# Thiophene-fused γ-lactams inhibit the SARS-CoV-2 main protease *via* reversible covalent acylation†

**DOI:** 10.1039/d4sc01027b

**Published:** 2024-04-16

**Authors:** Gayatri, Lennart Brewitz, Lewis Ibbotson, Eidarus Salah, Shyam Basak, Hani Choudhry, Christopher J. Schofield

**Affiliations:** a Chemistry Research Laboratory, Department of Chemistry and the Ineos Oxford Institute for Antimicrobial Research, University of Oxford 12 Mansfield Road OX1 3TA Oxford UK christopher.schofield@chem.ox.ac.uk lennart.brewitz@chem.ox.ac.uk; b Department of Biochemistry, Center for Artificial Intelligence in Precision Medicines, King Abdulaziz University Jeddah Saudi Arabia

## Abstract

Enzyme inhibitors working by *O*-acylation of nucleophilic serine residues are of immense medicinal importance, as exemplified by the β-lactam antibiotics. By contrast, inhibition of nucleophilic cysteine enzymes by *S*-acylation has not been widely exploited for medicinal applications. The SARS-CoV-2 main protease (M^pro^) is a nucleophilic cysteine protease and a validated therapeutic target for COVID-19 treatment using small-molecule inhibitors. The clinically used M^pro^ inhibitors nirmatrelvir and simnotrelvir work *via* reversible covalent reaction of their electrophilic nitrile with the M^pro^ nucleophilic cysteine (Cys145). We report combined structure activity relationship and mass spectrometric studies revealing that appropriately functionalized γ-lactams can potently inhibit M^pro^ by reversible covalent reaction with Cys145 of M^pro^. The results suggest that γ-lactams have potential as electrophilic warheads for development of covalently reacting small-molecule inhibitors of M^pro^ and, by implication, other nucleophilic cysteine enzymes.

## Abstract 2

Thiophene-fused γ-lactams are reversible covalent inhibitors of the SARS-CoV-2 main protease, a nucleophilic cysteine enzyme. γ-Lactams can inhibit nucleophilic cysteine enzymes by *S*-acylation as well as nucleophilic serine enzymes by *O*-acylation.

## Graphical abstract

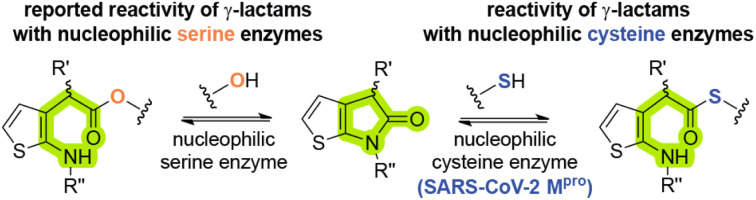

## Introduction

**Fig. 1:**
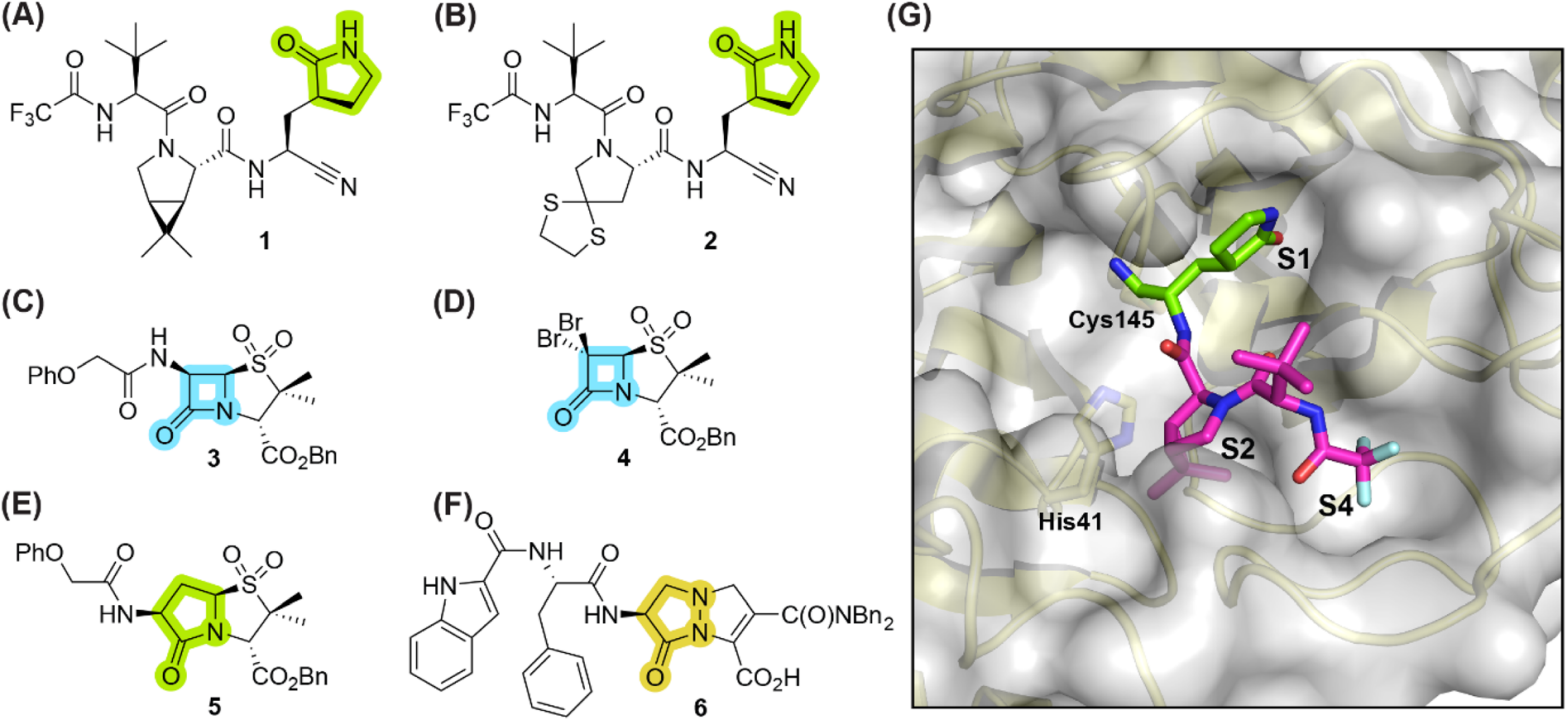
Selected reported γ-lactam- and β-lactam-containing SARS-CoV-2 main protease (M^pro^) inhibitors. (A) Nirmatrelvir (1);^14^ (B) simnotrelvir (2);^15^ (C) penicillin V sulfone benzyl ester 3;^21^ (D) β-lactam 4;^21^ (E) γ-lactam 5,^22^ derived from 3; (F) γ-lactam-derived pyrazolidinone 6;^23^ (G) view of the surface from the reported SARS-CoV-2 M^pro^:1 complex structure active site (PDB ID: 7TE0 ^24^), revealing that the γ-lactam group of 1 binds in the S1 subsite, whereas its bicyclic leucine mimic binds in the S2 subsite, its *tert*-butyl group is solvent exposed, and its trifluoroacetamide group binds in the S4 subsite. γ-Lactam, β-lactam, and pyrazolidinone groups are in green, blue, and ochre, respectively. Bn: benzyl; Ph: phenyl.

γ-Lactams are common in bioactive natural products,^1^ including *e.g.* in anantine and derivatives,^2,3^ monascuslactams A–D,^4^ the proteasome inhibitors lactacystin^5–7^ and salinosporamide A,^8^ and clausenamide.^9^ They are also present in clinically-used therapeutics, for example in the antiemetic rolapitant,^10^ the respiratory stimulant doxapram,^11^ piracetam, which is used to treat cortical myoclonus,^12^ the anti-cancer drug ivosidenib,^13^ and the antivirals nirmatrelvir (1)^14^ and simnotrelvir (2) (Fig. 1).^15^ The latter two inhibit the main protease (M^pro^) of the severe acute respiratory syndrome coronavirus type 2 (SARS-CoV-2),^16^ which catalyses hydrolysis of the viral polyproteins pp1a/1ab into functional non-structural proteins; M^pro^ inhibition results in impaired viral replication.^17–20^

**Fig. 2:**
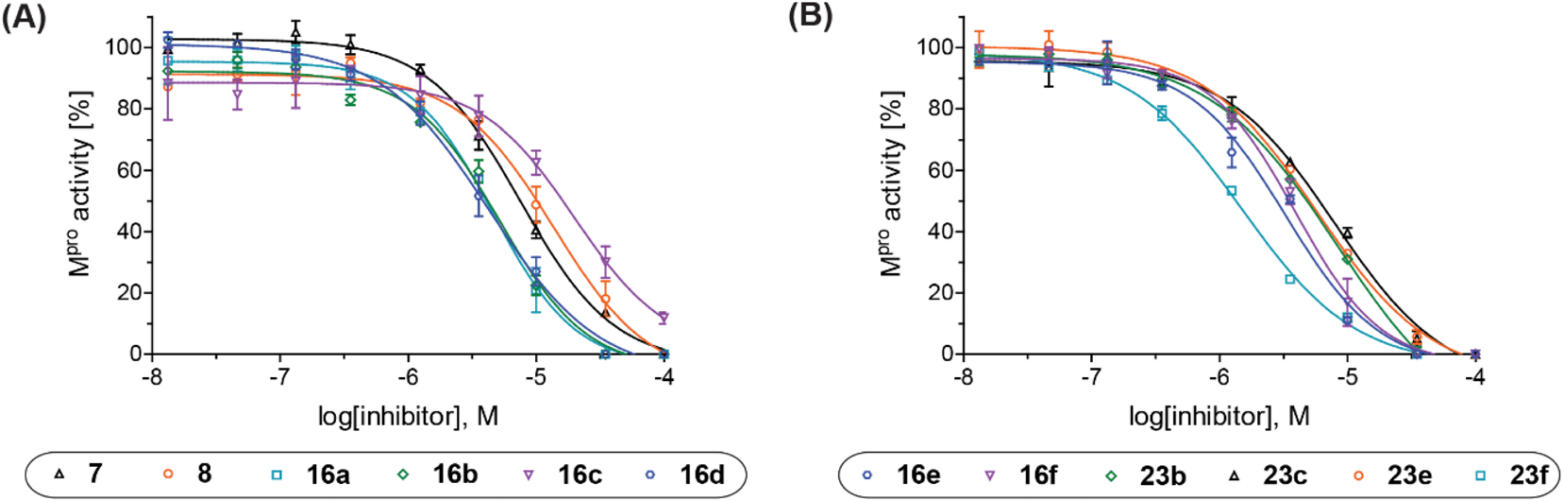
Representative dose–response curves of M^pro^ γ-lactam inhibitors used to determine IC_50_-values. (A) 7 (black triangles), 8 (orange circles), 16a (cyan boxes), 16b (green diamonds), 16c (violet inverse triangles), 16d (blue hexagons), and (B) 16e (blue hexagons), 16f (violet inverse triangles), 23b (green diamonds), 23c (black triangles), 23e (orange circles), 23f (cyan boxes). Two dose–response curves each composed of technical duplicates were independently determined using reported SPE-MS M^pro^ inhibition assays.^42^

Small-molecule inhibitors which acylate their target enzyme(s) *via* covalent reaction of a γ-lactam group with a nucleophilic residue have, to our knowledge, not yet been approved for therapeutic use. The lack of therapeutics which employ a γ-lactam as an electrophilic warhead for covalent reaction is remarkable, considering (i) the revived interest in the development of covalently reacting small-molecule therapeutics,^25,26^ (ii) recent advances in the synthesis of γ-lactams,^1,27,28^ (iii) the demonstrated safety of active pharmaceutical ingredients which contain a γ-lactam group that does not acylate the target enzyme, *e.g.*, nirmatrelvir (1)^14^ and simnotrelvir (2),^15^ and, in particular, (iv) that many clinically used small-molecules employ a β-lactam as an electrophilic warhead to acylate their target enzyme(s), *e.g.*, penicillin- and cephalosporin-based antibiotics.^29^ At least in part, this gap may reflect the reduced intrinsic reactivity of γ-lactams compared to more strained β-lactams based on (non-enzymatic) ring closure rates.^30^

The covalent reaction of γ-lactams with nucleophilic serine enzymes is reported;^31–39^ however, their analogous reactivity with nucleophilic cysteine enzymes, many of which are contemporary drug targets,^25,26^ is, to our knowledge, unknown. SARS-CoV-2 M^pro^ appears to be a suitable target to investigate the reactivity of γ-lactams with nucleophilic cysteine enzymes, because many small-molecule inhibitors are reported which employ an electrophilic group for reversible or irreversible covalent reaction with the nucleophilic thiolate of the catalytic cysteine residue of M^pro^ (*i.e.*, Cys145, Fig. 1) and because of the important structural roles of a γ-lactam group in many reported substrate-derived M^pro^ inhibitors,^40,41^*e.g.*, nirmatrelvir (1)^14^ and simnotrelvir (2).^15^ The γ-lactam group of these inhibitors binds in the S1 subsite of M^pro^, that is proximate to Cys145 (Fig. 1).

Of M^pro^ inhibitors that react covalently, those that react reversibly may be preferred over those that react irreversibly, as the latter may also react with ‘off-targets’ in an irreversible manner. Indeed Cys145 of M^pro^ reacts reversibly with the nitrile group of the clinically-used drugs 1 and 2.^14,15,42^ Many investigational M^pro^ inhibitors, however, employ highly reactive electrophiles for covalent reaction with Cys145, including *e.g.*, aldehydes, α-ketoamides, and Michael acceptors,^19,43,44^ which may potentially compromise safety, as reported for some clinically-used small-molecules bearing reactive electrophiles;^26,45^ The use of electrophilic groups with relatively low intrinsic reactivity is thus desirable. The observation that the γ-lactam of both 1 and 2 is stable in cells^14,15^ likely reflects its reduced reactivity compared to more reactive electrophiles, indicating that covalently reacting γ-lactams may have potential for development of safe COVID-19 therapeutics. However, by contrast with β-lactams,^21,22^ γ-lactams have, to our knowledge, not yet been considered as electrophilic warheads for covalent reaction with M^pro^ Cys145.

During the course of investigations aimed at developing penicillin-based M^pro^ inhibitors which acylate Cys145 *via* β-lactam ring opening (*e.g.*, 3 and 4),^21,22^ we synthesized the γ-lactam analogue 5 to probe the effect of altering the lactam group on potency, including with respect to reversibility of acylation. Consistent with studies revealing that acylation of nucleophilic serine residues is more reversible with a γ-lactam compared to an analogous β-lactam,^46,47^ γ-lactam 5 inhibits isolated recombinant SARS-CoV-2 M^pro^ ∼4-fold less efficiently than the structurally-related β-lactam 3.^22^ Mass spectrometric analyses indicated that, by contrast with β-lactam 3, γ-lactam 5 did not react to form a stable acyl–enzyme complex, suggesting that it may bind to M^pro^ principally *via* non-covalent interactions.^22^

Here we report the synthesis of thiophene-fused γ-lactams which inhibit isolated recombinant SARS-CoV-2 M^pro^ more efficiently than β-lactam 3 and γ-lactam 5 (Fig. 1). Mass spectrometric evidence supports a mechanism involving reversible covalent reaction of the γ-lactam group with Cys145. The results reveal bicyclic γ-lactams are useful scaffolds for the inhibition of nucleophilic cysteine enzymes.

## Results

### Thiophene-fused γ-lactams inhibit SARS-CoV-2 M^pro^

Natural product inspired *trans*-ring-fused γ-lactams can inhibit serine proteases *via* acylating their nucleophilic serine residue,^31,32^ as is also the case for thiophene-fused γ-lactams.^33^ In the latter case, it is proposed that, following acylation, the presence of the thiophene ring hinders deacylation by sequestering electron density on the γ-lactam-derived amine.^33^ Based on the proposal that a hydrophobic thiophene ring may bind in the hydrophobic S2 pocket of M^pro^ that is in proximity of Cys145 (Fig. 1), we synthesized an initial set of thiophene-fused γ-lactams (7–9) following modifications of reported procedures.^33^ The effect of these synthetic γ-lactams on catalysis of isolated recombinant SARS-CoV-2 M^pro^ was investigated using solid phase extraction coupled to mass spectrometry (SPE-MS) based assays, which directly monitor M^pro^-catalysed hydrolysis of a pp1a/1ab-derived oligopeptide,^21,22,42^ and which we and others have used to characterise covalently and non-covalently binding M^pro^ inhibitors.^21,22,42,48–56^

Analysis of the half-maximum inhibitory concentrations (IC_50_-values) revealed that both the regioisomeric thiophene-fused γ-lactams 7 and 8 moderately inhibited isolated recombinant SARS-CoV-2 M^pro^ with similar potencies (IC_50_ ∼ 8.5 and 8.4 μM, respectively; Table 1, entries i and ii). By contrast, the regioisomeric γ-lactam 9 did not inhibit M^pro^ over the tested concentration range (Table 1, entry iii), showing that the position of the thiophene sulfur atom with respect to the γ-lactam nitrogen atom affects inhibition potency. The substitution of the methylene group of 9 with an NSO_2_Me group to give 10 did not result in inhibition (Table 1, entry iv). To investigate the effect of the thiophene ring of 7 and 8, we synthesized the corresponding benzene-fused γ-lactam 11 using the route employed for synthesis of 7 and 8. 11 did not inhibit M^pro^ over the tested concentration range (Table 1, entry v), indicating that the size of the ring fused to the γ-lactam, nature of delocalization, and/or the presence of a sulfur atom in that ring are important for the inhibition manifested by 7 and 8.

**Table 1:** Thiophene-fused γ-lactams inhibit isolated recombinant SARS-CoV-2 M^pro^

	γ-Lactam	aIC_50_ [μM]
i	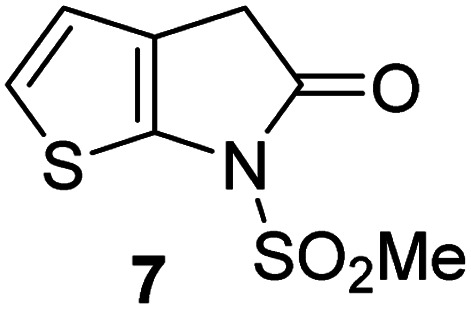	8.5 ± 1.4
ii	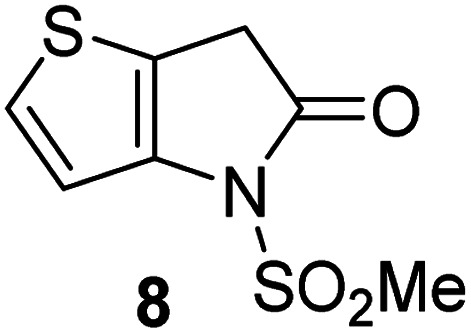	8.4 ± 0.5
iii	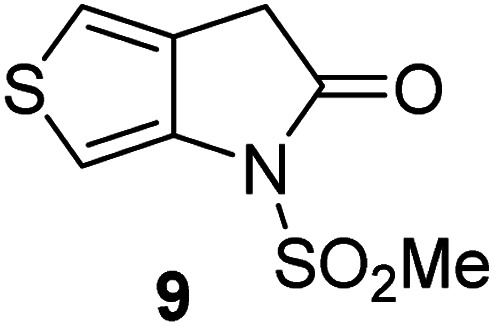	>100
iv	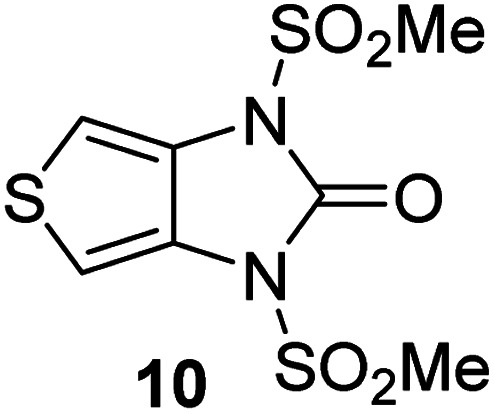	>100
v	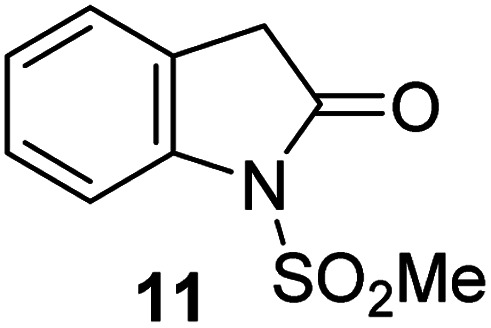	>100

a Assays were performed as reported using SPE-MS, employing SARS-CoV-2 M^pro^ (0.05 μM) and substrate peptide (2.0 μM).^42^ Results are means of two independent runs each composed of technical duplicates (*n* = 2; mean ± standard deviation, SD). Representative dose–response curves of selected γ-lactams are shown in Fig. 2.

The M^pro^ inhibition potency of γ-lactams 7 and 8 is in the range of that reported for penicillin V sulfone benzyl ester 3 (IC_50_ ∼ 6.6 μM;^22^Fig. 1), which inhibits M^pro^*via* covalent reaction of its β-lactam with the active site Cys145.^22^ Notably, γ-lactams 7 and 8 inhibit M^pro^ ∼3-fold more efficiently than the reported γ-lactam 5 (IC_50_ ∼ 26.1 μM;^22^Fig. 1), which inhibits M^pro^ apparently *via* non-covalent interactions,^22^ and ∼5-fold more efficiently than the reported pyrazolidinone 6 (IC_50_ ∼ 45 μM;^23^Fig. 1).

### Substitution affects the inhibition potency of thiophene-fused γ-lactams

**Scheme 1:**
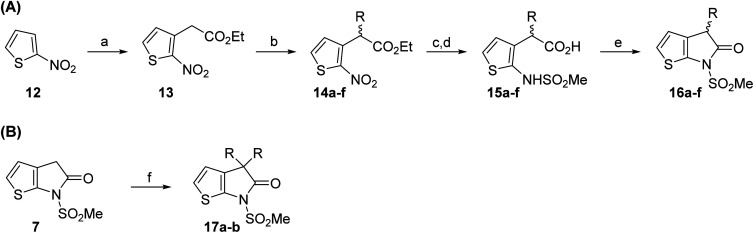
Synthesis of α-substituted γ-lactam derivatives of 7. Reagents and conditions: (a) ethyl chloroacetate, KO^*t*^Bu, THF, −50 °C to rt, 89%; (b) Cs_2_CO_3_, alkyl halide, DMF, rt, 52–94%, or for 14e: 1,1,3,3-tetramethylguanidine (20 mol%), acrylonitrile, THF, rt, 55%; (c) Fe(0), FeSO_4_ (8 mol%), dioxane : H_2_O (4 : 1), reflux; then: methylsulfonyl chloride, NEt_3_, 4-(*N*,*N*-dimethylamino)pyridine (10 mol%), CH_2_Cl_2_, rt, 10–30%; (d) LiOH, THF : H_2_O : EtOH (2 : 1 : 1), rt, 73–94%; (e) 1-[bis(dimethylamino)methylene]-1*H*-1,2,3-triazolo[4,5-*b*]pyridinium-3-oxide hexafluorophosphate (HATU),^58^^*i*^Pr_2_NEt,^59^ MeCN : CH_2_Cl_2_ (1 : 1), rt, 46–76%; (f) Cs_2_CO_3_ (2.5 equiv.), alkyl halide (2.2 equiv.), DMF, rt, 9–90%. See Table 2 for structures of 16a–f and 17a–b.

Structure activity relationship studies were performed to investigate whether the lactam nitrogen substituent and substituents α to the lactam carbonyl affect inhibition potency. Derivatives of γ-lactam 7 with a single α substituent were synthesized from commercially-sourced 2-nitrothiophene (12) in 5 steps following modification of reported procedures (Scheme 1A).^33^ Initially, 12 was efficiently alkylated with ethyl chloroacetate to give thiophene 13 as a single regioisomer. 13 was alkylated using an alkyl halide with Cs_2_CO_3_ as a base to give thiophenes 14a–d and 14f; 14e was synthesized from 13 using catalytic amounts of 1,1,3,3-tetramethylguanidine as a base and acrylonitrile as a Michael acceptor, as reported for related nitriles.^57^ Nitrothiophenes 14a–f were converted to the corresponding sulfonamides 15a–f following nitro-reduction, sulfonylation, and saponification. γ-Lactams 16a–f were obtained from 15a–f*via* HATU^58^-mediated amide bond formation. Derivatives of γ-lactam 7 which bear two identical α substituents, *i.e.*, 17a and 17b, were directly synthesized from 7*via* an alkylation reaction (Scheme 1B).

The M^pro^ inhibition results reveal that the addition of a methyl group α to the carbonyl of γ-lactam 7 increases inhibition potency by ∼2-fold, whereas the addition of a second methyl group ablates inhibition (Table 2, entries i and ii). The length of the alkyl substituent α to the γ-lactam carbonyl did not appear to substantially affect potency: 16b, which bears a propyl substituent α to the lactam carbonyl, inhibited isolated recombinant SARS-CoV-2 M^pro^ with similar potency as 16a which bears a methyl group at the same position (Table 2, entries i and iii). By contrast, isomeric isopropyl-substituted γ-lactam 16c inhibited M^pro^ ∼5-fold less efficiently than propyl-substituted 16b (IC_50_ ∼ 16 μM; Table 2, entry iv). However, sterically bulky substituents are not necessarily detrimental for efficient inhibition, since benzyl substituted γ-lactam 16d inhibited with similar potency as 16a and 16b (IC_50_ ∼ 4.0 μM; Table 2, entry v).

**Table 2:** Effect of substituents α to the γ-lactam carbonyl on M^pro^ inhibition

	γ-Lactam	aIC_50_ [μM]		γ-Lactam	aIC_50_ [μM]
i	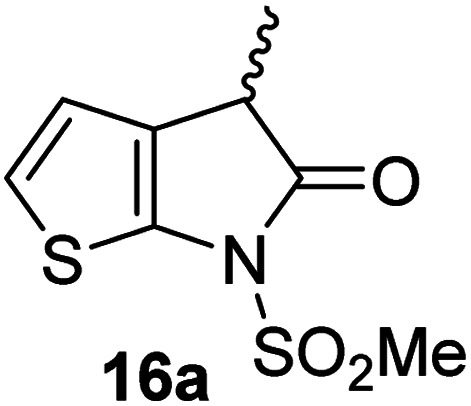	3.9 ± 0.1	vi	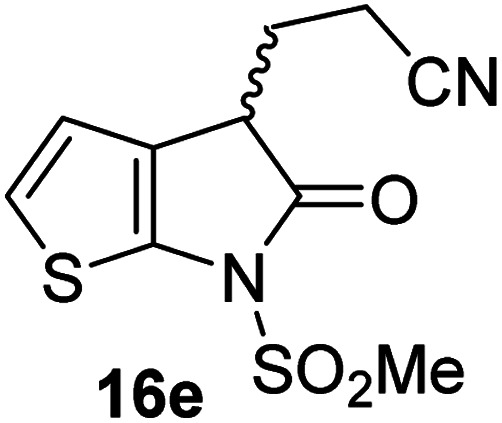	2.7 ± 0.1
ii	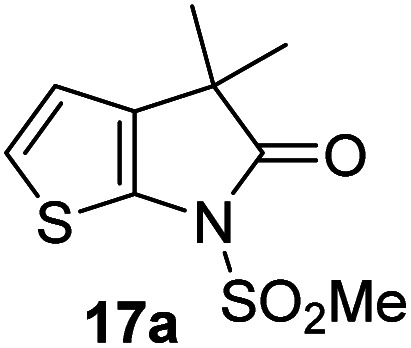	>100	vii	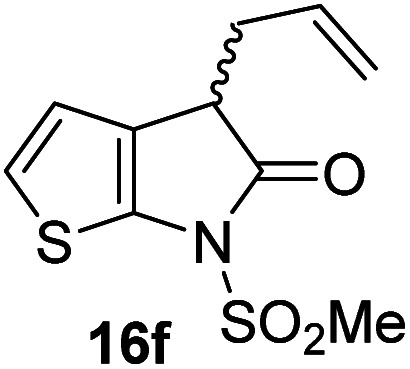	3.5 ± 0.2
iii	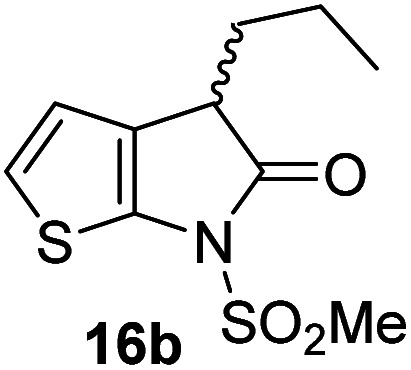	3.2 ± 0.8	viii	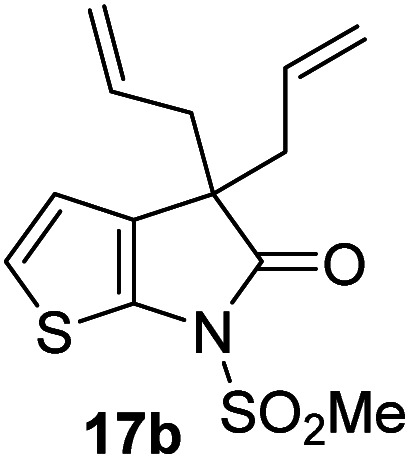	>100
iv	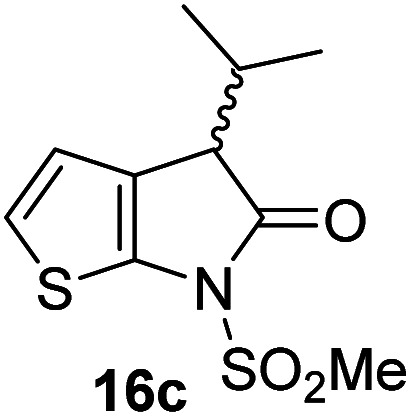	16 ± 2	ix	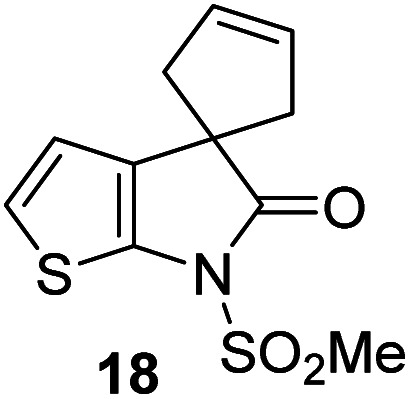	61 ± 4
v	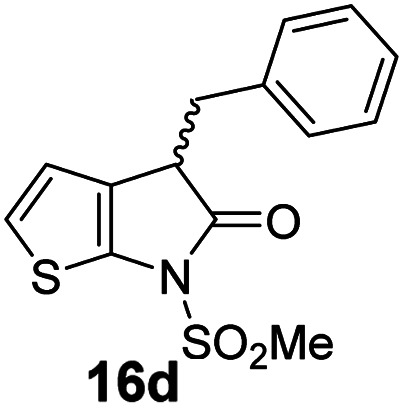	4.0 ± 0.4	x	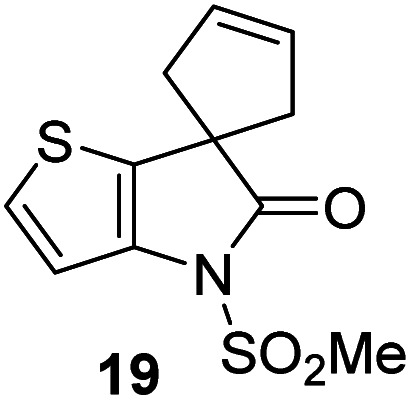	>100

a Assays were performed as reported using SPE-MS, employing SARS-CoV-2 M^pro^ (0.05 μM) and substrate peptide (2.0 μM).^42^ Results are means of two independent runs each composed of technical duplicates (*n* = 2; mean ± SD). Representative dose–response curves of selected γ-lactams are shown in Fig. 2.

γ-Lactams 16e and 16f which are both derived from propyl-substituted γ-lactam 16b, but which contain a nitrile or olefin, respectively, in their alkyl substituent, inhibited M^pro^ with similar potency to 16b (IC_50_ ∼ 2.7 and 3.5 μM, respectively; Table 2, entries vi and vii). Similar to α-disubstituted γ-lactam 17a, 17b which bears two allyl substituents α to its lactam carbonyl did not inhibit M^pro^ (Table 2, entry viii). Both the spiro γ-lactam 18, which was synthesized from 17b*via* a ring-closing metathesis,^60^ and the isomeric γ-lactam 19, which was synthesized from γ-lactam 8 following an analogous synthesis route (ESI†), did not efficiently inhibit M^pro^ (Table 2, entries ix and x).

**Scheme 2:**
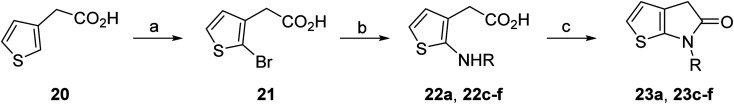
Synthesis of γ-lactams bearing different lactam nitrogen substituents. Reagents and conditions: (a) *N*-bromosuccinimide, THF, 0 °C to rt, 80%; (b) amine, K_2_CO_3_, ^*t*^BuOH, CuI (10 mol%), *N*,*N*′-dimethylethylenediamine, 100 °C; or: amine, K_2_CO_3_, Cu(0), pyridine, 120 °C, 5–44%; (c) HATU,^58^^i^Pr_2_NEt,^59^ MeCN : CH_2_Cl_2_ (1 : 1), rt, 22–67%. See Table 3 for structures of 23a and 23c–f.

The effect of varying the γ-lactam nitrogen substituent of 7 on inhibition potency was investigated (Table 3). Derivatives of 7, *i.e.*, 23a and 23c–f, were synthesized from commercially-sourced 2-(thiophen-3-yl)acetic acid (20) in 3 steps, employing copper-catalysed reaction of thiophene bromide 21 with activated amines (Scheme 2).^61,62^ γ-Lactam 23b was synthesized from thiophene 13 in a similar manner to which 7 was prepared (ESI†). Substituting the methylsulfonyl group of γ-lactam 7 for an acetyl group ablated M^pro^ inhibition (Table 3, entry ii), whereas use of benzylsulfonyl or phenylsulfonyl groups apparently increased potency by ∼2-fold (Table 3, entries iii and iv). In some cases, the addition of substituents on the phenyl ring of 23c*para* to the sulfonyl group further increased potency (Table 3, entries v–vii); the CF_3_-substituted γ-lactam 23f appeared to be a particularly potent M^pro^ inhibitor, being ∼3- and ∼6-fold more potent than γ-lactams 23c and 7, respectively (IC_50_ ∼ 1.3 μM; Table 3, entry vii). Notably, γ-lactam 23f inhibits M^pro^ ∼20-fold more efficiently than our reported non-covalently reacting γ-lactam M^pro^ inhibitor 5 and ∼6-fold more efficiently than our reported covalently reacting β-lactam M^pro^ inhibitor 3 (Fig. 1).^22^

**Table 3:** The γ-lactam nitrogen substituent affects M^pro^ inhibition

	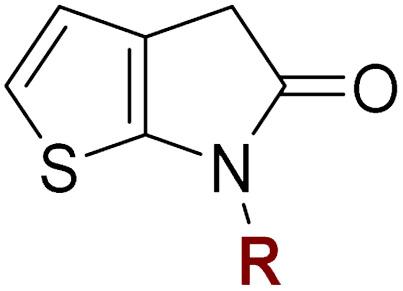	aIC_50_ [μM]
i	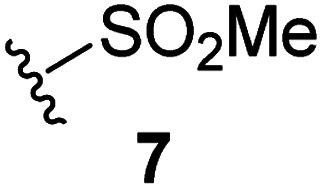	8.5 ± 1.4
ii	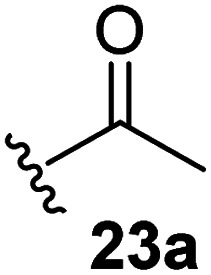	>100
iii	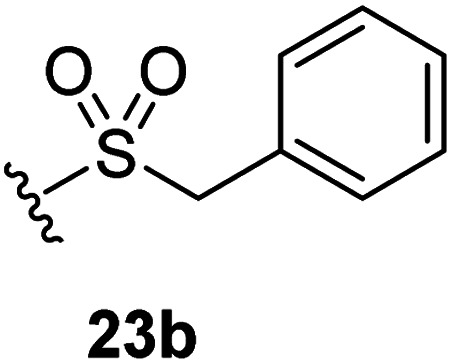	4.3 ± 0.1
iv	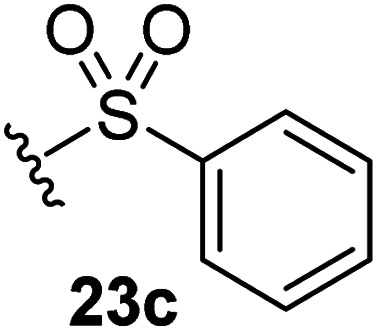	4.7 ± 1.1
v	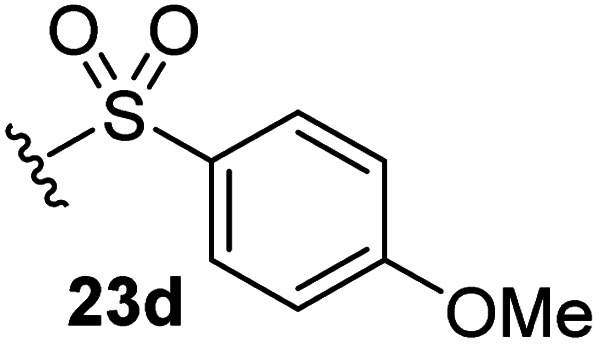	2.7 ± 0.2
vi	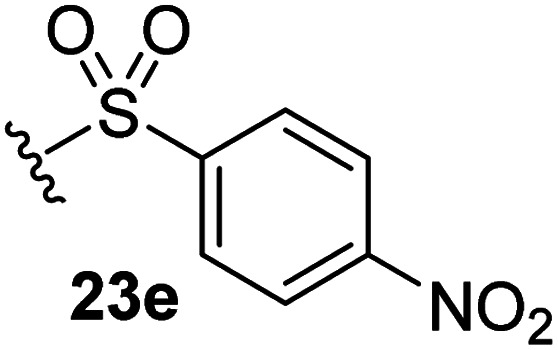	4.4 ± 0.7
vii	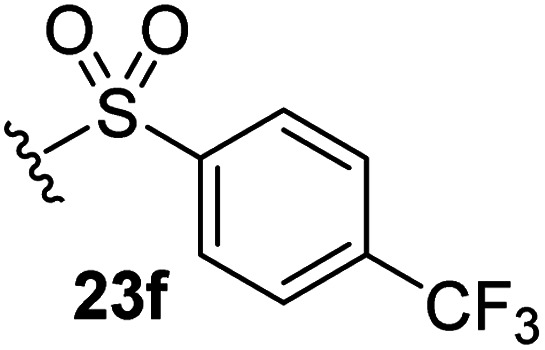	1.3 ± 0.1

a Assays were performed as reported using SPE-MS, employing SARS-CoV-2 M^pro^ (0.05 μM) and substrate peptide (2.0 μM).^42^ Results are means of two independent runs each composed of technical duplicates (*n* = 2; mean ± SD). Representative dose response curves of selected γ-lactams are shown in Fig. 2.

### Thiophene-fused γ-lactams inhibit M^pro^*via* reversible covalent reaction

**Fig. 3:**
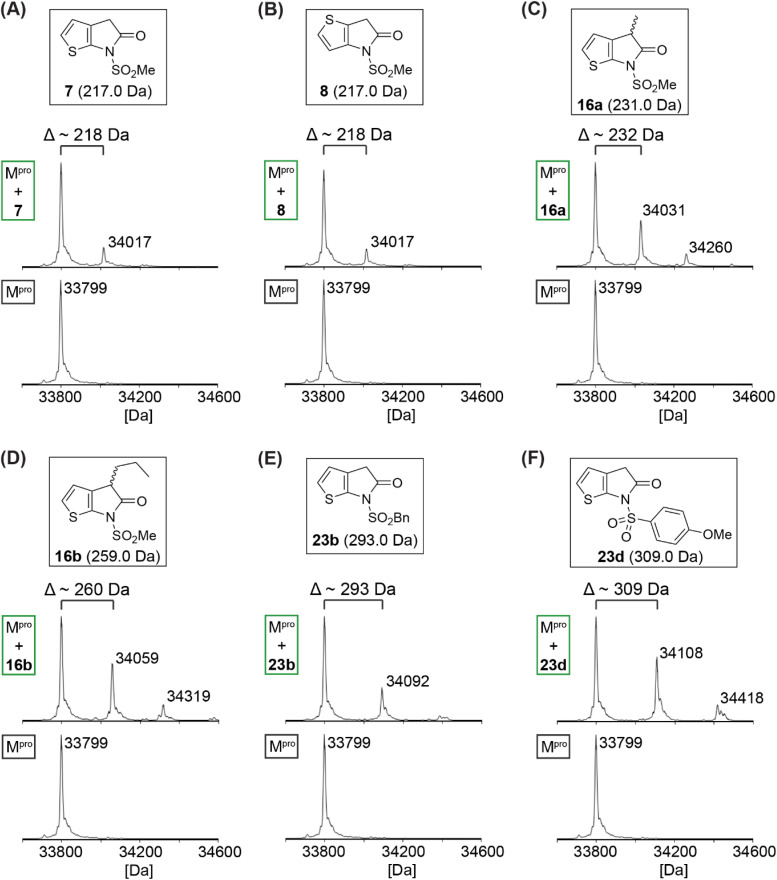
γ-Lactams react covalently with isolated recombinant M^pro^. Analysis of a reaction mixture of M^pro^ and γ-lactams (A) 7, (B) 8, (C) 16a, (D) 16b, (E) 23b, and (F) 23d prior (bottom) and 4 h post (top) incubation with the respective γ-lactam. Assays were performed using SPE-MS as described in the Experimental section employing SARS-CoV-2 M^pro^ (3.0 μM) and, if appropriate, a γ-lactam (15 μM) in buffer (20 mM HEPES, pH 7.5). Representative spectra of technical duplicates are shown.

Protein-observed MS studies under denaturing conditions were performed with selected synthetic γ-lactams to investigate whether they inhibit isolated recombinant SARS-CoV-2 M^pro^*via* non-covalent interactions, as for γ-lactam 5,^22^ or *via* covalent reaction. The results reveal that some of the tested γ-lactams covalently react with M^pro^ during the tested time period (*i.e.*, 4 h), as shown by the anticipated mass shifts (Fig. 3); the stoichiometry of the covalent reaction appears to be, at least predominantly, 1 : 1, suggesting that γ-lactams react selectively with a single nucleophilic M^pro^ residue, likely Cys145. Nonetheless, the MS studies imply that some of the γ-lactams, *i.e.*, 16a, 16b, and 23d, may have capacity to covalently react with M^pro^ residues other than Cys145, albeit at substantially lower levels even when being used in excess; note that M^pro^ has eleven cysteine residues in addition to Cys145, all of which can covalently react with non-specific inhibitors such as ebselen.^49^

Variable levels of M^pro^ acylation were observed depending on the γ-lactam employed, suggesting that initial binding constants, reaction rates, and/or stabilities of the acyl–enzyme complex differ depending on the substitution pattern. Notably, complete M^pro^ acylation was not observed under the tested conditions, an observation which may reflect the reversibility of the reaction and the comparatively low enzyme to γ-lactam ratio employed in the assay (*i.e.*, 1 : 5); this ratio was chosen to avoid γ-lactam-induced ionization suppression of M^pro^ observed at higher γ-lactam concentrations, thus perturbing data analysis.

**Fig. 4:**
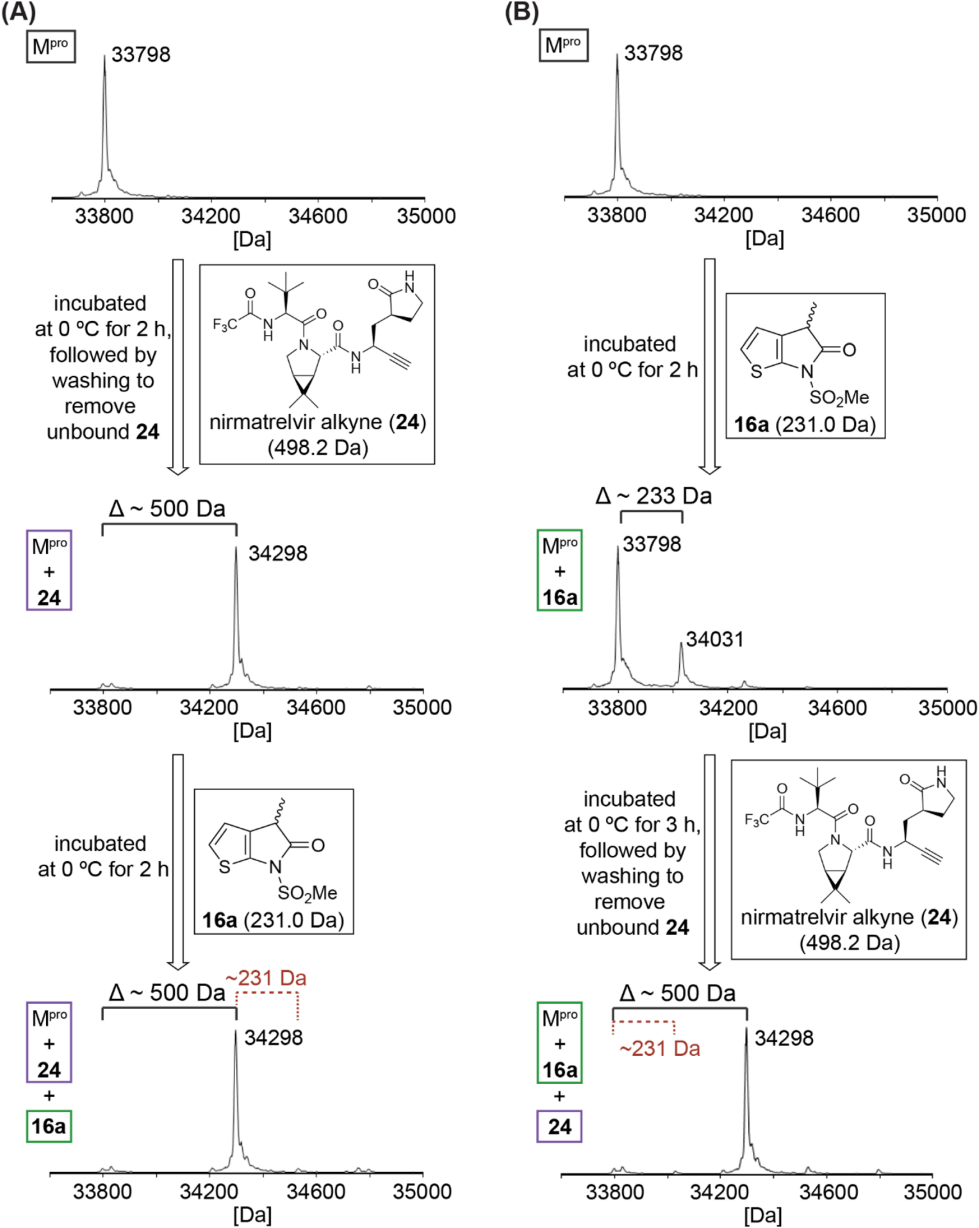
Evidence that γ-lactam 16a inhibits M^pro^ by selective reversible covalent reaction with Cys145. (A) γ-Lactam 16a does not covalently react with the covalent M^pro^:nirmatrelvir alkyne derivative 24^42^ complex obtained *via* irreversible covalent reaction of M^pro^ Cys145 with 24,^42^ indicating that γ-lactams selectively react with the nucleophilic thiolate of Cys145 under the tested conditions; (B) addition of an excess of 24^42^ to a mixture containing the covalent M^pro^:16a complex results in stoichiometric formation of the corresponding covalent M^pro^:24^42^ complex, substantial levels of the M^pro^:16a complex were not detected using SPE-MS implying that the reaction of γ-lactams with M^pro^ is reversible and/or that the acyl–enzyme complex is not stable towards hydrolysis. Assays were performed using SPE-MS, as described in the Experimental section, employing SARS-CoV-2 M^pro^ (3.0 μM), and, if appropriate, γ-lactam 16a (15 μM) and/or nirmatrelvir alkyne 24^42^ (50 μM) in buffer (20 mM HEPES, pH 7.5). Representative spectra of technical duplicates are shown.

To localize the site of covalent modification and to probe whether covalent reaction occurs with Cys145, 16a was incubated with M^pro^ that had been previously reacted with a small-molecule inhibitor^42^ that selectively and irreversibly reacts with Cys145 (Fig. 4); 16a was used for this study because its levels of M^pro^ acylation were apparently higher than those for the unsubstituted 7 and 8, and because it has the least bulky substituent amongst those γ-lactams that covalently react with M^pro^, a property which may favour more efficient covalent reaction. We have reported that an alkyne derivative of nirmatrelvir (24; Fig. 4), in which the electrophilic nitrile is substituted for an alkyne, reacts selectively and irreversibly with Cys145.^42^ Thus, isolated recombinant SARS-CoV-2 M^pro^ was incubated with a ∼16-fold excess of the alkyne derivative of 1 (*i.e.*, 24) to block the thiol of Cys145 by stoichiometric thioenol ether formation (Fig. 4A).^42^ The excess of 24 was removed by washing and the resultant covalent M^pro^:24 complex was incubated with 16a. The results reveal that 16a does not covalently react with the covalent M^pro^:nirmatrelvir alkyne (24)^42^ complex within the tested time (*i.e.*, 2 h). γ-Lactam 16a thus likely reacts selectively under the tested conditions with Cys145, but not, at least substantially, with other surface-exposed cysteine residues of M^pro^.

It was of interest to investigate whether the synthetic γ-lactams react reversibly with Cys145. Thus, γ-lactam 16a was incubated with M^pro^ at 0 °C for 2 h (enzyme/16a ratio: 1 : 5), before the nirmatrelvir alkyne derivative 24 was added to the reaction mixture. The resultant mixture was incubated for 3 h at 0 °C, followed by washing to remove excess 24 and analysis using SPE-MS (Fig. 4B). Stoichiometric formation of the covalent M^pro^:24 (ref. 42) complex was observed, whereas substantial levels of the M^pro^:16a complex were not detected (Fig. 4B). The results imply that the reaction of γ-lactams with M^pro^ is reversible and/or that the resultant acyl–enzyme complex is not stable towards hydrolysis. This proposal is precedented by the reported hydrolytic γ-lactamase activity of other nucleophilic serine^63,64^ and cysteine enzymes.^65–67^ The combined results indicate that γ-lactams have potential as electrophilic warheads for development of covalently reacting small-molecule inhibitors of M^pro^ and, consequently, other nucleophilic cysteine enzymes.

## Discussion

Penicillins and related antibiotics inhibit bacterial cell wall biosynthesis *via* covalent reaction of their electrophilic β-lactam ring with the nucleophilic serine residue of transpeptidases to give stable acyl-enzyme complexes.^68^ Efforts to substitute the β-lactam ring of penicillins began in the 1940s, ultimately leading to the identification of suitably activated γ-lactams and related compounds, including the natural product lactivicin and derivatives, as antibiotics.^69–75^ Subsequently, 1,6-diazabicyclo[3.2.1]octane-based compounds (DBOs),^76–79^ including avibactam, have been developed for clinical use as reversibly reacting covalent inhibitors of nucleophilic serine β-lactamases.^80–82^

γ-Lactams have been developed as inhibitors of both human and viral serine proteases including *e.g.*, human neutrophil elastase,^31–33^ the hepatitis C virus (HCV) ns3/4a serine protease,^34–37^ and the human cytomegalovirus (HCMV) serine protease.^38,39^ They inhibit *via* acylation of the nucleophilic serine residue, at least in some cases in a reversible manner.^38,46^ By contrast with β-lactams,^21,22,83–89^ the reaction of γ-lactams with nucleophilic cysteine enzymes has not, to our knowledge, been well explored. Several γ-lactamases have been proposed to catalyse γ-lactam hydrolysis *via* nucleophilic attack by a cysteine residue,^65–67^ however, the intermediate acyl-enzyme complexes have not yet been structurally characterized.

Our combined results imply that γ-lactams have potential to be useful covalently reacting inhibitors of nucleophilic cysteine enzymes, in particular SARS-CoV-2 M^pro^. They reveal that thiophene-fused γ-lactams can efficiently inhibit M^pro^*in vitro* (Tables 1–3), in accord with the proposal that the thiophene ring helps to sequester electron density of the γ-lactam-derived amine following acyl–enzyme complex formation.^33^ The γ-lactam thiophene ring appears to be important for efficient M^pro^ inhibition, since its substitution by a benzene ring ablates inhibition and, interestingly, the regioisomeric positioning of the sulfur atom within the thiophene ring also affects inhibition potency (Table 1). The reasons for this observation, including precisely how sequestration of the lone pair(s) on the γ-lactam-derived amine *N* atom affects the extent of inhibition, are under investigation. The results also show that substitution both α to the γ-lactam carbonyl and on the γ-lactam *N* atom affect inhibition potency (Tables 2 and 3). Thus, there is considerable scope for further optimization of the identified γ-lactam M^pro^ inhibitors, in particular with respect to optimal binding in the S1 or S2 pocket. The knowledge that γ-lactams can bind in the S1 pocket of M^pro^*via* non-covalent interactions^14,24,42^ suggests that derivatives of 7 and 8 possessing a second γ-lactam binding in the S2 or S1 pocket are of interest.

**Fig. 5:**
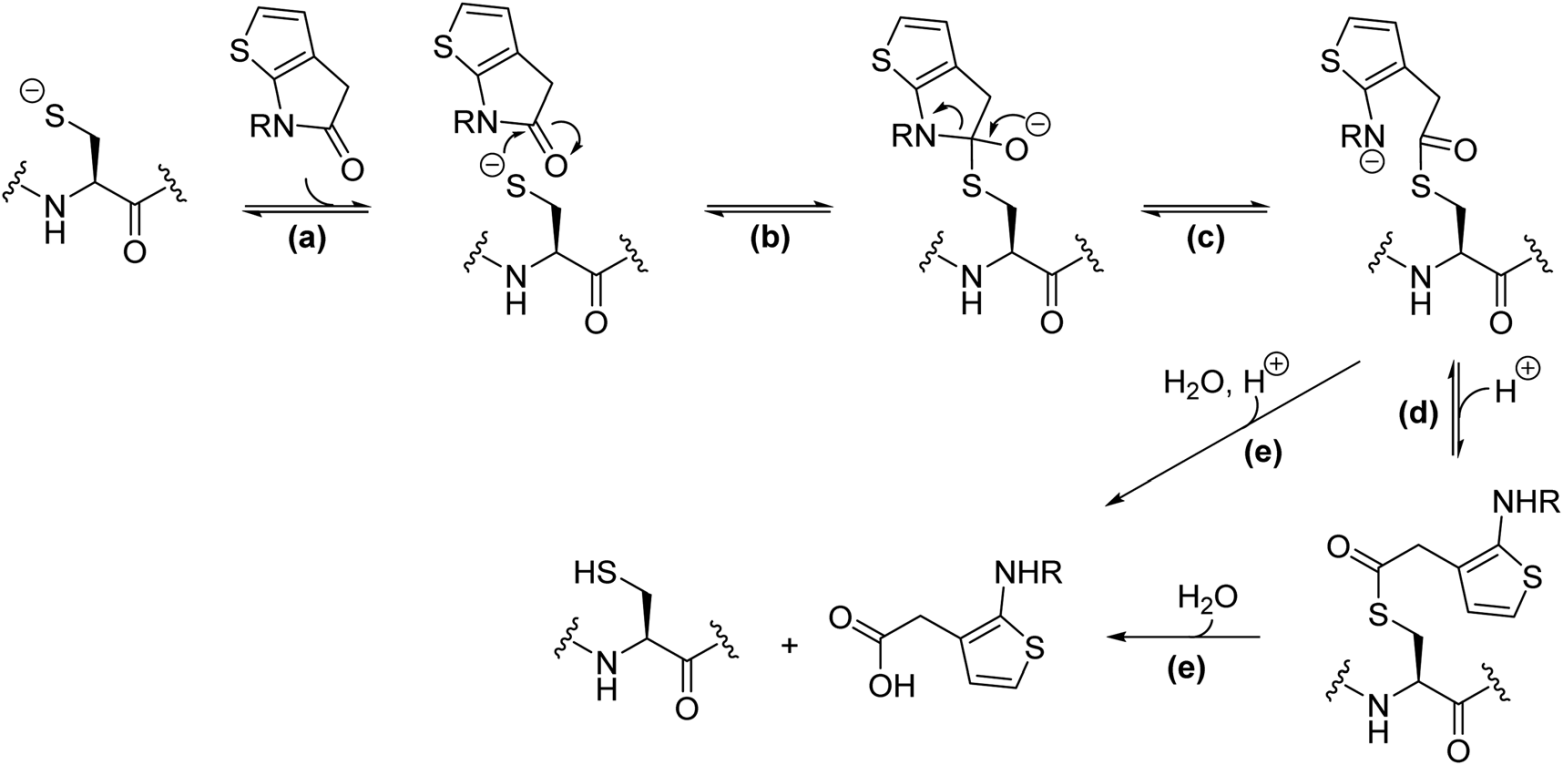
Proposed scheme for reaction of thiophene-fused γ-lactams with nucleophilic cysteine enzymes. Reaction steps include: (a) the reversible non-covalent binding of the thiophene-fused γ-lactam to the enzyme active site; (b) reversible nucleophilic attack of the cysteine thiolate to the proximate γ-lactam carbonyl forming a tetrahedral intermediate; (c) reversible γ-lactam fission and formation of an acyl–enzyme complex; (d) (reversible) protonation of the resultant amine, which may or may not be associated with a conformational change to form a hydrolytically more stable acyl–enzyme complex; (e) irreversible hydrolysis of the acyl–enzyme complex. Note there is variation in the general acid base machinery and oxy-anion stabilising mechanisms employed by nucleophilic cysteine enzymes.

Our MS studies reveal that γ-lactams acylate the nucleophilic thiolate of Cys145 in a reversible manner (Fig. 3–5). The results thus indicate that lactam rings other than β-lactams have potential for development as covalently reacting inhibitors of M^pro^ and, by implication, of other nucleophilic cysteine enzymes. However, it should be noted that γ-lactams do not necessarily have to covalently react with M^pro^ for efficient inhibition, because *e.g.*, γ-lactam 5 (Fig. 1) is reported to likely inhibit M^pro^ without covalently reacting.^22^ The formation of a γ-lactam from an ester, including an acyl–enzyme complex, is intrinsically more favourable than that of a β-lactam, as indicated by studies on the reactivity of γ-lactams with a nucleophilic serine enzyme.^30,46^ The more reversible nature of γ-lactam *versus* β-lactam reaction with nucleophilic residues may, in some circumstances, be an advantage with respect to limiting (irreversible) off-target reactivity. Reversibility may, however, be an unfavourable property with respect to the relative potency of inhibition for analogous γ- and β-lactams.

It is possible that the γ-lactam-derived acyl–enzyme complex can either react to reform the initial γ-lactam and/or be hydrolysed (Fig. 5), as proposed for other nucleophilic cysteine enzymes with reported γ-lactamase reactivity.^65–67^ We did not observe evidence for γ-lactam hydrolysis in our MS studies; this potential problem can be limited by steric extrusion of hydrolytic water from the active site, as precedented with work on inhibiting nucleophilic serine enzymes.^33,46,47^ The potential γ-lactam liability concerning reversibility in acylation can be overcome if the amine lone pair derived from the γ-lactam is sequestered in the acyl–enzyme complex; indeed, this concept enabled the initial development of thiophene-fused γ-lactams as serine protease inhibitors.^33^ It should be noted that the acylation ability of 5,5-*trans*-fused bicyclic pyrrolidine lactams^31^ and related γ-lactams is likely not, at least principally, a result of their ring strained structure.^90^ Indeed, the normally efficient natural substrates of proteases are themselves unstrained amides. Hence, empirical optimization remains of importance in inhibitor development.

We have not yet obtained a crystal structure of the acyl–enzyme complex formed by covalent reaction of Cys145 with a γ-lactam, something that may reflect reversible binding and/or the labile nature of this intermediate. In addition to inhibiting by covalent reaction with Cys145, our combined mass spectrometric evidence imply that γ-lactams can also inhibit M^pro^*via* non-covalent binding.^22^ It is thus possible that γ-lactams can bind to the M^pro^ active site in different conformations, *i.e.*, one that enables covalent reaction of the γ-lactam group with Cys145 or one that enables non-covalent binding of the γ-lactam group, for example in the S1 pocket, as crystallographically observed for the γ-lactam of nirmatrelvir (1).^14,24^ Our current work is thus focused on substituents α to the γ-lactam carbonyl which engage with residues at the M^pro^ active site to promote formation of a stable acyl–enzyme complex.

## Conclusions

Our results expand the repertoire of covalently reacting groups for efficient SARS-CoV-2 M^pro^ inhibition to γ-lactams and suggest that γ-lactams may also covalently react with other disease-relevant nucleophilic cysteine enzymes. In this regard, it will be of interest to investigate γ-lactams as inhibitors of nucleophilic cysteine enzymes from different mechanistic sub-families, including the SARS-CoV-2 papain-like protease (PL^pro^), which employs a catalytic triad for catalysis, rather than a dyad as M^pro^.^91–93^ Considering that both β-lactams^21,22^ and, as we now report, γ-lactams can covalently react with Cys145, other related ring systems also have potential for M^pro^ inhibition. Such ring systems include lactone derivatives, as precedented by work on β-lactone inhibitors of hepatitis A virus and plant nucleophilic cysteine enzymes.^94–97^ The corresponding β- and γ-sultam derivatives and δ-lactams, which are reported to inhibit human neutrophil elastase,^98–100^ may also be suited to covalent reaction with nucleophilic cysteine enzymes, including M^pro^.

## Experimental section

### γ-Lactam synthesis

Thiophene-fused γ-lactams were synthesized following modifications of reported procedures.^33^

### SARS-CoV-2 Mpro inhibition assays

Solid phase extraction coupled to mass spectrometry (SPE-MS) inhibition assays were performed using isolated recombinant SARS-CoV-2 M^pro^ (0.05 μM), which was based on the Wuhan-Hu-1 genome^101^ (National Center for Biotechnology Information (NCBI) reference sequence: NC_045512.2) and which was prepared according to established procedures,^21^ a 37mer oligopeptide (ALNDFSNSGSDVLYQPPQTSITSAVLQ/SGFRKMAFPS-NH_2_), which was based on the on the sequence of the N-terminal SARS-CoV-2 M^pro^ self-cleavage site and synthesized as a C-terminal amide and purified by GL Biochem (Shanghai) Ltd (Shanghai, China), as a substrate (2.0 μM), and the N-terminally acetylated C-terminal product peptide (Ac-SGFRKMAFPS-NH_2_) as an internal standard (0.4 μM) in buffer (20 mM HEPES, pH 7.5, 50 mM NaCl, 20 °C), as reported.^42^

### Protein-observed Mpro assays

Assays were performed as reported using recombinant isolated SARS-CoV-2 M^pro^ (3.0 μM) and, if appropriate, the indicated γ-lactam (15 μM) and/or nirmatrelvir alkyne 24^42^ (50 μM) in buffer (20 mM HEPES, pH 7.5, 20 °C); reaction mixtures were analysed using SPE-MS.^21,22,42^

## Data availability

The synthetic procedures and the characterization of all products and NMR spectra of the lead inhibitors are given in the ESI.†

## Author contributions

G. and L. I. synthesised the γ-lactams with assistance from S. B.; L. B. performed assays; E. S. and H. C. provided resources; L. B. and C. J. S. supervised and conceived the research and wrote the manuscript with help from G.

## Conflicts of interest

The authors declare no competing interests.

## Supplementary Material

SC-015-D4SC01027B-s001
