# Supplementary material for: Thiophene-fused γ-lactams inhibit the SARS-CoV-2 main protease via reversible covalent acylation
Source: Chem Sci. 2024 Apr 16;15(20):7667–78. doi: 10.1039/d4sc01027b (PMC11110133; doi:10.1039/d4sc01027b)
Supplement: SC-015-D4SC01027B-s001 [file SC-015-D4SC01027B-s001.pdf]

Electronic Supplementary Material (ESI) for Chemical Science.
This journal is © The Royal Society of Chemistry 2024

Supporting Information

Thiophene-fused γ-lactams inhibit the SARS-CoV-2 main protease via reversible covalent acylation

Gayatri[1], Lennart Brewitz[1,*], Lewis Ibbotson[1], Eidarus Salah[1], Shyam Basak[1], Hani Choudhry[2], and Christopher J. Schofield[1,*]

[1]*Chemistry Research Laboratory, Department of Chemistry and the Ineos Oxford Institute for Antimicrobial Research, University of Oxford, 12 Mansfield Road, OX1 3TA, Oxford, United Kingdom.*

[2]*Department of Biochemistry, Center for Artificial Intelligence in Precision Medicines, King Abdulaziz University, Jeddah, Saudi Arabia.*

*Email: christopher.schofield@chem.ox.ac.uk or lennart.brewitz@chem.ox.ac.uk

Table of contents

1. General synthesis information S2
2. General synthetic procedures S3-S5
3. Experimental procedures and compound characterizations S6-S36
4. References S37-S38
5. ^{1}H and ^{13}C NMR spectra of thiophene-fused γ-lactam SARS-CoV-2 M^{pro} inhibitors prepared for this study S39-S58

1. General synthesis information

All reagents were from commercial sources (Sigma-Aldrich, Inc.; Fluorochem Ltd; Ambeed, Inc.) and used as received. Anhydrous solvents (Sigma-Aldrich, Inc.) were kept under an atmosphere of nitrogen.

Purifications were performed using a Biotage Isolera One or a Biotage Selekt purification machine (wavelength monitored: 254 and 280 nm) equipped with pre-packed Biotage® SFär flash chromatography cartridges. HPLC grade solvents (Sigma-Aldrich Inc.) were used for purifications, reaction work-ups, and extractions.

Thin layer chromatography (TLC) was carried out using Merck silica gel 60 F254 TLC plates and visualized using UV light or ninhydrin/vanillin stain. Infrared (IR) spectroscopy was performed using a Bruker Tensor-27 Fourier transform infrared (FT-IR) spectrometer. High-resolution mass spectrometry (HRMS) was performed using electrospray ionization (ESI) mass spectrometry (MS) in the positive or negative ionization mode employing a Thermo Scientific Exactive mass spectrometer (ThermoFisher Scientific); data are presented as a mass-to-charge ratio (m/z).

Nuclear magnetic resonance (NMR) spectroscopy was performed using 600, 500, 400, and 300 MHz machines. Chemical shifts for protons are reported in parts per million (ppm) downfield from tetramethylsilane and are referenced to residual protium in the NMR solvent (*i.e.*, DMSO-d_6: δ = 2.50 ppm; $CDCl_3$: δ = 7.26 ppm; acetone-d_6: 2.05 ppm). For ^{13}C NMR, chemical shifts are reported in the scale relative to the NMR solvent (*i.e.*, DMSO-d_6: δ = 39.52 ppm; $CDCl_3$: δ = 77.16 ppm; acetone-d_6: 29.84 ppm). NMR data are reported as follows: chemical shift, multiplicity (br: broad signal), coupling constant (*J*, Hz; to nearest 0.1 Hz), and integration.

2. General synthetic procedures

General Procedure A

A modified version of reported procedures was used.[1, 2] Fe(0) powder (3.4 equiv.) and iron sulfate heptahydrate (0.08 equiv.) were added to a solution of a nitrothiophene (1.0 equiv.) in a 4:1 (v/v) mixture of dioxane and water (0.3 M) at room temperature (rt). The reaction mixture was refluxed for 2-4 h. After cooling to rt, the reaction mixture was filtered through a short pad of Celite®; saturated aqueous $NaHCO_3$ and diethyl ether were added to the resultant filtrate. The layers were separated, and the aqueous phase was extracted with diethyl ether three times; the combined organic extracts were dried using $MgSO_4$, filtered, then concentrated *in vacuo*. The residue was dissolved in anhydrous CH_2Cl_2 (0.2 M); anhydrous triethylamine (2.5 equiv.) and 4-(dimethylamino)pyridine (DMAP; 0.1 equiv.) were added to the resultant solution at rt. The mixture was stirred at rt for 15 min, then cooled to 0 °C, and a sulfonyl chloride (3.0 equiv.) was added dropwise/in portions. The reaction mixture was stirred at rt overnight, then saturated aqueous NH_4Cl was added. The resultant layers were separated, and the aqueous phase was extracted with CH_2Cl_2 three times; the combined organic extracts were dried using $MgSO_4$, filtered, then concentrated *in vacuo*. The crude material was purified using flash column chromatography (20-40% (v/v) EtOAc in cyclohexane) to afford the desired product.

General Procedure B

A modified version of a reported procedure was used.[3] LiOH monohydrate (5.0 equiv.) was added to a solution of a thiophene (1.0 equiv.) in a 2:1:1 (v/v) mixture of THF, water and ethanol (0.1 M) at rt. The reaction mixture was stirred at rt for 1-3 h, then acidified to pH ~ 2 using aqueous HCl (4 M) at 0 °C. EtOAc was added to the mixture, and the layers were separated. The aqueous phase was extracted with EtOAc three times; the combined organic extracts were dried using $MgSO_4$, filtered, then concentrated *in vacuo*. The crude material was purified using flash column chromatography (30-60% (v/v) EtOAc in cyclohexane, EtOAc was supplemented with 0.1% (v/v) formic acid) to afford the desired product.

General Procedure C

A modified version of a reported procedure was used.[4] 1-[Bis(dimethylamino)methylene]-1*H*-1,2,3-triazolo[4,5-*b*]pyridinium-3-oxide hexafluorophosphate (HATU; 1.2 equiv.) and anhydrous *N,N*-diisopropylethylamine (DIPEA; 1.5 equiv.) were added to a solution of a carboxylic acid (1.0 equiv.) in a 1:1 (v/v) mixture of CH_2Cl_2 and CH_3CN (0.05 M) at rt. The reaction mixture was

stirred at rt for 8-16 h, then saturated aqueous NH_4Cl was added. The resultant layers were separated, and the aqueous phase was extracted with CH_2Cl_2 three times; the combined organic extracts were dried using $MgSO_4$, filtered, then concentrated *in vacuo*. The crude material was purified using flash column chromatography (10-60% (v/v) EtOAc in cyclohexane) to afford the desired product.

General Procedure D

Anhydrous cesium carbonate (1.2 equiv.) was added in one portion to a solution of ethyl 2-(2-nitrothiophen-3-yl)acetate (**13**; 1.0 equiv.) in anhydrous DMF (0.1 M) at rt. After stirring at rt for 1 h, an alkyl halide (1.1 equiv.) was slowly added to the mixture, which was then stirred at rt for 8-12 h. Saturated aqueous NH_4Cl and EtOAc were subsequently added to the reaction mixture, the resultant layers were separated, and the aqueous phase was extracted with EtOAc three times; the combined organic extracts were washed with an excess of brine, dried using $MgSO_4$, filtered, then concentrated *in vacuo*. The crude material was purified using flash column chromatography (20-50% (v/v) EtOAc in cyclohexane) to afford the desired product.

General Procedure E

Anhydrous cesium carbonate (2.5 equiv.) was added in one portion to a solution of a γ-lactam (1.0 equiv.) in anhydrous DMF (0.1 M) at rt. After stirring at rt for 1 h, an alkyl halide (2.2-2.5 equiv.) was slowly added to the mixture, which was then stirred at rt for 4-8 h. Saturated aqueous NH_4Cl and EtOAc were subsequently added to the reaction mixture, the resultant layers were separated, and the aqueous phase was extracted with EtOAc three times; the combined organic extracts were washed with an excess of brine, dried using $MgSO_4$, filtered, then concentrated *in vacuo*. The crude material was purified using flash column chromatography (15-40% (v/v) EtOAc in cyclohexane) to afford the desired product.

General Procedure F

A modified version of a reported procedure was used.[5] Grubbs II catalyst (0.05 equiv.) was added to a solution of a diene (1.0 equiv.) in anhydrous CH_2Cl_2 (0.05 M) at rt. The reaction mixture was stirred at rt for 8-12 h, then concentrated *in vacuo*. The crude material was purified using flash column chromatography (10-30% (v/v) EtOAc in cyclohexane) to afford the desired product.

General Procedure G

A modified version of a reported procedure was used.[6] Potassium carbonate (3.0 equiv.) and an

amine (2.0-2.5 equiv.) were added to a solution of 2-(2-bromothiophen-3-yl)acetic acid (**21**; 1.0 equiv.) in pyridine (4.0 M) at rt. Cu(0) powder (0.04 g/mmol) was added to the resultant suspension and the reaction mixture was stirred at 120 °C overnight, then cooled to rt and diluted with water. The mixture was subsequently acidified to pH ~ 2 using aqueous HCl (1 M), and the layers were separated. The aqueous phase was extracted with EtOAc three times; the combined organic extracts were dried using $MgSO_4$, filtered, then concentrated *in vacuo*. The crude material was purified using flash column chromatography (20-60% (v/v) EtOAc in cyclohexane, EtOAc was supplemented with 0.1% (v/v) formic acid) to afford the desired product.

General Procedure H

A modified version of a reported procedure was used.[7] Potassium carbonate (3.0 equiv.) and an amine (1.1-1.2 equiv.) were added to a solution of 2-(2-bromothiophen-3-yl)acetic acid (**21**; 1.0 equiv.) in *tert*-butanol (0.2 M) at rt. Copper(I) iodide (0.1 equiv.) and *N*,*N'*-dimethylethylenediamine (1.0 equiv.) were subsequently added to the resultant suspension. The reaction mixture was stirred at 100 °C overnight, then cooled to rt and concentrated *in vacuo*. The residue was resuspended in EtOAc; the resulting suspension was acidified to pH ~ 2 using aqueous HCl (1 M) and the layers were separated. The aqueous phase was extracted with EtOAc three times; the combined organic extracts were washed with an excess of brine, dried using $MgSO_4$, filtered, then concentrated *in vacuo*. The crude material was purified using flash column chromatography (30-80% (v/v) EtOAc in cyclohexane supplemented with 0.1% (v/v) formic acid using normal phase silica gel, followed by 10-30% (v/v) CH_3CN in water supplemented with 0.1% (v/v) formic acid using reversed-phase silica gel) to afford the desired product.

3. Experimental procedures and compound characterizations

Ethyl 2-(2-nitrothiophen-3-yl)acetate (13)

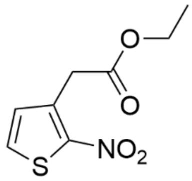

Thiophene **13** was synthesized according to a reported procedure.[1] A solution of 2-nitrothiophene (**12**) (1.00 g, 7.74 mmol, 1.0 equiv.) and ethyl chloroacetate (0.82 mL, 7.74 mmol, 1.0 equiv.) in anhydrous THF (50 mL) was added dropwise to a solution of potassium *tert*-butoxide (2.60 g, 23.2 mmol, 3.0 equiv.) in anhydrous THF (750 mL) at −50 °C. The reaction mixture was stirred at −50 °C for 1 h, then warmed to rt. Acetic acid (4.0 mL) was subsequently added to the reaction mixture, which was then diluted with water (100 mL) and EtOAc (200 mL). The resultant layers were separated, and the aqueous phase was extracted with EtOAc (3 x 50 mL); the combined organic extracts were washed with brine (50 mL), dried using $MgSO_4$, filtered, then concentrated *in vacuo*. The crude material was purified using flash column chromatography (10-30% (v/v) EtOAc in cyclohexane) to afford the desired product **13** as a reddish-brown oil (1.49 g, 89%). The analytical data for **13** are consistent with those reported.[1]

R_f: ~0.35 (4:1 (v/v) cyclohexane-EtOAc); **^{1}H NMR** (500 MHz, 298 K, $CDCl_3$): δ = 7.47 (d, *J* = 5.4 Hz, 1H), 7.02 (d, *J* = 5.4 Hz, 1H), 4.20 (q, *J* = 7.0 Hz, 2H), 4.08 (s, 2H), 1.28 ppm (t, *J* = 7.0 Hz, 3H); **^{13}C NMR** (126 MHz, 298 K, $CDCl_3$): δ = 169.2, 148.5, 136.7, 130.9, 130.3, 61.6, 35.3, 14.3 ppm; **IR** (film): ṽ = 3109, 2985, 1740, 1552, 1501, 1398, 1340, 1257, 1192, 1035 cm^{-1}; **HRMS** (ESI-TOF) calculated for $[C_8H_{10}NO_4S]^+$ $[M+H]^+$: 216.0325, found 216.0326.

Ethyl 2-(2-(*N*-(methylsulfonyl)methylsulfonamido)thiophen-3-yl)acetate (25)

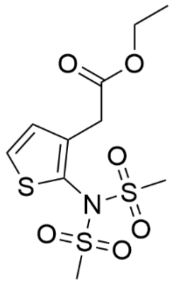

Thiophene **25** was prepared from ethyl 2-(2-nitrothiophen-3-yl)acetate **13** (878 mg, 4.08 mmol, 1.0 equiv.) and methanesulfonyl chloride (0.95 mL, 12.2 mmol, 3.0 equiv.) *via* General Procedure A, and was obtained as a light brown solid (611 mg, 44%).

MP: 122-125 °C; **R_f:** ~0.45 (7:3 (v/v) cyclohexane-EtOAc); **^{1}H NMR** (400 MHz, 298 K, $CDCl_3$):

δ = 7.38 (d, *J* = 5.8 Hz, 1H), 7.12 (d, *J* = 5.8 Hz, 1H), 4.16 (q, *J* = 7.0 Hz, 2H), 3.63 (s, 2H), 3.49 (s, 6H), 1.26 ppm (t, *J* = 7.0 Hz, 3H); **^{13}C NMR** (126 MHz, 298 K, $CDCl_3$): δ = 169.8, 136.8, 129.3, 128.4, 127.2, 61.6, 42.5 (2C), 34.3, 14.3 ppm; **IR** (film): ṽ = 1735, 1419, 1373, 1327, 1218, 1168, 1096, 1032 cm^{-1}; **HRMS** (ESI-TOF) calculated for $[C_{10}H_{16}NO_6S_3]^+$ $[M+H]^+$: 342.0134, found 342.0133.

2-(2-(Methylsulfonamido)thiophen-3-yl)acetic acid (26)

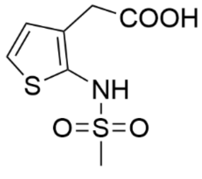

Thiophene **26** was prepared from ethyl 2-(2-(*N*-(methylsulfonyl)methylsulfonamido)thiophen-3-yl)acetate **25** (100 mg, 0.293 mmol) *via* General Procedure B, and was obtained as a yellow solid (63.0 mg, 91%). To our knowledge, analytical data for **26** have not been previously provided.[10]

MP: 75-78 °C; **R$_f$:** ~0.20 (3:2 (v/v) cyclohexane-EtOAc + 0.1% formic acid); **^{1}H NMR** (400 MHz, 298 K, acetone-*d*$_6$): δ = 8.46 (s, 1H), 7.25 (d, *J* = 5.7 Hz, 1H), 7.00 (d, *J* = 5.7 Hz, 1H), 3.72 (s, 2H), 3.04 ppm (s, 3H); **^{13}C NMR** (126 MHz, 298 K, acetone-*d*$_6$): δ = 172.2, 134.8, 131.4, 129.0, 123.0, 39.6, 33.2 ppm; **IR** (film): ṽ = 3570, 3255, 1716, 1562, 1406, 1323, 1207, 1150 cm^{-1}; **HRMS** (ESI-TOF) calculated for $[C_7H_9NO_4S_2Na]^+$ $[M+Na]^+$: 257.9865, found 257.9872.

6-(Methylsulfonyl)-4,6-dihydro-5*H*-thieno[2,3-*b*]pyrrol-5-one (7)

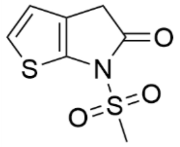

γ-Lactam **7** was prepared from 2-(2-(methylsulfonamido)thiophen-3-yl)acetic acid **26** (1.01 g, 4.29 mmol) *via* General Procedure C, and was obtained as a yellow solid (609 mg, 65%). To our knowledge, analytical data for **7** have not been previously provided.[10]

MP: 160-162 °C; **R$_f$:** ~0.60 (2:3 (v/v) cyclohexane-EtOAc); **^{1}H NMR** (500 MHz, 298 K, $CDCl_3$): δ = 6.96 (d, *J* = 5.3 Hz, 1H), 6.85 (d, *J* = 5.3 Hz, 1H), 3.69 (s, 2H), 3.40 ppm (s, 3H); **^{13}C NMR** (126 MHz, 298 K, $CDCl_3$): δ = 175.2, 138.8, 121.2, 120.5, 120.3, 41.1, 37.1 ppm; **IR** (film): ṽ = 1750, 1388, 1356, 1170, 1086 cm^{-1}; **HRMS** (ESI-TOF) calculated for $[C_7H_8NO_3S_2]^+$ $[M+H]^+$: 217.9940, found 217.9944.

Di-*tert*-butyl 2-(3-nitrothiophen-2-yl)malonate (27)

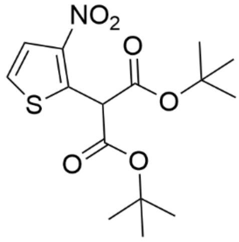

Thiophene **27** was synthesized according to a reported procedure.[13] Sodium hydride (60% dispersion in mineral oil, 245 mg, 6.11 mmol, 2.0 equiv.) was added to a solution of di-*tert*-butyl malonate (0.75 mL, 3.36 mmol, 1.1 equiv.) in anhydrous DMSO (15 mL) at rt. After stirring at 100 °C for 1 h, the mixture was cooled to rt and 2-chloro-3-nitrothiophene (500 mg, 3.06 mmol, 1.0 equiv.) was added in one portion. The red-colored reaction mixture was stirred at 60 °C for 2 h, then cooled to rt and acidified using aqueous HCl (0.1 M) until the solution turned yellow. EtOAc (30 mL) and water (20 mL) were subsequently added to the resultant mixture and the layers were separated. The aqueous phase was extracted with EtOAc (3 x 20 mL); the combined organic extracts were dried using $MgSO_4$, filtered, then concentrated *in vacuo*. The crude material was purified using flash column chromatography (10-30% (v/v) EtOAc in cyclohexane) to afford **27** as a yellow oil (361 mg, 34%). To our knowledge, analytical data for **27** have not been previously provided.[13]

R_f: ~0.40 (4:1 (v/v) cyclohexane-EtOAc); **^{1}H NMR** (500 MHz, 298 K, $CDCl_3$): δ = 7.63 (d, *J* = 5.7 Hz, 1H), 7.27 (d, *J* = 5.7 Hz, 1H), 5.68 (s, 1H), 1.50 ppm (s, 18H); **^{13}C NMR** (126 MHz, 298 K, $CDCl_3$): δ = 165.4 (2C), 145.7, 138.0, 124.8, 124.1, 83.8 (2C), 54.2, 28.0 ppm (6C); **IR** (film): ṽ = 1737, 1542, 1508, 1457, 1384, 1370, 1327, 1259, 1228, 1158, 1138 cm^{-1}; **HRMS** (ESI-TOF) calculated for $[C_{15}H_{21}NO_6SNa]^+$ $[M+Na]^+$: 366.0982, found 366.0990.

Di-*tert*-butyl 2-(3-(*N*-(methylsulfonyl)methylsulfonamido)thiophen-2-yl)malonate (28a) and *tert*-butyl 2-(3-(*N*-(methylsulfonyl)methylsulfonamido)thiophen-2-yl)acetate (28b)

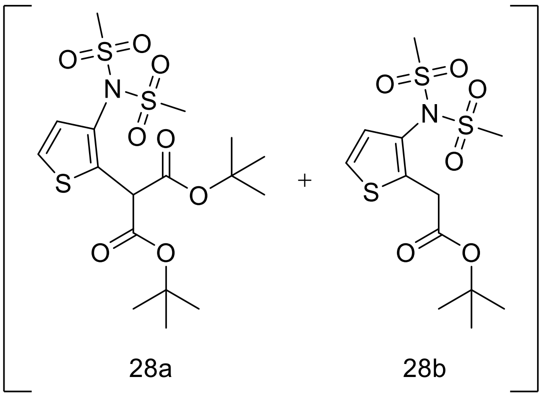

Thiophenes **28a** and **28b** were prepared from di-*tert*-butyl 2-(3-nitrothiophen-2-yl)malonate **27**

(1.50 g, 4.37 mmol, 1.0 equiv.) and methanesulfonyl chloride (1.0 mL, 13.1 mmol, 3.0 equiv.) *via* General Procedure A, and were obtained as an inseparable amorphous mixture (820 mg, ~6:1 (w/w) of **28a** (33%) and **28b** (8%)), which was used in the following step without further purification.

28a:

R_f: ~0.35 (4:1 (v/v) cyclohexane-EtOAc); **^{1}H NMR** (400 MHz, 298 K, $CDCl_3$): δ = 7.40 (dd, *J* = 5.4, 1.0 Hz, 1H), 6.98 (d, *J* = 5.4 Hz, 1H), 4.90 (d, *J* = 1.0 Hz, 1H), 3.44 (s, 6H), 1.47 ppm (s, 18H); **^{13}C NMR** (126 MHz, 298 K, $CDCl_3$): δ = 165.4 (2C), 136.8, 128.4, 126.2, 126.0, 83.4 (2C), 53.8, 42.5 (2C), 27.9 ppm (6C); **HRMS** (ESI-TOF) calculated for $[C_{17}H_{27}NO_8S_3Na]^+$ $[M+Na]^+$: 492.0791, found 492.0791.

28b:

R_f: ~0.35 (4:1 (v/v) cyclohexane-EtOAc); **^{1}H NMR** (400 MHz, 298 K, $CDCl_3$): δ = 7.29 (d, *J* = 5.4 Hz, 1H), 6.96 (d, *J* = 5.4 Hz, 1H), 3.79 (s, 2H), 3.42 (s, 6H), 1.46 ppm (s, 9H); **^{13}C NMR** (126 MHz, 298 K, $CDCl_3$): δ = 168.5, 138.2, 127.7, 126.8, 124.3, 82.5, 42.7 (2C), 35.0, 28.1 ppm (3C); **HRMS** (ESI-TOF) calculated for $[C_{12}H_{19}NO_6S_3Na]^+$ $[M+Na]^+$: 392.0267, found 392.0267.

2-(3-(*N*-(Methylsulfonyl)methylsulfonamido)thiophen-2-yl)acetic acid (29)

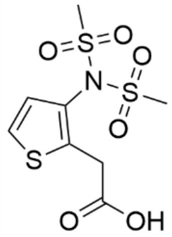

Thiophene **29** was synthesized according to a modified version of a reported procedure.[13] The mixture of thiophenes **28a** and **28b** (814 mg) was dissolved in HCl (4 M in dioxane, 4.4 mL) at rt. The reaction mixture was stirred at 70 °C for 14 h, then cooled to rt and diluted with water (30 mL) and EtOAc (20 mL). The resultant layers were separated, and the aqueous phase was extracted with EtOAc (3 x 20 mL); the combined organic extracts were dried using $MgSO_4$, filtered, then concentrated *in vacuo*. The crude material was purified using flash column chromatography (40-60% (v/v) EtOAc in cyclohexane, EtOAc was supplemented with 0.1% (v/v) formic acid) to afford **29** as an off-white solid (504 mg, 89%).

MP: 128-130 °C; R_f**:** ~0.30 (1:1 (v/v) cyclohexane-EtOAc + 0.1% formic acid); **^{1}H NMR** (500 MHz, 298 K, DMSO-*d*$_6$): δ = 7.54 (d, *J* = 5.4 Hz, 1H), 7.13 (d, *J* = 5.4 Hz, 1H), 3.83 (s, 2H), 3.48 ppm (s, 6H); **^{13}C NMR** (126 MHz, 298 K, DMSO-*d*$_6$): δ = 170.6, 137.9, 127.7, 127.3, 124.3, 42.6 (2C), 32.9 ppm; **IR** (film): ṽ = 3160, 1721, 1361, 1215, 1166, 1041 cm^{-1}; **HRMS** (ESI-TOF) calculated for $[C_8H_{10}NO_6S_3]^-$ $[M-H]^-$: 311.9676, found 311.9683.

2-(3-(Methylsulfonamido)thiophen-2-yl)acetic acid (30)

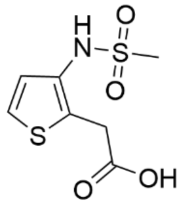

Thiophene **30** was prepared from 2-(3-(*N*-(methylsulfonyl)methylsulfonamido)thiophen-2-yl)acetic acid **29** (734 mg, 2.34 mmol) *via* General Procedure B, and was obtained as a pale white amorphous solid (515 mg, 93%).

R_f: ~0.35 (3:7 (v/v) cyclohexane-EtOAc = 0.1% formic acid); **^{1}H NMR** (400 MHz, 298 K, acetone-*d*$_6$): δ = 8.16 (s, 1H), 7.37 (d, *J* = 5.4 Hz, 1H), 7.11 (d, *J* = 5.4 Hz, 1H), 3.97 (s, 2H), 2.96 ppm (s, 3H); **^{13}C NMR** (126 MHz, 298 K, acetone-*d*$_6$): δ = 171.8, 133.7, 127.7, 126.1, 124.3, 39.7, 32.5 ppm; **IR** (film): ṽ = 3571, 3255, 1720, 1560, 1381, 1317, 1197, 1154, 1086, 1027 cm^{-1}; **HRMS** (ESI-TOF) calculated for $[C_7H_8NO_4S_2]^-$ [M-H]$^-$: 233.9900, found 233.9907.

4-(Methylsulfonyl)-4,6-dihydro-5*H*-thieno[3,2-*b*]pyrrol-5-one (8)

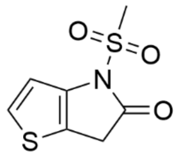

γ-Lactam **8** was prepared from 2-(3-(methylsulfonamido)thiophen-2-yl)acetic acid **30** (510 mg, 2.17 mmol) *via* General Procedure C, and was obtained as a light pink solid (364 mg, 77%).

MP: 165-167 °C; **R_f:** ~0.65 (2:3 (v/v) cyclohexane-EtOAc); **^{1}H NMR** (500 MHz, 298 K, $CDCl_3$): δ = 7.29 (dt, *J* = 5.3, 1.0 Hz, 1H), 7.23 (d, *J* = 5.3 Hz, 1H), 3.78 (d, *J* = 1.0 Hz, 2H), 3.38 ppm (s, 3H); **^{13}C NMR** (126 MHz, 298 K, $CDCl_3$): δ = 175.7, 140.7, 127.2, 115.7, 115.4, 41.4, 36.7 ppm; **IR** (film): ṽ = 1745, 1451, 1349, 1288, 1257, 1193, 1162, 1086, 1057 cm^{-1}; **HRMS** (ESI-TOF) calculated for $[C_7H_8NO_3S_2]^+$ [M+H]$^+$: 217.9940, found 217.9947.

4-(2-Ethoxy-2-oxoethyl)thiophene-3-carboxylic acid (31)

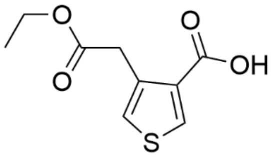

Thiophene **31** was synthesized according to a modified version of a reported procedure.[14] Anhydrous ethanol (15 mL) was added dropwise to sodium (300 mg, 13.0 mmol, 2.4 equiv.) at 0 °C. After stirring at 0 °C for 1 h, all of sodium was dissolved and ethyl acetoacetate (1.0 mL,

8.14 mmol, 1.5 equiv.) was added to the resultant solution. The mixture was then stirred at 0 °C for 5 min, and 4-bromo-3-thiophenecarboxylic acid (1.12 g, 5.43 mmol, 1.0 equiv.) and copper(I) bromide (156 mg, 1.09 mmol, 0.2 equiv.) were subsequently added at 0 °C. The reaction mixture was refluxed overnight, then diluted with water (50 mL) at rt. The resultant suspension was filtered, and the filtrate was acidified to pH ~ 3 using aqueous HCl (6 M). The solid residue was collected *via* vacuum filtration, washed with water (2 x 10 mL), and dried to give **31** as a white solid (902 mg, 78%). The analytical data for **31** are consistent with those reported.[15]

MP: 158-160 °C; **R$_f$:** ~0.35 (3:2 (v/v) cyclohexane-EtOAc + 0.1% formic acid); **^{1}H NMR** (400 MHz, 298 K, acetone-*d*$_6$): δ = 8.26 (d, *J* = 3.4 Hz, 1H), 7.37 (dt, *J* = 3.4, 1.0 Hz, 1H), 4.09 (q, *J* = 7.0 Hz, 2H), 3.92 (d, *J* = 1.0 Hz, 2H), 1.19 ppm (t, *J* = 7.0 Hz, 3H); **^{13}C NMR** (126 MHz, 298 K, acetone-*d*$_6$): δ = 171.4, 164.2, 136.7, 135.2, 133.0, 126.4, 60.8, 35.7, 14.5 ppm; **IR** (film): ṽ = 3492, 1726, 1674, 1467, 1299, 1192, 1160, 1033 cm^{-1}; **HRMS** (ESI-TOF) calculated for $[C_9H_9O_4S]^-$ [M-H]$^-$: 213.0227, found 213.0235.

Ethyl 2-(4-((*tert*-butoxycarbonyl)amino)thiophen-3-yl)acetate (32)

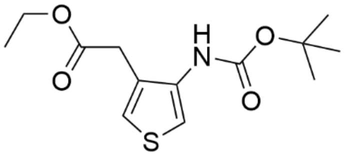

Thiophene **32** was synthesized according to a modified version of a reported procedure.[16] Anhydrous triethylamine (0.46 mL, 3.27 mmol, 1.5 equiv.) and ethyl chloroformate (0.31 mL, 3.27 mmol, 1.5 equiv.) were added dropwise to a solution of 4-(2-ethoxy-2-oxoethyl)thiophene-3-carboxylic acid **31** (467 mg, 2.18 mmol, 1.0 equiv.) in anhydrous acetone (20 mL) at 0 °C. After stirring at 0 °C for 1 h, a solution of sodium azide (213 mg, 3.27 mmol, 1.5 equiv.) in water (0.74 mL) was added dropwise to the mixture at 0 °C. The reaction mixture was stirred at rt for 1 h, then diluted with water (30 mL) and EtOAc (25 mL). The resultant layers were separated, and the aqueous phase was extracted with EtOAc (3 x 20 mL); the combined organic extracts were dried using $MgSO_4$, filtered, then concentrated *in vacuo*. The residue was redissolved in a 1:1 (v/v) mixture of *tert*-butanol and toluene (58 mL) and the mixture was stirred at 80 °C overnight, then concentrated *in vacuo*. The crude material was purified using flash column chromatography (10-20% (v/v) EtOAc in cyclohexane) to afford **32** as a yellow oil (374 mg, 60%). The analytical data for **32** are consistent with those reported.[13]

R$_f$: ~0.50 (7:3 (v/v) cyclohexane-EtOAc); **^{1}H NMR** (500 MHz, 298 K, DMSO-*d*$_6$): δ = 8.74 (s, 1H), 7.29 (s, 1H), 7.23 (d, *J* = 1.0 Hz, 1H), 4.07 (q, *J* = 7.0 Hz, 2H), 3.66 (br s, 2H), 1.46 (s, 9H), 1.19 ppm (t, *J* = 7.0 Hz, 3H); **^{13}C NMR** (126 MHz, 298 K, DMSO-*d*$_6$): δ = 170.6, 153.3, 135.6,

128.2, 122.7, 111.2, 79.0, 60.2, 33.0, 28.1 (3C), 14.0 ppm; **IR** (film): ṽ = 3377, 1729, 1537, 1369, 1319, 1249, 1164, 1046, 1021 cm^{-1}; **HRMS** (ESI-TOF) calculated for $[C_{13}H_{20}NO_4S]^+$ $[M+H]^+$: 286.1108.

Ethyl 2-(4-aminothiophen-3-yl)acetate hydrochloride (33)

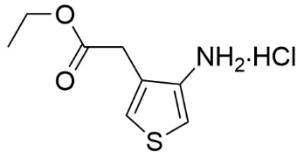

Thiophene **33** was synthesized according to a reported procedure.[13] HCl (4 M in dioxane, 3.7 mL) was added dropwise to a solution of ethyl 2-(4-((*tert*-butoxycarbonyl)amino)thiophen-3-yl)acetate **32** (600 mg, 2.10 mmol) in anhydrous dioxane (5.5 mL) at rt. After stirring at rt for 24 h, diethyl ether (23 mL) was added to the reaction mixture, which was then stirred at rt for 1 h. The resultant solid residue was subsequently collected *via* vacuum filtration, washed with diethyl ether (2 x 10 mL), and dried to afford the desired product **33** as a white amorphous solid (223 mg, 48%). The analytical data for **33** were consistent with those reported.[13]

R_f: ~0.10 (3/7 (v/v) methanol in CH_2Cl_2); **^{1}H NMR** (400 MHz, 298 K, DMSO-d_6): δ = 10.05 (s, 2H), 7.48 (dt, J = 3.5, 1.0 Hz, 1H), 7.45 (t, J = 3.5 Hz, 1H), 4.12 (q, J = 7.0 Hz, 2H), 3.76 (d, J = 1.0 Hz, 2H), 1.22 ppm (t, J = 7.0 Hz, 3H); **^{13}C NMR** (126 MHz, 298 K, DMSO-d_6): δ = 170.1, 128.6, 126.0, 119.1, 60.7, 32.4, 14.1 ppm; **IR** (film): ṽ = 2932, 1725, 1374, 1267, 1164, 1045, 1026 cm^{-1}; **HRMS** (ESI-TOF) calculated for $[C_8H_{12}NO_2S]^+$ $[M-Cl]^+$: 186.0583, found 186.0589.

2-(4-(Methylsulfonamido)thiophen-3-yl)acetic acid (34)

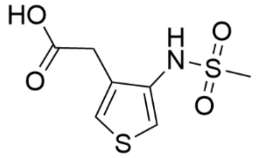

Thiophene **34** was synthesized according to a modified version of the reported procedures.[2, 3] Anhydrous triethylamine (0.95 mL, 6.82 mmol, 3.5 equiv.) and 4-(dimethylamino)pyridine (23.8 mg, 0.195 mmol, 0.1 equiv.) were added to a solution of ethyl 2-(4-aminothiophen-3-yl)acetate hydrochloride **33** (432 mg, 1.95 mmol, 1.0 equiv.) in anhydrous CH_2Cl_2 (15 mL) at rt. After stirring at rt for 15 min, methanesulfonyl chloride (0.45 mL, 5.84 mmol, 3.0 equiv.) was added dropwise to the mixture at 0 °C. The reaction mixture was stirred at rt overnight, then saturated aqueous NH_4Cl (10 mL) was added. The resultant layers were separated, and the aqueous phase was extracted with CH_2Cl_2 (3 x 10 mL); the combined organic extracts were dried using $MgSO_4$, filtered, then concentrated *in vacuo*. The residue was redissolved in a 2:1:1 (v/v) mixture of THF, water and

ethanol (15 mL), and LiOH monohydrate (409 mg, 9.75 mmol, 5.0 equiv.) was added to the resultant solution at rt. The reaction mixture was stirred at rt for 2 h, then acidified to pH ~ 2 using aqueous HCl (4 M) at 0 °C. EtOAc (20 mL) was added, and the layers were separated. The aqueous phase was extracted with EtOAc (3 x 15 mL); the combined organic extracts were dried using $MgSO_4$, filtered, then concentrated *in vacuo*. The crude material was purified using flash column chromatography (30-45% (v/v) EtOAc in cyclohexane, EtOAc was supplemented with 0.1% (v/v) formic acid) to afford **34** as a yellow solid (317 mg, 69%).

MP: 133-135 °C; **R_f:** ~0.20 (1:1 (v/v) cyclohexane-EtOAc + 0.1% formic acid); **^{1}H NMR** (400 MHz, 298 K, acetone-*d_6*): δ = 7.38 (dt, *J* = 3.4, 1.0 Hz, 1H), 7.33 (d, *J* = 3.4 Hz, 1H), 3.77 (d, *J* = 1.0 Hz, 2H), 2.99 ppm (s, 3H); **^{13}C NMR** (126 MHz, 298 K, acetone-*d_6*): δ = 172.8, 135.7, 131.1, 124.5, 116.2, 39.2, 33.8 ppm; **IR** (film): ṽ = 3578, 3263, 1717, 1464, 1412, 1364, 1317, 1201, 1154 cm^{-1}; **HRMS** (ESI-TOF) calculated for $[C_7H_8NO_4S_2]^-$ $[M-H]^-$: 233.9900, found 233.9906.

1-(Methylsulfonyl)-1*H*-thieno[3,4-*b*]pyrrol-2(3*H*)-one (9)

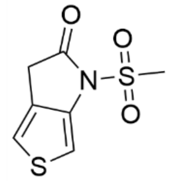

γ-Lactam **9** was prepared from 2-(4-(methylsulfonamido)thiophen-3-yl)acetic acid **34** (314 mg, 1.33 mmol) *via* General Procedure C, and was obtained as a yellow solid (262 mg, 90%).

MP: 172-174 °C; **R_f:** ~0.50 (1:1 (v/v) cyclohexane-EtOAc); **^{1}H NMR** (500 MHz, 298 K, $CDCl_3$): δ = 7.08 (dt, *J* = 2.5, 1.5 Hz, 1H), 7.01 (d, *J* = 2.5 Hz, 1H), 3.62 (d, *J* = 1.5 Hz, 2H), 3.39 ppm (s, 3H); **^{13}C NMR** (126 MHz, 298 K, $CDCl_3$): δ = 175.4, 137.0, 125.0, 118.5, 103.1, 41.3, 34.8 ppm; **IR** (film): ṽ = 1742, 1504, 1396, 1350, 1223, 1180, 1165, 1133, 1101 cm^{-1}; **HRMS** (ESI-TOF) calculated for $[C_7H_7NO_3S_2Na]^+$ $[M+Na]^+$: 239.9760, found 239.9767.

1*H*-Thieno[3,4-*d*]imidazol-2(3*H*)-one (35)

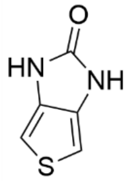

Thiophene **35** was synthesized according to a reported procedure.[17] Urea (379 mg, 6.31 mmol, 1.2 equiv.) was added to a solution of thiophene-3,4-diamine (600 mg, 5.26 mmol, 1.0 equiv.) in 1-pentanol (26 mL) at rt. The reaction mixture was refluxed for 5 h, then concentrated *in vacuo*. The crude material was purified using flash column chromatography (80-100% (v/v) EtOAc in

cyclohexane using normal phase silica gel, followed by 5-15% (v/v) CH_3CN in water supplemented with 0.1% (v/v) formic acid using reversed-phase silica gel) to afford **35** as a yellow solid (245 mg, 33%). The analytical data for **35** are consistent with those reported.[17]

MP: 148-152 °C; **R_f:** ~0.20 (2:3 (v/v) cyclohexane-EtOAc); **^{1}H NMR** (400 MHz, 298 K, DMSO-*d$_6$*): δ = 10.30 (s, 2H), 6.43 ppm (s, 2H); **^{13}C NMR** (126 MHz, 298 K, DMSO-*d$_6$*): δ = 160.3, 132.1 (2C), 93.1 ppm (2C); **IR** (film): ṽ = 3139, 3014, 1712, 1560, 1439, 1348, 1156, 1006 cm^{-1}; **HRMS** (ESI-TOF) calculated for $[C_5H_5N_2OS]^+$ $[M+H]^+$: 141.0117, found 141.0126.

1-(Methylsulfonyl)-1*H*-thieno[3,4-*d*]imidazol-2(3*H*)-one and 1,3-bis(methylsulfonyl)-1*H*-thieno[3,4-*d*]imidazol-2(3*H*)-one (36 and 10)

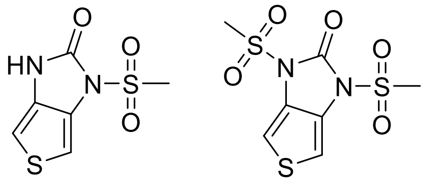

36 **10**

Thiophenes **36** and **10** were synthesized according to a modified version of a reported procedure.[18] Sodium hydride (60% dispersion in mineral oil, 25.7 mg, 0.642 mmol, 1.5 equiv.) was added to a solution of 1*H*-thieno[3,4-*d*]imidazol-2(3*H*)-one **35** (60.0 mg, 0.428 mmol, 1.0 equiv.) in anhydrous DMF (1.0 mL) at rt. After stirring at rt for 40 min, methanesulfonyl chloride (0.05 mL, 0.642 mmol, 1.5 equiv.) was added dropwise to the resultant mixture. The reaction mixture was stirred at rt for 16 h, then saturated aqueous NH_4Cl (10 mL) and EtOAc (5.0 mL) were added. The resultant layers were separated, and the aqueous phase was extracted with EtOAc (3 x 5.0 mL); the combined organic extracts were washed with an excess of brine, dried using $MgSO_4$, filtered, then concentrated *in vacuo*. The crude material was purified using flash column chromatography (30-60% (v/v) EtOAc in cyclohexane using normal phase silica gel, followed by 10-50% (v/v) CH_3CN in water supplemented with 0.1% (v/v) formic acid using reversed-phase silica gel) to afford **36** (11.0 mg, 12%) and **10** (32.0 mg, 25%) as white solids.

36:

MP: 174-176 °C; **R_f:** ~0.35 (2:3 (v/v) cyclohexane-EtOAc); **^{1}H NMR** (500 MHz, 298 K, DMSO-*d$_6$*): δ = 11.40 (s, 1H), 6.96 (d, *J* = 2.5 Hz, 1H), 6.77 (d, *J* = 2.5 Hz, 1H), 3.53 ppm (s, 3H); **^{13}C NMR** (126 MHz, 298 K, DMSO-*d$_6$*): δ = 154.6, 128.8, 126.8, 100.9, 96.4, 40.7 ppm; **IR** (film): ṽ = 3180, 1731, 1435, 1363, 1304, 1175, 1155, 1049 cm^{-1}; **HRMS** (ESI-TOF) calculated for $[C_6H_7N_2O_3S_2]^+$ $[M+H]^+$: 218.9893, found 218.9898.

10:

MP: 181-184 °C; **R_f:** ~0.50 (2:3 (v/v) cyclohexane-EtOAc); **^{1}H NMR** (400 MHz, 298 K, DMSO-d_6): δ = 7.16 (s, 2H), 3.63 ppm (s, 6H); **^{13}C NMR** (126 MHz, 298 K, DMSO-d_6): δ = 149.9, 124.5 (2C), 102.5 (2C), 40.9 ppm (2C); **IR** (film): ṽ = 1744, 1375, 1358, 1272, 1183, 1171, 1126, 1021 cm^{-1}; **HRMS** (ESI-TOF) calculated for $[C_7H_9N_2O_5S_3]^+$ $[M+H]^+$: 296.9668, found 296.9660.

1-(Methylsulfonyl)indolin-2-one (11)

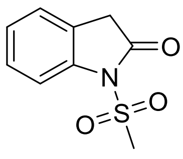

To a solution of 2-(2-(methylsulfonamido)phenyl)acetic acid (1.0 g, 4.45 mmol, 1.0 equiv.) in CH_2Cl_2 (50 mL) were added sequentially DIPEA (1.55 mL, 8.90 mmol, 2.0 equiv.) and HATU (2.0 g, 5.3 mmol, 1.2 equiv.); the reaction mixture was then stirred at rt for 16 h, before being diluted with saturated aqueous NH_4Cl (50 mL), water (50 mL), and CH_2Cl_2 (100 mL). The resultant layers were separated, and the aqueous phase was extracted with CH_2Cl_2 (100 mL). The combined organic extracts were washed with brine (200 mL), dried using Na_2SO_4, filtered; then concentrated *in vacuo*. The crude material was purified using flash column chromatography (0-50% (v/v) EtOAc in cyclohexane) to afford **11** as a pale pink solid (586 mg, 60%). The analytical data for **11** are consistent with those reported.[26]
R_f: ~0.30 (3:10 (v/v) cyclohexane-EtOAc); **^{1}H NMR** (300 MHz, 298 K, $CDCl_3$): δ = 7.76 (d, *J* = 8.2 Hz, 1H), 7.34 – 7.26 (m, 2H), 7.17 (t, *J* = 7.2 Hz, 1H), 3.71 (s, 2H), 3.42 ppm (s, 3H); **^{13}C NMR** (100 MHz, 298 K, $CDCl_3$): δ = 174.0, 140.4, 128.7, 125.0, 124.9, 123.1, 113.9, 41.8, 36.3 ppm; **IR** (film): ṽ = 3014, 2930, 1743, 1613, 1476, 1464, 1356, 1332, 1301, 1235, 1200, 1170, 1146, 1087, 1031 cm^{-1}; **HRMS** (ESI-TOF) calculated for $[C_9H_9NO_3S]^+$ $[M+H]^+$: 211.0303, found 211.0306.

***rac*-Ethyl 2-(2-nitrothiophen-3-yl)propanoate (14a)**

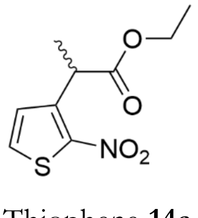

Thiophene **14a** was prepared from ethyl 2-(2-nitrothiophen-3-yl)acetate **13** (435 mg, 2.02 mmol, 1.0 equiv.) and iodomethane (0.14 mL, 2.22 mmol, 1.1 equiv.) *via* General Procedure D, and was obtained as a pale white solid (354 mg, 76%).
MP: 69-71 °C; **R_f:** ~0.40 (4:1 (v/v) cyclohexane-EtOAc); **^{1}H NMR** (500 MHz, 298 K, $CDCl_3$): δ = 7.46 (d, *J* = 5.5 Hz, 1H), 7.08 (d, *J* = 5.5 Hz, 1H), 4.69 (q, *J* = 7.5 Hz, 1H), 4.16 (q, *J* = 7.0 Hz,

2H), 1.56 (d, *J* = 7.5 Hz, 3H), 1.23 ppm (t, *J* = 7.0 Hz, 3H); **^{13}C NMR** (126 MHz, 298 K, $CDCl_3$): δ = 172.7, 147.6, 143.0, 130.6, 128.6, 61.5, 39.5, 17.6, 14.2 ppm; **IR** (film): ṽ = 1736, 1542, 1501, 1393, 1338, 1190, 1072, 1024 cm^{-1}; **HRMS** (ESI-TOF) calculated for $[C_9H_{12}NO_4S]^+$ $[M+H]^+$: 230.0482, found 230.0481.

***rac*-Ethyl 2-(2-nitrothiophen-3-yl)pentanoate (14b)**

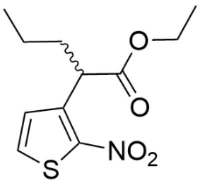

Thiophene **14b** was prepared from ethyl 2-(2-nitrothiophen-3-yl)acetate **13** (1.00 g, 4.65 mmol, 1.0 equiv.) and iodopropane (0.50 mL, 5.12 mmol, 1.1 equiv.) *via* General Procedure D, and was obtained as a yellow amorphous solid (620 mg, 52%).

R_f: ~0.55 (4:1 (v/v) cyclohexane-EtOAc); **^{1}H NMR** (400 MHz, 298 K, $CDCl_3$): δ = 7.44 (d, *J* = 5.7 Hz, 1H), 7.15 (d, *J* = 5.7 Hz, 1H), 4.75 (t, *J* = 7.5 Hz, 1H), 4.22 – 4.12 (m, 2H), 2.07 (dddd, *J* = 13.0, 9.5, 7.5, 6.0 Hz, 1H), 1.79 (dddd, *J* = 13.5, 9.5, 7.5, 6.0 Hz, 1H), 1.49 – 1.26 (m, 2H), 1.24 (t, *J* = 7.0 Hz, 3H), 0.93 ppm (t, *J* = 7.5 Hz, 3H); **^{13}C NMR** (126 MHz, 298 K, $CDCl_3$): δ = 172.4, 147.9, 141.9, 130.4, 128.9, 61.4, 44.4, 35.2, 20.9, 14.3, 13.9 ppm; **IR** (film): ṽ = 1736, 1542, 1504, 1466, 1400, 1337, 1237, 1185, 1103, 1032 cm^{-1}; **HRMS** (ESI-TOF) calculated for $[C_{11}H_{16}NO_4S]^+$ $[M+H]^+$: 258.0795, found 258.0798.

***rac*-Ethyl 3-methyl-2-(2-nitrothiophen-3-yl)butanoate (14c)**

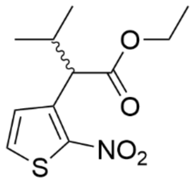

Thiophene **14c** was prepared from ethyl 2-(2-nitrothiophen-3-yl)acetate **13** (450 mg, 2.09 mmol, 1.0 equiv.) and 2-iodopropane (0.23 mL, 2.30 mmol, 1.1 equiv.) *via* General Procedure D, and was obtained as a yellow amorphous solid (418 mg, 78%).

R_f: ~0.55 (4:1 (v/v) cyclohexane-EtOAc); **^{1}H NMR** (500 MHz, 298 K, $CDCl_3$): δ = 7.44 (d, *J* = 5.6 Hz, 1H), 7.29 (d, *J* = 5.6 Hz, 1H), 4.66 (d, *J* = 9.5 Hz, 1H), 4.21 – 4.05 (m, 2H), 2.31 (dhept, *J* = 9.5, 6.5 Hz, 1H), 1.22 (t, *J* = 7.0 Hz, 3H), 1.04 (d, *J* = 6.5 Hz, 3H), 0.80 ppm (d, *J* = 7.0 Hz, 3H); **^{13}C NMR** (126 MHz, 298 K, $CDCl_3$): δ = 172.1, 148.5, 140.8, 130.2, 129.2, 61.2, 50.9, 33.1, 20.9, 20.0, 14.2 ppm; **IR** (film): ṽ = 1733, 1540, 1504, 1402, 1332, 1244, 1187, 1122, 1032 cm^{-1}; **HRMS** (ESI-TOF) calculated for $[C_{11}H_{16}NO_4S]^+$ $[M+H]^+$: 258.0795, found 258.0802.

***rac*-Ethyl 2-(2-nitrothiophen-3-yl)-3-phenylpropanoate (14d)**

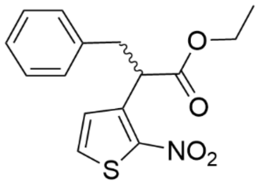

Thiophene **14d** was prepared from ethyl 2-(2-nitrothiophen-3-yl)acetate **13** (1.00 g, 4.65 mmol, 1.0 equiv.) and benzyl bromide (0.61 mL, 5.12 mmol, 1.1 equiv.) *via* General Procedure D, and was obtained as a yellow amorphous solid (1.33 g, 94%).

R_f: ~0.50 (9:1 (v/v) cyclohexane-EtOAc); **^{1}H NMR** (400 MHz, 298 K, $CDCl_3$): δ = 7.42 (d, *J* = 5.5 Hz, 1H), 7.25 – 7.22 (m, 2H), 7.21 – 7.18 (m, 1H), 7.17 – 7.12 (m, 3H), 5.03 (t, *J* = 7.5 Hz, 1H), 4.11 (qd, *J* = 7.0, 2.0 Hz, 2H), 3.41 – 3.33 (m, 1H), 3.14 – 3.06 (m, 1H), 1.14 ppm (t, *J* = 7.0 Hz, 3H); **^{13}C NMR** (126 MHz, 298 K, $CDCl_3$): δ = 171.7, 148.0, 140.9, 137.8, 130.4, 129.1, 129.1 (2C), 128.6 (2C), 126.9, 61.5, 46.6, 39.1, 14.2 ppm; **IR** (film): ṽ = 1736, 1541, 1503, 1400, 1336, 1199, 1164, 1033 cm^{-1}; **HRMS** (ESI-TOF) calculated for $[C_{15}H_{15}NO_4SNa]^+$ $[M+Na]^+$: 328.0614, found 328.0614.

***rac*-Ethyl 4-cyano-2-(2-nitrothiophen-3-yl)butanoate (14e)**

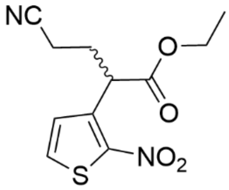

Thiophene **14e** was synthesized according to a modified version of a reported procedure.[11] 1,1,3,3-Tetramethylguanidine (0.12 mL, 0.930 mmol, 0.2 equiv.) was added dropwise to a solution of ethyl 2-(2-nitrothiophen-3-yl)acetate **13** (1.00 g, 4.65 mmol, 1.0 equiv.) in anhydrous THF (23 mL) at rt. After stirring at rt for 1 h, acrylonitrile (0.46 mL, 6.98 mmol, 1.5 equiv.) was added dropwise to the solution. The reaction mixture was stirred at rt overnight, then saturated aqueous NH_4Cl (15 mL) and EtOAc (30 mL) were added. The resultant layers were separated, and the aqueous phase was extracted with EtOAc (3 x 15 mL); the combined organic extracts were dried using $MgSO_4$, filtered, then concentrated *in vacuo*. The crude material was purified using flash column chromatography (30-50% (v/v) EtOAc in cyclohexane) to afford **14e** as a yellow amorphous solid (687 mg, 55%).

R_f: ~0.30 (7:3 (v/v) cyclohexane-EtOAc); **^{1}H NMR** (400 MHz, 298 K, $CDCl_3$): δ = 7.52 (d, *J* = 5.5 Hz, 1H), 7.07 (d, *J* = 5.5 Hz, 1H), 4.73 (t, *J* = 7.0 Hz, 1H), 4.19 (q, *J* = 7.0 Hz, 2H), 2.57 – 2.39 (m, 3H), 2.23 – 2.12 (m, 1H), 1.23 ppm (t, *J* = 7.0 Hz, 3H); **^{13}C NMR** (126 MHz, 298 K, $CDCl_3$):

δ = 170.7, 148.5, 139.2, 131.2, 128.8, 118.5, 62.1, 43.7, 28.1, 15.7, 14.2 ppm; **IR** (film): ṽ = 3111, 2248, 1735, 1542, 1503, 1401, 1338, 1234, 1196, 1023 cm^{-1}; **HRMS** (ESI-TOF) calculated for $[C_{11}H_{12}N_2O_4SNa]^+$ $[M+Na]^+$: 291.0410, found 291.0419.

***rac*-Ethyl 2-(2-nitrothiophen-3-yl)pent-4-enoate (14f)**

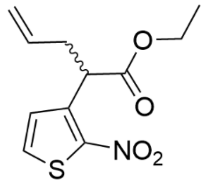

Thiophene **14f** was prepared from ethyl 2-(2-nitrothiophen-3-yl)acetate **13** (450 mg, 2.09 mmol, 1.0 equiv.) and allyl bromide (0.20 mL, 2.30 mmol, 1.1 equiv.) *via* General Procedure D, and was obtained as a pale yellow solid (356 mg, 67%).

MP: 58-60 °C; $\mathbf{R_f}$: ~0.45 (4:1 (v/v) cyclohexane-EtOAc); ^{1}**H NMR** (400 MHz, 298 K, $CDCl_3$): δ = 7.45 (d, *J* = 5.6 Hz, 1H), 7.14 (d, *J* = 5.6 Hz, 1H), 5.75 (ddt, *J* = 17.0, 10.0, 7.0 Hz, 1H), 5.09 – 4.99 (m, 2H), 4.83 (t, *J* = 7.5 Hz, 1H), 4.23 – 4.11 (m, 2H), 2.83 (dtt, *J* = 14.0, 7.0, 1.5 Hz, 1H), 2.63 – 2.55 (m, 1H), 1.24 ppm (t, *J* = 7.0 Hz, 3H); ^{13}**C NMR** (126 MHz, 298 K, $CDCl_3$): δ = 171.7, 148.1, 141.0, 134.2, 130.4, 129.0, 118.1, 61.5, 44.5, 37.0, 14.3 ppm; **IR** (film): ṽ = 1736, 1643, 1542, 1504, 1400, 1338, 1277, 1187, 1121, 1033 cm^{-1}; **HRMS** (ESI-TOF) calculated for $[C_{11}H_{14}NO_4S]^+$ $[M+H]^+$: 256.0638, found 256.0640.

***rac*-Ethyl 2-(2-(*N*-(methylsulfonyl)methylsulfonamido)thiophen-3-yl)propanoate (37a)**

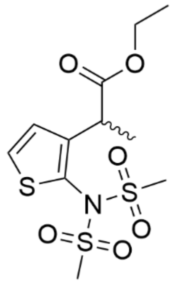

Thiophene **37a** was prepared from ethyl 2-(2-nitrothiophen-3-yl)propanoate **14a** (1.13 g, 4.93 mmol, 1.0 equiv.) and methanesulfonyl chloride (1.1 mL, 14.8 mmol, 3.0 equiv.) *via* General Procedure A, and was obtained as a brown solid (406 mg, 23%).

MP: 110-112 °C; $\mathbf{R_f}$: ~0.50 (7:3 (v/v) cyclohexane-EtOAc); ^{1}**H NMR** (400 MHz, 298 K, $CDCl_3$): δ = 7.37 (d, *J* = 5.8 Hz, 1H), 7.14 (d, *J* = 5.8 Hz, 1H), 4.13 (q, *J* = 7.0 Hz, 2H), 3.84 (q, *J* = 7.0 Hz, 1H), 3.60 (s, 3H), 3.42 (s, 3H), 1.50 (d, *J* = 7.0 Hz, 3H), 1.21 ppm (t, *J* = 7.0 Hz, 3H); ^{13}**C NMR** (126 MHz, 298 K, $CDCl_3$): δ = 172.9, 143.1, 128.3, 127.6, 126.2, 61.5, 43.0, 41.9, 39.3, 18.1, 14.2 ppm; **IR** (film): ṽ = 1736, 1420, 1374, 1324, 1207, 1167, 1099, 1077 cm^{-1}; **HRMS** (ESI-TOF)

calculated for $[C_{11}H_{18}NO_6S_3]^+$ $[M+H]^+$: 356.0291, found 356.0292.

***rac*-Ethyl 2-(2-(*N*-(methylsulfonyl)methylsulfonamido)thiophen-3-yl)pentanoate (37b)**

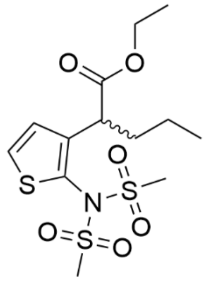

Thiophene **37b** was prepared from ethyl 2-(2-nitrothiophen-3-yl)pentanoate **14b** (2.70 g, 10.5 mmol, 1.0 equiv.) and methanesulfonyl chloride (2.4 mL, 31.5 mmol, 3.0 equiv.) *via* General Procedure A, and was obtained as a yellow solid (868 mg, 22%).

MP: 102-105 °C; $\mathbf{R_f}$**:** ~0.55 (7:3 (v/v) cyclohexane-EtOAc); **^{1}H NMR** (500 MHz, 298 K, $CDCl_3$): δ = 7.36 (d, *J* = 5.9 Hz, 1H), 7.18 (d, *J* = 5.9 Hz, 1H), 4.13 (q, *J* = 7.0 Hz, 2H), 3.71 (dd, *J* = 10.0, 5.5 Hz, 1H), 3.62 (s, 3H), 3.43 (s, 3H), 2.07 (dtd, *J* = 13.5, 10.0, 5.0 Hz, 1H), 1.76 – 1.67 (m, 1H), 1.46 – 1.29 (m, 2H), 1.22 (t, *J* = 7.0 Hz, 3H), 0.94 ppm (t, *J* = 7.5 Hz, 3H); **^{13}C NMR** (126 MHz, 298 K, $CDCl_3$): δ = 172.5, 142.2, 128.4, 127.3, 126.7, 61.4, 45.1, 43.0, 42.0, 35.4, 21.2, 14.2, 13.9 ppm; **IR** (film): ṽ = 1734, 1419, 1374, 1326, 1242, 1170, 1101, 1030 cm^{-1}; **HRMS** (ESI-TOF) calculated for $[C_{13}H_{22}NO_6S_3]^+$ $[M+H]^+$: 384.0604, found 384.0604.

***rac*-Ethyl 3-methyl-2-(2-(*N*-(methylsulfonyl)methylsulfonamido)thiophen-3-yl)butanoate (37c)**

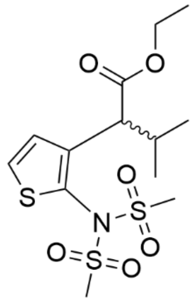

Thiophene **37c** was prepared from ethyl 3-methyl-2-(2-nitrothiophen-3-yl)butanoate **14c** (2.90 g, 11.3 mmol, 1.0 equiv.) and methanesulfonyl chloride (2.6 mL, 33.9 mmol, 3.0 equiv.) *via* General Procedure A, and was obtained as a light brown solid (742 mg, 17%).

MP: 97-99 °C; $\mathbf{R_f}$**:** ~0.55 (7:3 (v/v) cyclohexane-EtOAc); **^{1}H NMR** (500 MHz, 298 K, $CDCl_3$): δ = 7.37 (d, *J* = 5.9 Hz, 1H), 7.21 (d, *J* = 5.9 Hz, 1H), 4.12 (q, *J* = 7.0 Hz, 2H), 3.66 (s, 3H), 3.46 (s, 3H), 3.36 (d, *J* = 10.5 Hz, 1H), 2.43 (dp, *J* = 10.5, 6.5 Hz, 1H), 1.22 (t, *J* = 7.0 Hz, 3H), 1.06 (d, *J* = 6.5 Hz, 3H), 0.82 ppm (d, *J* = 7.0 Hz, 3H); **^{13}C NMR** (126 MHz, 298 K, $CDCl_3$): δ = 172.2,

141.5, 129.3, 127.1, 126.8, 61.2, 52.9, 43.2, 41.8, 32.2, 21.3, 20.5, 14.2 ppm; **IR** (film): ṽ = 1731, 1420, 1374, 1325, 1243, 1200, 1167, 1123, 1097, 1032 cm^{-1}; **HRMS** (ESI-TOF) calculated for $[C_{13}H_{22}NO_6S_3]^+$ $[M+H]^+$: 384.0604, found 384.0615.

***rac*-Ethyl 2-(2-(*N*-(methylsulfonyl)methylsulfonamido)thiophen-3-yl)-3-phenylpropanoate (37d)**

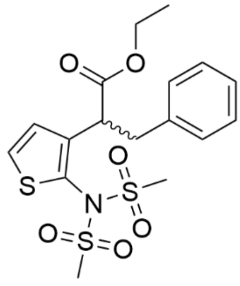

Thiophene **37d** was prepared from ethyl 2-(2-nitrothiophen-3-yl)-3-phenylpropanoate **14d** (1.30 g, 4.26 mmol, 1.0 equiv.) and methanesulfonyl chloride (0.99 mL, 12.8 mmol, 3.0 equiv.) *via* General Procedure A, and was obtained as a light orange solid (558 mg, 30%).

MP: 108-110 °C; **R_f:** ~0.40 (9:1 (v/v) cyclohexane-EtOAc); **^{1}H NMR** (400 MHz, 298 K, $CDCl_3$): δ = 7.42 (d, *J* = 5.9 Hz, 1H), 7.32 – 7.20 (m, 6H), 4.04 (qd, *J* = 7.0, 3.0 Hz, 2H), 3.96 – 3.90 (m, 1H), 3.56 (s, 3H), 3.37 (s, 3H), 3.34 – 3.28 (m, 1H), 3.04 (dd, *J* = 13.5, 4.0 Hz, 1H), 1.11 ppm (t, *J* = 7.0 Hz, 3H); **^{13}C NMR** (126 MHz, 298 K, $CDCl_3$): δ = 171.6, 141.5, 138.9, 129.2 (2C), 128.7 (2C), 128.6, 127.7, 126.9, 126.5, 61.4, 47.9, 43.0, 41.9, 39.2, 14.1 ppm; **IR** (film): ṽ = 1736, 1375, 1325, 1259, 1208, 1169, 1039 cm^{-1}; **HRMS** (ESI-TOF) calculated for $[C_{17}H_{22}NO_6S_3]^+$ $[M+H]^+$: 432.0604, found 432.0606.

***rac*-Ethyl 4-cyano-2-(2-(*N*-(methylsulfonyl)methylsulfonamido)thiophen-3-yl)butanoate (37e)**

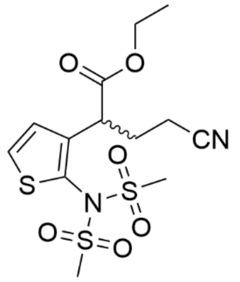

Thiophene **37e** was prepared from ethyl 4-cyano-2-(2-nitrothiophen-3-yl)butanoate **14e** (735 mg, 2.74 mmol, 1.0 equiv.) and methanesulfonyl chloride (0.64 mL, 8.22 mmol, 3.0 equiv.) *via* General Procedure A, and was obtained as a yellow solid (270 mg, 25%).

MP: 131-133 °C; **R_f:** ~0.45 (7:3 (v/v) cyclohexane-EtOAc); **^{1}H NMR** (500 MHz, 298 K, $CDCl_3$):

δ = 7.43 (d, *J* = 5.9 Hz, 1H), 7.10 (d, *J* = 5.9 Hz, 1H), 4.16 (qd, *J* = 7.0, 1.0 Hz, 2H), 3.83 – 3.79 (m, 1H), 3.62 (s, 3H), 3.46 (s, 3H), 2.46 – 2.32 (m, 3H), 2.23 – 2.15 (m, 1H), 1.23 ppm (t, *J* = 7.0 Hz, 3H); **^{13}C NMR** (126 MHz, 298 K, $CDCl_3$): δ = 170.9, 139.7, 129.9, 128.1, 125.7, 118.9, 62.1, 43.8, 43.1, 41.9, 28.4, 15.6, 14.1 ppm; **IR** (film): ṽ = 2250, 1734, 1422, 1374, 1325, 1232, 1217, 1167, 1098, 1043 cm^{-1}; **HRMS** (ESI-TOF) calculated for $[C_{13}H_{19}N_2O_6S_3]^+$ $[M+H]^+$: 395.0400, found 395.0410.

rac-Ethyl 2-(2-(*N*-(methylsulfonyl)methylsulfonamido)thiophen-3-yl)pent-4-enoate (37f)

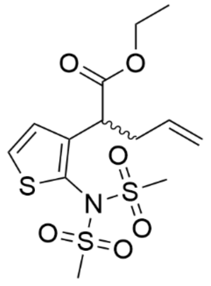

Thiophene **37f** was prepared from ethyl 2-(2-nitrothiophen-3-yl)pent-4-enoate **14f** (354 mg, 1.39 mmol, 1.0 equiv.) and methanesulfonyl chloride (0.32 mL, 4.17 mmol, 3.0 equiv.) *via* General Procedure A, and was obtained as a yellow amorphous solid (52.0 mg, 10%).

R_f: ~0.50 (3:2 (v/v) cyclohexane-EtOAc); **^{1}H NMR** (400 MHz, 298 K, $CDCl_3$): δ = 7.38 (d, *J* = 5.9 Hz, 1H), 7.19 (d, *J* = 5.9 Hz, 1H), 5.79 (dddd, *J* = 16.5, 10.0, 7.5, 6.5 Hz, 1H), 5.19 – 5.02 (m, 2H), 4.14 (q, *J* = 7.0 Hz, 2H), 3.77 (dd, *J* = 10.0, 5.0 Hz, 1H), 3.60 (s, 3H), 3.43 (s, 3H), 2.80 (dddt, *J* = 14.0, 10.0, 7.5, 1.0 Hz, 1H), 2.50 (dddt, *J* = 14.0, 6.5, 5.0, 1.5 Hz, 1H), 1.22 ppm (t, *J* = 7.0 Hz, 3H); **^{13}C NMR** (126 MHz, 298 K, $CDCl_3$): δ = 171.7, 141.4, 134.8, 128.7, 127.4, 126.6, 117.8, 61.5, 45.2, 43.0, 42.0, 37.3, 14.2 ppm; **IR** (film): ṽ = 1736, 1420, 1374, 1324, 1237, 1168 cm^{-1}; **HRMS** (ESI-TOF) calculated for $[C_{13}H_{20}NO_6S_3]^+$ $[M+H]^+$: 382.0447, found 382.0451.

rac-2-(2-(Methylsulfonamido)thiophen-3-yl)propanoic acid (15a)

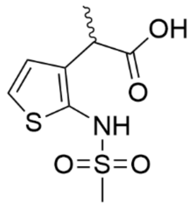

Thiophene **15a** was prepared from ethyl 2-(2-(*N*-(methylsulfonyl)methylsulfonamido)thiophen-3-yl)propanoate **37a** (20.0 mg, 0.056 mmol) *via* General Procedure B, and was obtained as a yellow amorphous solid (12.5 mg, 89%).

R_f: ~0.30 (7:3 (v/v) cyclohexane-EtOAc + 0.1% formic acid); **^{1}H NMR** (400 MHz, 298 K,

acetone-d_6): δ = 8.10 (s, 1H), 7.29 (d, *J* = 5.8 Hz, 1H), 7.02 (d, *J* = 5.8 Hz, 1H), 4.10 (q, *J* = 7.0 Hz, 1H), 3.08 (s, 3H), 1.42 ppm (d, *J* = 7.0 Hz, 3H); **^{13}C NMR** (126 MHz, 298 K, acetone-d_6): δ = 175.0, 138.3, 133.7, 126.4, 123.9, 39.7, 38.4, 18.6 ppm; **IR** (film): ṽ = 3581, 3234, 1710, 1435, 1406, 1324, 1237, 1210, 1150, 1098 cm^{-1}; **HRMS** (ESI-TOF) calculated for $[C_8H_{10}NO_4S_2]^-$ $[M-H]^-$: 248.0057, found 248.0066.

***rac*-2-(2-(Methylsulfonamido)thiophen-3-yl)pentanoic acid (15b)**

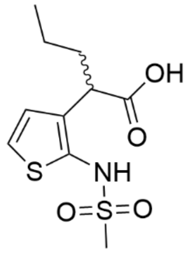

Thiophene **15b** was prepared from ethyl 2-(2-(*N*-(methylsulfonyl)methylsulfonamido)thiophen-3-yl)pentanoate **37b** (770 mg, 2.01 mmol) *via* General Procedure B, and was obtained as a yellow amorphous solid (486 mg, 87%).

R$_f$: ~0.40 (3:2 (v/v) cyclohexane-EtOAc + 0.1% formic acid); **^{1}H NMR** (400 MHz, 298 K, acetone-d_6): δ = 8.55 (s, 1H), 7.29 (d, *J* = 5.8 Hz, 1H), 7.04 (d, *J* = 5.8 Hz, 1H), 3.99 (dd, *J* = 8.5, 7.0 Hz, 1H), 3.08 (s, 3H), 2.03 – 1.95 (m, 1H), 1.69 (dddd, *J* = 13.0, 10.0, 7.0, 5.5 Hz, 1H), 1.46 – 1.20 (m, 2H), 0.90 ppm (t, *J* = 7.5 Hz, 3H); **^{13}C NMR** (126 MHz, 298 K, acetone-d_6): δ = 174.5, 136.8, 134.2, 126.6, 123.7, 44.2, 39.8, 36.1, 21.4, 14.2 ppm; **IR** (film): ṽ = 3574, 3241, 1708, 1556, 1437, 1404, 1328, 1245, 1197, 1152, 1104 cm^{-1}; **HRMS** (ESI-TOF) calculated for $[C_{10}H_{16}NO_4S_2]^+$ $[M+H]^+$: 278.0515, found 278.0516.

***rac*-3-Methyl-2-(2-(methylsulfonamido)thiophen-3-yl)butanoic acid (15c)**

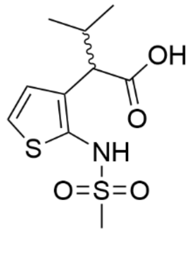

Thiophen **15c** was prepared from ethyl 3-methyl-2-(2-(*N*-(methylsulfonyl)methylsulfonamido)thiophen-3-yl)butanoate **37c** (660 mg, 1.72 mmol) *via* General Procedure B, and was obtained as a yellow amorphous solid (367 mg, 77%).

R$_f$: ~0.40 (3:2 (v/v) cyclohexane-EtOAc + 0.1% formic acid); **^{1}H NMR** (500 MHz, 298 K, acetone-d_6): δ = 8.48 (s, 1H), 7.28 (d, *J* = 5.8 Hz, 1H), 7.06 (d, *J* = 5.8 Hz, 1H), 3.66 (d, *J* = 10.5

Hz, 1H), 3.08 (s, 3H), 2.37 – 2.25 (m, 1H), 1.06 (d, *J* = 6.5 Hz, 3H), 0.75 ppm (d, *J* = 7.0 Hz, 3H); **^{13}C NMR** (126 MHz, 298 K, acetone-*d$_6$*): δ = 174.3, 135.1, 135.0, 126.8, 123.5, 52.1, 40.0, 32.3, 21.5, 20.2 ppm; **IR** (film): ṽ = 3541, 3229, 1704, 1553, 1466, 1402, 1326, 1261, 1152, 1033 cm^{-1}; **HRMS** (ESI-TOF) calculated for $[C_{10}H_{16}NO_4S_2]^+$ $[M+H]^+$: 278.0515, found 278.0524.

***rac*-2-(2-(Methylsulfonamido)thiophen-3-yl)-3-phenylpropanoic acid (15d)**

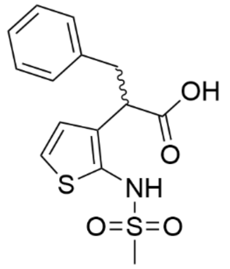

Thiophene **15d** was prepared from ethyl 2-(2-(*N*-(methylsulfonyl)methylsulfonamido)thiophen-3-yl)-3-phenylpropanoate **37d** (558 mg, 1.29 mmol) *via* General Procedure B, and was obtained as an orange amorphous solid (308 mg, 73%).

R$_f$: ~0.40 (7:3 (v/v) cyclohexane-EtOAc + 0.1% formic acid); **^{1}H NMR** (500 MHz, 298 K, acetone-*d$_6$*): δ = 8.44 (s, 1H), 7.32 (d, *J* = 5.8 Hz, 1H), 7.29 – 7.23 (m, 4H), 7.19 – 7.15 (m, 2H), 4.31 (dd, *J* = 9.5, 6.0 Hz, 1H), 3.32 (dd, *J* = 13.5, 9.5 Hz, 1H), 3.04 (dd, *J* = 13.5, 6.0 Hz, 1H), 2.97 ppm (s, 3H); **^{13}C NMR** (126 MHz, 298 K, acetone-*d$_6$*): δ = 173.8, 140.3, 136.0, 134.5, 130.0 (2C), 129.1 (2C), 127.2, 126.6, 123.8, 46.6, 39.6, 39.5 ppm; **IR** (film): ṽ = 3579, 3224, 1710, 1496, 1455, 1401, 1321, 1240, 1151, 1095 cm^{-1}; **HRMS** (ESI-TOF) calculated for $[C_{14}H_{14}NO_4S_2]^-$ $[M-H]^-$: 324.0370, found 324.0380.

***rac*-4-Cyano-2-(2-(methylsulfonamido)thiophen-3-yl)butanoic acid (15e)**

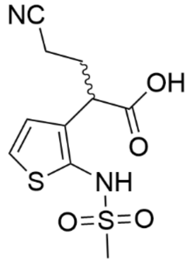

Thiophene **15e** was prepared from ethyl 4-cyano-2-(2-(*N*-(methylsulfonyl)methylsulfonamido)thiophen-3-yl)butanoate **37e** (405 mg, 1.03 mmol) *via* General Procedure B, and was obtained as a yellow amorphous solid (278 mg, 94%).

R$_f$: ~0.35 (3:2 (v/v) cyclohexane-EtOAc + 0.1% formic acid); **^{1}H NMR** (400 MHz, 298 K, acetone-*d$_6$*): δ = 8.11 (s, 1H), 7.36 (d, *J* = 5.8 Hz, 1H), 7.03 (d, *J* = 5.8 Hz, 1H), 4.11 (t, *J* = 7.5 Hz,

1H), 3.12 (s, 3H), 2.57 – 2.33 (m, 3H), 2.17 – 2.09 ppm (m, 1H); **^{13}C NMR** (126 MHz, 298 K, acetone-*d*$_6$): δ = 173.3, 162.3, 135.3, 126.1, 124.6, 120.1, 43.2, 39.9, 30.6, 15.5 ppm; **IR** (film): ṽ = 3580, 3235, 2252, 1720, 1632, 1556, 1428, 1406, 1324, 1233, 1150, 1096 cm^{-1}; **HRMS** (ESI-TOF) calculated for $[C_{10}H_{11}N_2O_4S_2]^-$ [M-H]$^-$: 287.0166, found 287.0179.

***rac*-2-(2-(Methylsulfonamido)thiophen-3-yl)pent-4-enoic acid (15f)**

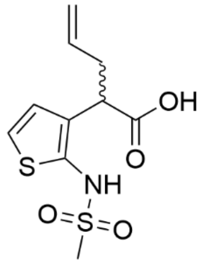

Thiophene **15f** was prepared from ethyl 2-(2-(*N*-(methylsulfonyl)methylsulfonamido)thiophen-3-yl)pent-4-enoate **37f** (50.0 mg, 0.131 mmol) *via* General Procedure B, and was obtained as a pale white amorphous solid (32.6 mg, 90%).

R$_f$: ~0.30 (7:3 (v/v) cyclohexane-EtOAc + 0.1% formic acid); **^{1}H NMR** (400 MHz, 298 K, acetone-*d*$_6$): δ = 8.51 (s, 1H), 7.29 (d, *J* = 5.8 Hz, 1H), 7.05 (d, *J* = 5.8 Hz, 1H), 5.80 (ddt, *J* = 17.0, 10.0, 7.0 Hz, 1H), 5.08 (dq, *J* = 17.0, 1.5 Hz, 1H), 4.97 (ddt, *J* = 10.0, 2.0, 1.0 Hz, 1H), 4.08 (dd, *J* = 8.5, 6.5 Hz, 1H), 3.08 (s, 3H), 2.76 (dddt, *J* = 14.0, 8.5, 7.0, 1.0 Hz, 1H), 2.53 – 2.44 ppm (m, 1H); **^{13}C NMR** (126 MHz, 298 K, acetone-*d*$_6$): δ = 173.8, 136.4, 136.0, 134.4, 126.6, 123.8, 117.2, 44.2, 39.8, 37.8 ppm; **IR** (film): ṽ = 3542, 3233, 1712, 1555, 1437, 1376, 1324, 1234, 1194, 1151, 1096 cm^{-1}; **HRMS** (ESI-TOF) calculated for $[C_{10}H_{12}NO_4S_2]^-$ [M-H]$^-$: 274.0213, found 274.0221.

***rac*-4-Methyl-6-(methylsulfonyl)-4,6-dihydro-5*H*-thieno[2,3-*b*]pyrrol-5-one (16a)**

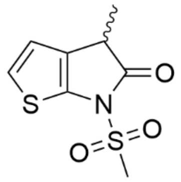

γ-Lactam **16a** was prepared from 2-(2-(methylsulfonamido)thiophen-3-yl)propanoic acid **15a** (180 mg, 0.722 mmol) *via* General Procedure C, and was obtained as a yellow solid (120 mg, 72%).

MP: 124-126 °C; **R$_f$:** ~0.60 (1:1 (v/v) cyclohexane-EtOAc); **^{1}H NMR** (500 MHz, 298 K, CDCl$_3$): δ = 6.95 (d, *J* = 5.3 Hz, 1H), 6.84 (d, *J* = 5.3 Hz, 1H), 3.68 (q, *J* = 7.5 Hz, 1H), 3.38 (s, 3H), 1.52 ppm (d, *J* = 7.5 Hz, 3H); **^{13}C NMR** (126 MHz, 298 K, CDCl$_3$): δ = 178.8, 137.4, 127.1, 120.6, 120.4, 42.8, 41.0, 15.9 ppm; **IR** (film): ṽ = 1761, 1441, 1360, 1291, 1256, 1166, 1068, 1019 cm^{-1}; **HRMS** (ESI-TOF) calculated for $[C_8H_9NO_3S_2Na]^+$ [M+Na]$^+$: 253.9916, found 253.9917.

4,4-Dimethyl-6-(methylsulfonyl)-4,6-dihydro-5*H*-thieno[2,3-*b*]pyrrol-5-one (17a)

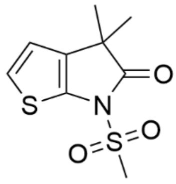

γ-Lactam **17a** was prepared from 6-(methylsulfonyl)-4,6-dihydro-5*H*-thieno[2,3-*b*]pyrrol-5-one **7** (50.0 mg, 0.230 mmol, 1.0 equiv.) and iodomethane (0.03 mL, 0.506 mmol, 2.2 equiv.) *via* General Procedure E, and was obtained as an off-white amorphous solid (5.10 mg, 9%).

R_f: ~0.50 (1:1 (v/v) cyclohexane-EtOAc); **^{1}H NMR** (400 MHz, 298 K, $CDCl_3$): δ = 7.18 (d, *J* = 5.9 Hz, 1H), 6.93 (d, *J* = 5.9 Hz, 1H), 3.74 (s, 3H), 3.15 (s, 3H), 3.04 ppm (s, 3H); **^{13}C NMR** (126 MHz, 298 K, $CDCl_3$): δ = 176.6, 143.9, 137.1, 126.5, 123.6, 52.4, 44.7, 41.2, 35.6 ppm; **IR** (film): ṽ = 1732, 1468, 1436, 1347, 1281, 1255, 1027 cm^{-1}; **HRMS** (ESI-TOF) for [$C_9H_{11}NO_3S_2$]: not observed.

***rac*-6-(Methylsulfonyl)-4-propyl-4,6-dihydro-5*H*-thieno[2,3-*b*]pyrrol-5-one (16b)**

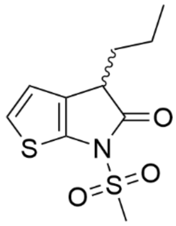

γ-Lactam **16b** was prepared from 2-(2-(methylsulfonamido)thiophen-3-yl)pentanoic acid **15b** (433 mg, 1.56 mmol) *via* General Procedure C, and was obtained as a pale orange solid (307 mg, 76%).

MP: 68-70 °C; **R_f:** ~0.50 (4:1 (v/v) cyclohexane-EtOAc); **^{1}H NMR** (400 MHz, 298 K, $CDCl_3$): δ = 6.95 (d, *J* = 5.3 Hz, 1H), 6.85 (d, *J* = 5.3 Hz, 1H), 3.66 (dd, *J* = 8.0, 5.5 Hz, 1H), 3.38 (s, 3H), 2.04 – 1.94 (m, 1H), 1.81 (dddd, *J* = 13.5, 10.0, 8.0, 5.5 Hz, 1H), 1.45 (dddd, *J* = 14.5, 13.0, 10.0, 7.5 Hz, 2H), 0.96 ppm (t, *J* = 7.5 Hz, 3H); **^{13}C NMR** (126 MHz, 298 K, $CDCl_3$): δ = 178.3, 137.8, 125.8, 121.1, 120.2, 47.9, 41.0, 33.2, 19.8, 13.9 ppm; **IR** (film): ṽ = 1756, 1445, 1364, 1292, 1169, 1083, 1054, 1019 cm^{-1}; **HRMS** (ESI-TOF) calculated for [$C_{10}H_{13}NO_3S_2Na$]$^+$ [M+Na]$^+$: 282.0229, found 282.0239.

rac-4-Isopropyl-6-(methylsulfonyl)-4,6-dihydro-5H-thieno[2,3-b]pyrrol-5-one (16c)

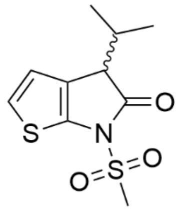

γ-Lactam **16c** was prepared from 3-methyl-2-(2-(methylsulfonamido)thiophen-3-yl)butanoic acid **15c** (342 mg, 1.23 mmol) *via* General Procedure C, and was obtained as a yellow solid (234 mg, 73%).

MP: 76-78 °C; **R_f:** ~0.50 (4:1 (v/v) cyclohexane-EtOAc); **^{1}H NMR** (400 MHz, 298 K, $CDCl_3$): δ = 6.96 (d, *J* = 5.3 Hz, 1H), 6.86 (d, *J* = 5.3 Hz, 1H), 3.60 (d, *J* = 4.0 Hz, 1H), 3.37 (s, 3H), 2.47 (hd, *J* = 7.0, 4.0 Hz, 1H), 1.17 (d, *J* = 7.0 Hz, 3H), 0.83 ppm (d, *J* = 7.0 Hz, 3H); **^{13}C NMR** (126 MHz, 298 K, $CDCl_3$): δ = 177.8, 138.4, 123.8, 121.5, 120.2, 54.4, 41.0, 31.1, 20.5, 18.0 ppm; **IR** (film): ṽ = 1758, 1443, 1366, 1293, 1236, 1172, 1086, 1004 cm^{-1}; **HRMS** (ESI-TOF) calculated for $[C_{10}H_{13}NO_3S_2Na]^+$ $[M+Na]^+$: 282.0229, found 282.0241.

rac-4-Benzyl-6-(methylsulfonyl)-4,6-dihydro-5H-thieno[2,3-b]pyrrol-5-one (16d)

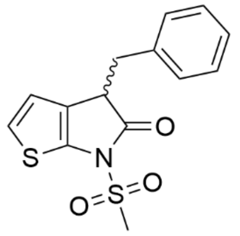

γ-Lactam **16d** was prepared from 2-(2-(methylsulfonamido)thiophen-3-yl)-3-phenylpropanoic acid **15d** (294 mg, 0.904 mmol) *via* General Procedure C, and was obtained as an orange solid (173 mg, 62%).

MP: 99-102 °C; **R_f:** ~0.40 (4:1 (v/v) cyclohexane-EtOAc); **^{1}H NMR** (500 MHz, 298 K, $CDCl_3$): δ = 7.27 – 7.19 (m, 3H), 7.08 – 7.04 (m, 2H), 6.89 (d, *J* = 5.3 Hz, 1H), 6.54 (d, *J* = 5.3 Hz, 1H), 3.97 (dd, *J* = 8.5, 4.5 Hz, 1H), 3.40 (dd, *J* = 13.5, 4.5 Hz, 1H), 3.07 (dd, *J* = 13.5, 8.5 Hz, 1H), 3.03 ppm (s, 3H); **^{13}C NMR** (126 MHz, 298 K, $CDCl_3$): δ = 177.1, 138.3, 136.4, 129.6 (2C), 128.6 (2C), 127.3, 124.8, 121.3, 120.2, 49.4, 40.4, 37.0 ppm; **IR** (film): ṽ = 1754, 1455, 1364, 1292, 1169, 1082, 1033 cm^{-1}; **HRMS** (ESI-TOF) calculated for $[C_{14}H_{14}NO_3S_2]^+$ $[M+H]^+$: 308.0410, found 308.0412.

***rac*-3-(6-(Methylsulfonyl)-5-oxo-5,6-dihydro-4*H*-thieno[2,3-*b*]pyrrol-4-yl)propanenitrile (16e)**

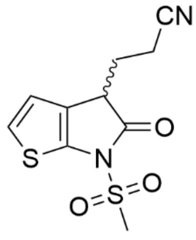

γ-Lactam **16e** was prepared from 4-cyano-2-(2-(methylsulfonamido)thiophen-3-yl)butanoic acid **15e** (90.0 mg, 0.312 mmol) *via* General Procedure C, and was obtained as a light orange amorphous solid (51.0 mg, 60%).

R$_f$: ~0.50 (1:1 (v/v) cyclohexane-EtOAc); **^{1}H NMR** (400 MHz, 298 K, $CDCl_3$): δ = 7.02 (d, *J* = 5.3 Hz, 1H), 6.87 (d, *J* = 5.3 Hz, 1H), 3.82 (dd, *J* = 7.0, 6.0 Hz, 1H), 3.42 (s, 3H), 2.64 – 2.49 (m, 2H), 2.38 – 2.24 ppm (m, 2H); **^{13}C NMR** (126 MHz, 298 K, acetone-*d*$_6$): δ = 177.7, 139.4, 125.2, 122.0, 121.2, 119.8, 47.1, 41.0, 27.2, 14.7 ppm; **IR** (film): ṽ = 2249, 1739, 1447, 1362, 1293, 1260, 1169, 1089, 1072 cm^{-1}; **HRMS** (ESI-TOF) calculated for $[C_{10}H_{11}N_2O_3S_2]^+$ $[M+H]^+$: 271.0206, found 271.0207.

***rac*-4-Allyl-6-(methylsulfonyl)-4,6-dihydro-5*H*-thieno[2,3-*b*]pyrrol-5-one (16f)**

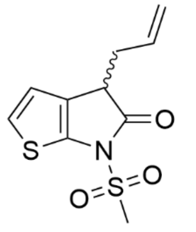

γ-Lactam **16f** was prepared from 2-(2-(methylsulfonamido)thiophen-3-yl)pent-4-enoic acid **15f** (28.0 mg, 0.102 mmol) *via* General Procedure C, and was obtained as a pale yellow amorphous solid (12.0 mg, 46%).

R$_f$: ~0.55 (3:2 (v/v) cyclohexane-EtOAc); **^{1}H NMR** (500 MHz, 298 K, $CDCl_3$): δ = 6.94 (d, *J* = 5.3 Hz, 1H), 6.86 (d, *J* = 5.3 Hz, 1H), 5.73 (dddd, *J* = 17.0, 10.0, 8.0, 6.0 Hz, 1H), 5.18 – 5.11 (m, 2H), 3.74 (dd, *J* = 8.0, 5.0 Hz, 1H), 3.36 (s, 3H), 2.83 (dddt, *J* = 14.0, 6.0, 5.0, 1.5 Hz, 1H), 2.54 ppm (dtt, *J* = 14.0, 8.0, 1.0 Hz, 1H); **^{13}C NMR** (126 MHz, 298 K, $CDCl_3$): δ = 177.48, 138.04, 133.12, 125.17, 121.18, 120.26, 119.10, 47.71, 41.03, 35.22 ppm; **IR** (film): ṽ = 1741, 1465, 1366, 1329, 1262, 1167, 1097, 1026 cm^{-1}; **HRMS** (ESI-TOF) calculated for $[C_{10}H_{12}NO_3S_2]^+$ $[M+H]^+$: 258.0253, found 258.0252.

4,4-Diallyl-6-(methylsulfonyl)-4,6-dihydro-5*H*-thieno[2,3-*b*]pyrrol-5-one (17b)

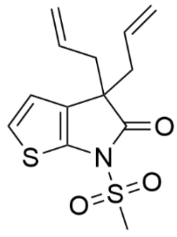

γ-Lactam **17b** was prepared from 6-(methylsulfonyl)-4,6-dihydro-5*H*-thieno[2,3-*b*]pyrrol-5-one **7** (30.0 mg, 0.138 mmol, 1.0 equiv.) and allyl bromide (0.03 mL, 0.304 mmol, 2.2 equiv.) *via* General Procedure E, and was obtained as a pale white solid (37.0 mg, 90%). The analytical data for **17b** are consistent with those reported.[10]

MP: 124-128 °C; **R$_f$:** ~0.65 (1:1 (v/v) cyclohexane-EtOAc); **^{1}H NMR** (400 MHz, 298 K, $CDCl_3$): δ = 6.96 (d, *J* = 5.3 Hz, 1H), 6.82 (d, *J* = 5.3 Hz, 1H), 5.51 – 5.40 (m, 2H), 5.10 – 5.01 (m, 4H), 3.28 (s, 3H), 2.61 – 2.56 ppm (m, 4H); **^{13}C NMR** (151 MHz, 298 K, $CDCl_3$): δ = 179.5, 137.2, 131.7 (2C), 128.0, 120.5, 120.4, 120.0 (2C), 56.6, 41.3 (2C), 41.0 ppm; **IR** (film): ṽ = 1758, 1641, 1538, 1444, 1368, 1290, 1263, 1173, 1085, 1022 cm^{-1}; **HRMS** (ESI-TOF) calculated for $[C_{13}H_{16}NO_3S_2]^+$ $[M+H]^+$: 298.0566, found 298.0571.

6'-(Methylsulfonyl)spiro[cyclopentane-1,4'-thieno[2,3-*b*]pyrrol]-3-en-5'(6'*H*)-one (18)

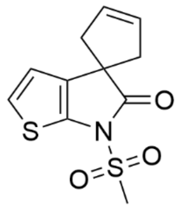

γ-Lactam **18** was prepared from 4,4-diallyl-6-(methylsulfonyl)-4,6-dihydro-5*H*-thieno[2,3-*b*]pyrrol-5-one **17b** (10.0 mg, 0.034 mmol) *via* General Procedure F, and was obtained as a white solid (3.00 mg, 33%). To our knowledge, analytical data for **18** have not been previously provided.[10]

MP: 126-129 °C; **R$_f$:** ~0.50 (3:2 (v/v) cyclohexane-EtOAc); **^{1}H NMR** (400 MHz, 298 K, $CDCl_3$): δ = 6.92 (d, *J* = 5.3 Hz, 1H), 6.78 (d, *J* = 5.3 Hz, 1H), 5.80 (s, 2H), 3.40 (s, 3H), 3.02 (d, *J* = 15.5 Hz, 2H), 2.63 ppm (d, *J* = 15.0 Hz, 2H); **^{13}C NMR** (126 MHz, 298 K, $CDCl_3$): δ = 182.0, 135.9, 132.9, 128.4 (2C), 120.9, 119.4, 55.3, 44.8 (2C), 41.1 ppm; **IR** (film): ṽ = 1758, 1442, 1397, 1365, 1292, 1172, 1082, 1034 cm^{-1}; **HRMS** (ESI-TOF) calculated for $[C_{11}H_{11}NO_3S_2K]^+$ $[M+K]^+$: 307.9812, found 307.9818.

6,6-Diallyl-4-(methylsulfonyl)-4,6-dihydro-5*H*-thieno[3,2-*b*]pyrrol-5-one (38)

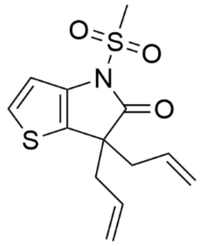

γ-Lactam **38** was prepared from 4-(methylsulfonyl)-4,6-dihydro-5*H*-thieno[3,2-*b*]pyrrol-5-one **8** (50.0 mg, 0.230 mmol, 1.0 equiv.) and allyl bromide (0.05 mL, 0.575 mmol, 2.5 equiv.) *via* General Procedure E, and was obtained as a pale yellow solid (19.0 mg, 28%).

MP: 89-92 °C; **R_f:** ~0.45 (4:1 (v/v) cyclohexane-EtOAc); **^{1}H NMR** (400 MHz, 298 K, $CDCl_3$): δ = 7.30 (d, *J* = 5.2 Hz, 1H), 7.23 (d, *J* = 5.2 Hz, 1H), 5.50 (ddt, *J* = 17.5, 10.0, 7.5 Hz, 2H), 5.14 – 5.04 (m, 4H), 3.27 (s, 3H), 2.59 ppm (dt, *J* = 7.5, 1.0 Hz, 4H); **^{13}C NMR** (126 MHz, 298 K, $CDCl_3$): δ = 180.0, 138.5, 131.4 (2C), 127.4, 123.4, 120.5 (2C), 115.5, 56.7, 41.9 (2C), 41.2 ppm; **IR** (film): ṽ = 1758, 1450, 1416, 1368, 1324, 1250, 1175, 1084, 1057 cm^{-1}; **HRMS** (ESI-TOF) calculated for $[C_{13}H_{16}NO_3S_2]^+$ $[M+H]^+$: 298.0566, found 298.0575.

4'-(Methylsulfonyl)spiro[cyclopentane-1,6'-thieno[3,2-*b*]pyrrol]-3-en-5'(4'*H*)-one (19)

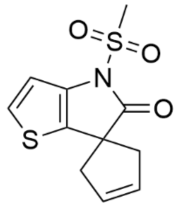

γ-Lactam **19** was prepared from 6,6-diallyl-4-(methylsulfonyl)-4,6-dihydro-5*H*-thieno[3,2-*b*]pyrrol-5-one **38** (14.0 mg, 0.047 mmol) *via* General Procedure F, and was obtained as a white solid (7.50 mg, 59%).

MP: 130-133 °C; **R_f:** ~0.25 (4:1 (v/v) cyclohexane-EtOAc); **^{1}H NMR** (400 MHz, 298 K, acetone-*d_6*): δ = 7.43 (d, *J* = 5.2 Hz, 1H), 7.14 (d, *J* = 5.2 Hz, 1H), 5.86 – 5.81 (m, 2H), 3.48 (s, 3H), 3.04 – 2.96 (m, 2H), 2.69 – 2.62 ppm (m, 2H); **^{13}C NMR** (151 MHz, 298 K, acetone-*d_6*): δ = 182.4, 138.8, 129.1 (2C), 128.8, 126.9, 116.0, 56.4, 46.3 (2C), 41.2 ppm; **IR** (film): ṽ = 1757, 1448, 1390, 1364, 1329, 1209, 1175, 1084, 1036 cm^{-1}; **HRMS** (ESI-TOF) calculated for $[C_{11}H_{12}NO_3S_2]^+$ $[M+H]^+$: 270.0253, found 270.0254.

2-(2-Bromothiophen-3-yl)acetic acid (21)

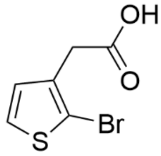

Thiophene **21** was synthesized according to a reported procedure.[22] *N*-Bromosuccinimide (3.01 g, 16.9 mmol, 1.2 equiv.) was added in 3 portions to a solution of 2-(thiophen-3-yl)acetic acid (**20**) (2.00 g, 14.1 mmol, 1.0 equiv.) in anhydrous THF (20 mL) at 0 °C over 10 min. The reaction mixture was stirred at 0 °C for 4 h, and at rt for 14 h, then concentrated *in vacuo*. The crude material was purified using flash column chromatography (0-30% (v/v) EtOAc in cyclohexane supplemented with 0.1% (v/v) formic acid using normal phase silica gel, followed by 20-50% (v/v) CH_3CN in water supplemented with 0.1% (v/v) formic acid using reversed-phase silica gel) to afford the desired product **21** as a white solid (2.50 g, 80%). The analytical data for **21** are consistent with those reported.[23]

MP: 68-70 °C; $\mathbf{R_f}$**:** ~0.35 (3:2 (v/v) cyclohexane-EtOAc + 0.1% formic acid); ^{1}**H NMR** (400 MHz, 298 K, acetone-d_6): δ = 10.88 (s, 1H), 7.48 (d, *J* = 5.6 Hz, 1H), 7.04 (d, *J* = 5.6 Hz, 1H), 3.65 ppm (s, 2H); ^{13}**C NMR** (126 MHz, 298 K, acetone-d_6): δ = 171.1, 135.7, 130.2, 126.9, 111.3, 35.0 ppm; **IR** (film): ṽ = 3112, 1711, 1411, 1303, 1246, 1223, 1160, 1094 cm^{-1}; **HRMS** (ESI-TOF) calculated for $[C_6H_4BrO_2S]^-$ $[M-H]^-$: 218.9121, found 218.9119.

2-(2-Acetamidothiophen-3-yl)acetic acid (22a)

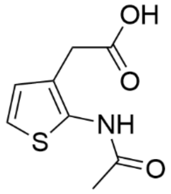

Thiophene **22a** was prepared from 2-(2-bromothiophen-3-yl)acetic acid **21** (200 mg, 0.905 mmol, 1.0 equiv.) using acetamide (58.8 mg, 0.996 mmol, 1.1 equiv.) *via* General Procedure H, and was obtained as a light green solid (9.70 mg, 5%).

MP: 125-127 °C; $\mathbf{R_f}$**:** ~0.20 (2:3 (v/v) cyclohexane-EtOAc + 0.1% formic acid); ^{1}**H NMR** (400 MHz, 298 K, acetone-d_6): δ = 9.72 (s, 1H), 6.90 (d, *J* = 5.5 Hz, 1H), 6.80 (d, *J* = 5.5 Hz, 1H), 3.63 (s, 2H), 2.12 ppm (s, 3H); ^{13}**C NMR** (126 MHz, 298 K, acetone-d_6): δ = 172.7, 167.8, 136.6, 127.4, 119.2, 117.8, 33.7, 22.9 ppm; **IR** (film): ṽ = 3248, 2928, 1685, 1619, 1590, 1320, 1264, 1218, 1026 cm^{-1}; **HRMS** (ESI-TOF) calculated for $[C_8H_{10}NO_3S]^+$ $[M+H]^+$: 200.0376, found 200.0377.

2-(2-(Phenylsulfonamido)thiophen-3-yl)acetic acid (22c)

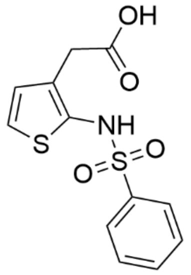

Thiophene **22c** was prepared from 2-(2-bromothiophen-3-yl)acetic acid **21** (500 mg, 2.26 mmol, 1.0 equiv.) using benzenesulfonamide (889 mg, 5.65 mmol, 2.5 equiv.) *via* General Procedure G, and was obtained as a yellow solid (283 mg, 42%).

MP: 167-170 °C; **R_f:** ~0.20 (1:1 (v/v) cyclohexane-EtOAc + 0.1% formic acid); **^{1}H NMR** (500 MHz, 298 K, acetone-*d_6*): δ = 10.73 (s, 1H), 8.85 (s, 1H), 7.79 – 7.75 (m, 2H), 7.71 – 7.66 (m, 1H), 7.60 – 7.54 (m, 2H), 7.16 (d, *J* = 5.7 Hz, 1H), 6.92 (d, *J* = 5.7 Hz, 1H), 3.43 ppm (s, 2H); **^{13}C NMR** (126 MHz, 298 K, acetone-*d_6*): δ = 171.9, 140.4, 134.4, 134.0, 132.1, 130.0 (2C), 128.9, 128.3 (2C), 123.2, 32.8 ppm; **IR** (film): ṽ = 3595, 3235, 1704, 1448, 1331, 1257, 1162, 1092 cm^{-1}; **HRMS** (ESI-TOF) calculated for $[C_{12}H_{12}NO_4S_2]^+$ $[M+H]^+$: 298.0202, found 298.0204.

2-(2-((4-Methoxyphenyl)sulfonamido)thiophen-3-yl)acetic acid (22d)

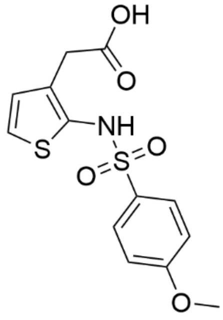

Thiophene **22d** was prepared from 2-(2-bromothiophen-3-yl)acetic acid **21** (200 mg, 0.905 mmol, 1.0 equiv.) using 4-methoxybenzenesulfonamide (423 mg, 2.26 mmol, 2.5 equiv.) *via* General Procedure G, and was obtained as a dark yellow amorphous solid (23.0 mg, 8%).

R_f: ~0.30 (1:1 (v/v) cyclohexane-EtOAc + 0.1% formic acid); **^{1}H NMR** (400 MHz, 298 K, acetone-*d_6*): δ = 8.69 (s, 1H), 7.71 – 7.66 (m, 2H), 7.15 (d, *J* = 5.7 Hz, 1H), 7.10 – 7.05 (m, 2H), 6.91 (d, *J* = 5.7 Hz, 1H), 3.89 (s, 3H), 3.45 ppm (s, 2H); **^{13}C NMR** (126 MHz, 298 K, acetone-*d_6*): δ = 172.1, 164.2, 134.8, 131.8, 131.8, 130.5 (2C), 128.8, 123.1, 115.0 (2C), 56.1, 32.9 ppm; **IR** (film): ṽ = 3530, 3253, 1706, 1596, 1498, 1415, 1329, 1262, 1184, 1157, 1093 cm^{-1}; **HRMS** (ESI-TOF) calculated for $[C_{13}H_{12}NO_5S_2]^-$ $[M-H]^-$: 326.0162, found 326.0167.

2-(2-((4-Nitrophenyl)sulfonamido)thiophen-3-yl)acetic acid (22e)

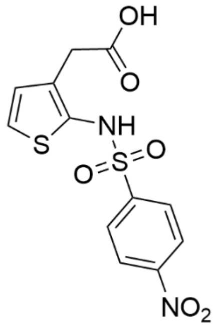

Thiophene **22e** was prepared from 2-(2-bromothiophen-3-yl)acetic acid **21** (200 mg, 0.905 mmol, 1.0 equiv.) using 4-nitrobenzenesulfonamide (201 mg, 0.996 mmol, 1.1 equiv.) *via* General Procedure H, and was obtained as a light green solid (76.0 mg, 24%).

MP: 166-169 °C; **R_f:** ~0.40 (3:2 (v/v) cyclohexane-EtOAc + 0.1% formic acid); **^{1}H NMR** (400 MHz, 298 K, acetone-*d*$_6$): δ = 8.45 – 8.39 (m, 2H), 8.07 – 8.00 (m, 2H), 7.21 (d, *J* = 5.7 Hz, 1H), 6.94 (d, *J* = 5.7 Hz, 1H), 3.46 ppm (s, 2H); **^{13}C NMR** (126 MHz, 298 K, acetone-*d*$_6$): δ = 171.7, 151.4, 145.9, 133.5, 132.3, 129.9 (2C), 129.1, 125.3 (2C), 123.6, 32.9 ppm; **IR** (film): ṽ = 3487, 3294, 1710, 1531, 1405, 1350, 1311, 1167, 1090 cm^{-1}; **HRMS** (ESI-TOF) calculated for $[C_{12}H_9N_2O_6S_2]^-$ $[M\text{-}H]^-$: 340.9908, found 340.9905.

2-(2-((4-(Trifluoromethyl)phenyl)sulfonamido)thiophen-3-yl)acetic acid (22f)

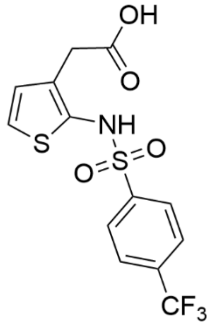

Thiophene **22f** was prepared from 2-(2-bromothiophen-3-yl)acetic acid **21** (200 mg, 0.905 mmol, 1.0 equiv.) using 4-trifluoromethylbenzenesulfonamide (224 mg, 0.996 mmol, 1.1 equiv.) *via* General Procedure H, and was obtained as a dark yellow solid (145 mg, 44%).

MP: 160-162 °C; **R_f:** ~0.30 (3:2 (v/v) cyclohexane-EtOAc + 0.1% formic acid); **^{1}H NMR** (400 MHz, 298 K, acetone-*d*$_6$): δ = 8.02 – 7.93 (m, 4H), 7.19 (d, *J* = 5.7 Hz, 1H), 6.95 (d, *J* = 5.7 Hz, 1H), 3.48 ppm (s, 2H); **^{19}F NMR** (470 MHz, 298 K, acetone-*d*$_6$): δ = −63.6 ppm (s, 3F); **^{13}C NMR** (126 MHz, 298 K, acetone-*d*$_6$): δ = 171.8, 144.2, 134.8 (q, *J* = 32.5 Hz), 133.6, 132.5, 129.2 (2C), 129.1, 127.2 (q, *J* = 4.0 Hz, 2C), 124.5 (q, *J* = 272.0 Hz), 123.6, 32.9 ppm; **IR** (film): ṽ = 3527, 3256, 1714, 1529, 1407, 1376, 1324, 1261, 1170, 1136, 1064 cm^{-1}; **HRMS** (ESI-TOF) calculated

for $[C_{13}H_9F_3NO_4S_2]^-$ $[M-H]^-$: 363.9931, found 363.9932.

6-Acetyl-4,6-dihydro-5*H*-thieno[2,3-*b*]pyrrol-5-one (23a)

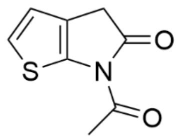

γ-Lactam **23a** was prepared from 2-(2-acetamidothiophen-3-yl)acetic acid **22a** (75.0 mg, 0.376 mmol) *via* General Procedure C, and was obtained as a light pink solid (35.0 mg, 51%).

MP: 136-138 °C; **R$_f$:** ~0.45 (1:1 (v/v) cyclohexane-EtOAc); **^{1}H NMR** (600 MHz, 298 K, $CDCl_3$): δ = 7.02 (d, *J* = 5.3 Hz, 1H), 6.86 (d, *J* = 5.3 Hz, 1H), 3.74 (s, 2H), 2.63 ppm (s, 3H); **^{13}C NMR** (126 MHz, 298 K, $CDCl_3$): δ = 175.6, 167.4, 139.8, 121.3, 120.7, 120.4, 37.6, 24.0 ppm; **IR** (film): ṽ = 1774, 1687, 1677, 1408, 1335, 1264, 1237, 1190, 1084, 1022 cm^{-1}; **HRMS** (ESI-TOF) calculated for $[C_8H_8NO_2S]^+$ $[M+H]^+$: 182.0270, found 182.0272.

Ethyl 2-(2-((*N*-(benzylsulfonyl)-1-phenylmethyl)sulfonamido)thiophen-3-yl)acetate (39)

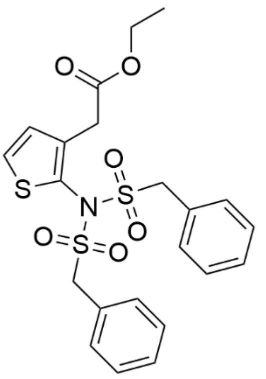

Thiophene **39** was prepared from ethyl 2-(2-nitrothiophen-3-yl)acetate **13** (1.13 g, 5.25 mmol, 1.0 equiv.) and phenylmethanesulfonyl chloride (3.01 g, 15.8 mmol, 3.0 equiv.) *via* General Procedure A, and was obtained as a light green solid (567 mg, 22%).

MP: 120-122 °C; **R$_f$:** ~0.40 (7:3 (v/v) cyclohexane-EtOAc); **^{1}H NMR** (400 MHz, 298 K, $CDCl_3$): δ = 7.51 – 7.46 (m, 4H), 7.44 – 7.38 (m, 6H), 7.23 (d, *J* = 5.8 Hz, 1H), 6.98 (d, *J* = 5.8 Hz, 1H), 5.00 (d, *J* = 13.5 Hz, 2H), 4.88 (d, *J* = 13.5 Hz, 2H), 4.15 (q, *J* = 7.0 Hz, 2H), 3.49 (s, 2H), 1.23 ppm (t, *J* = 7.0 Hz, 3H); **^{13}C NMR** (126 MHz, 298 K, $CDCl_3$): δ = 170.1, 136.6, 131.6 (4C), 130.6, 129.7 (2C), 129.3 (4C), 128.0 (2C), 127.4, 126.6, 61.5, 61.2 (2C), 34.0, 14.3 ppm; **IR** (film): ṽ = 1734, 1457, 1379, 1357, 1203, 1167, 1142, 1032 cm^{-1}; **HRMS** (ESI-TOF) calculated for $[C_{22}H_{24}NO_6S_3]^+$ $[M+H]^+$: 494.0760, found 494.0771.

2-(2-((Phenylmethyl)sulfonamido)thiophen-3-yl)acetic acid (22b)

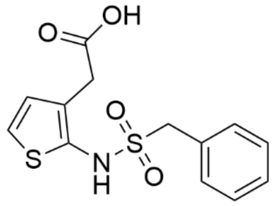

Thiophene **22b** was prepared from ethyl 2-(2-((*N*-(benzylsulfonyl)-1-phenylmethyl)sulfonamido)thiophen-3-yl)acetate **39** (542 mg, 1.10 mmol) *via* General Procedure B, and was obtained as a light brown amorphous solid (168 mg, 49%).

R_f: ~0.35 (2:3 (v/v) cyclohexane-EtOAc + 0.1% formic acid); **^{1}H NMR** (500 MHz, 298 K, acetone-d_6): δ = 8.55 (s, 1H), 7.47 – 7.43 (m, 2H), 7.41 – 7.34 (m, 3H), 7.24 (d, *J* = 5.7 Hz, 1H), 7.00 (d, *J* = 5.7 Hz, 1H), 4.48 (s, 2H), 3.68 ppm (s, 2H); **^{13}C NMR** (126 MHz, 298 K, acetone-d_6): δ = 172.2, 135.0, 131.9 (2C), 131.0, 130.4, 129.3 (2C), 129.2, 129.0, 122.7, 58.2, 33.3 ppm; **IR** (film): ṽ = 3572, 3237, 1710, 1496, 1407, 1328, 1255, 1202, 1154 cm^{-1}; **HRMS** (ESI-TOF) calculated for $[C_{13}H_{12}NO_4S_2]^-$ [M-H]$^-$: 310.0213, found 310.0220.

6-(Benzylsulfonyl)-4,6-dihydro-5*H*-thieno[2,3-*b*]pyrrol-5-one (23b)

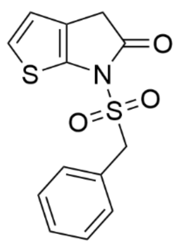

γ-Lactam **23b** was prepared from 2-(2-((phenylmethyl)sulfonamido)thiophen-3-yl)acetic acid **22b** (162 mg, 0.520 mmol) *via* General Procedure C, and was obtained as a light brown solid (39.0 mg, 26%).

MP: 122-124 °C; **R_f:** ~0.90 (2:3 (v/v) cyclohexane-EtOAc); **^{1}H NMR** (400 MHz, 298 K, $CDCl_3$): δ = 7.38 – 7.33 (m, 2H), 7.32 – 7.27 (m, 3H), 6.79 (d, *J* = 5.3 Hz, 1H), 6.70 (d, *J* = 5.3 Hz, 1H), 4.75 (s, 2H), 3.60 ppm (s, 2H); **^{13}C NMR** (101 MHz, 298 K, $CDCl_3$): δ = 175.3, 139.8, 130.7 (2C), 129.6, 129.1 (2C), 126.5, 120.6, 119.9, 119.8, 59.2, 36.9 ppm; **IR** (film): ṽ = 1750, 1450, 1366, 1289, 1202, 1155, 1088, 1072 cm^{-1}; **HRMS** (ESI-TOF) calculated for $[C_{13}H_{11}NO_3S_2Na]^+$ [M+Na]$^+$: 316.0073, found 316.0074.

6-(Phenylsulfonyl)-4,6-dihydro-5*H*-thieno[2,3-*b*]pyrrol-5-one (23c)

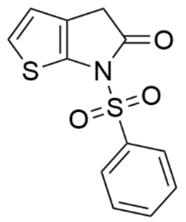

γ-Lactam **23c** was prepared from 2-(2-(phenylsulfonamido)thiophen-3-yl)acetic acid **22c** (283 mg, 0.952 mmol) *via* General Procedure C, and was obtained as a light pink solid (128 mg, 48%).

MP: 141-144 °C; **R_f:** ~0.40 (7:3 (v/v) cyclohexane-EtOAc); **^{1}H NMR** (400 MHz, 298 K, $CDCl_3$): δ = 8.12 – 8.07 (m, 2H), 7.70 – 7.65 (m, 1H), 7.59 – 7.51 (m, 2H), 6.95 (d, *J* = 5.3 Hz, 1H), 6.80 (d, *J* = 5.3 Hz, 1H), 3.53 ppm (s, 2H); **^{13}C NMR** (101 MHz, 298 K, $CDCl_3$): δ = 174.1, 139.4, 137.4, 134.8, 129.4 (2C), 128.2 (2C), 121.3, 120.8, 120.1, 37.0 ppm; **IR** (film): ṽ = 1766, 1585, 1450, 1376, 1291, 1185, 1161, 1078 cm^{-1}; **HRMS** (ESI-TOF) calculated for $[C_{12}H_{13}N_2O_3S_2]^+$ $[M+NH_4]^+$: 297.0362, found 297.0370.

6-((4-Methoxyphenyl)sulfonyl)-4,6-dihydro-5*H*-thieno[2,3-*b*]pyrrol-5-one (23d)

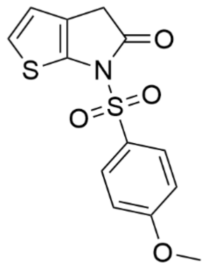

γ-Lactam **23d** was prepared from 2-(2-((4-methoxyphenyl)sulfonamido)thiophen-3-yl)acetic acid **22d** (17.0 mg, 0.052 mmol) *via* General Procedure C, and was obtained as a pale white solid (10.8 mg, 67%).

MP: 155-158 °C; **R_f:** ~0.55 (1:1 (v/v) cyclohexane-EtOAc); **^{1}H NMR** (500 MHz, 298 K, $CDCl_3$): δ = 8.05 – 8.00 (m, 2H), 7.01 – 6.96 (m, 2H), 6.94 (d, *J* = 5.3 Hz, 1H), 6.79 (d, *J* = 5.3 Hz, 1H), 3.87 (s, 3H), 3.52 ppm (s, 2H); **^{13}C NMR** (126 MHz, 298 K, $CDCl_3$): δ = 174.2, 164.7, 139.6, 130.6 (2C), 128.8, 121.3, 120.6, 119.9, 114.6 (2C), 55.9, 37.0 ppm; **IR** (film): ṽ = 1760, 1595, 1498, 1373, 1266, 1168, 1092, 1024 cm^{-1}; **HRMS** (ESI-TOF) calculated for $[C_{13}H_{11}NO_4S_2Na]^+$ $[M+Na]^+$: 332.0022, found 332.0025.

6-((4-Nitrophenyl)sulfonyl)-4,6-dihydro-5*H*-thieno[2,3-*b*]pyrrol-5-one (23e)

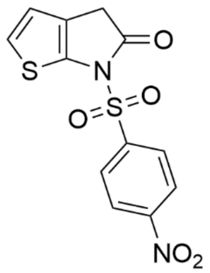

γ-Lactam **23e** was prepared from 2-(2-((4-nitrophenyl)sulfonamido)thiophen-3-yl)acetic acid **22e** (38.0 mg, 0.111 mmol) *via* General Procedure C, and was obtained as a dark yellow solid (8.00 mg, 22%).

MP: 120-123 °C; **R$_f$:** ~0.50 (3:2 (v/v) cyclohexane-EtOAc); ^{1}**H NMR** (400 MHz, 298 K, $CDCl_3$): δ = 8.43 – 8.35 (m, 2H), 8.32 – 8.27 (m, 2H), 7.00 (d, *J* = 5.3 Hz, 1H), 6.83 (d, *J* = 5.3 Hz, 1H), 3.57 ppm (s, 2H); ^{13}**C NMR** (126 MHz, 298 K, $CDCl_3$): δ = 174.0, 151.4, 142.6, 138.2, 129.6 (2C), 124.7 (2C), 121.5, 121.4, 120.6, 36.9 ppm; **IR** (film): ṽ = 1748, 1608, 1532, 1438, 1351, 1314, 1261, 1168, 1087, 1035 cm^{-1}; **HRMS** (ESI-TOF) calculated for $[C_{12}H_8N_2O_5S_2Na]^+$ $[M+Na]^+$: 346.9767, found 346.9766.

6-((4-(Trifluoromethyl)phenyl)sulfonyl)-4,6-dihydro-5*H*-thieno[2,3-*b*]pyrrol-5-one (23f)

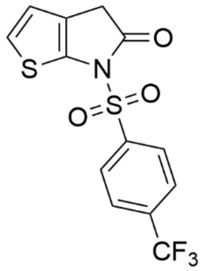

γ-Lactam **23f** was prepared from 2-(2-((4-(trifluoromethyl)phenyl)sulfonamido)thiophen-3-yl)acetic acid **22f** (310 mg, 0.848 mmol) *via* General Procedure C, and was obtained as a light pink solid (127 mg, 43%).

MP: 148-150 °C; **R$_f$:** ~0.50 (3:2 (v/v) cyclohexane-EtOAc); ^{1}**H NMR** (400 MHz, 298 K, $CDCl_3$): δ = 8.23 (d, *J* = 8.5 Hz, 2H), 7.82 (d, *J* = 8.5 Hz, 2H), 6.98 (d, *J* = 5.3 Hz, 1H), 6.82 (d, *J* = 5.3 Hz, 1H), 3.56 ppm (s, 2H); ^{19}**F NMR** (376 MHz, 298 K, $CDCl_3$): δ = −63.3 ppm (s, 3F); ^{13}**C NMR** (101 MHz, 298 K, $CDCl_3$): δ = 174.0, 140.7, 138.7, 136.3 (q, *J* = 33.5 Hz), 128.8 (2C), 126.6 (q, *J* = 3.5 Hz, 2C), 123.1 (q, *J* = 274.5 Hz), 121.4, 121.2, 120.4, 37.0 ppm; **IR** (film): ṽ = 1775, 1407, 1385, 1324, 1263, 1183, 1142, 1110, 1064, 1017 cm^{-1}; **HRMS** (ESI-TOF) calculated for $[C_{13}H_9F_3NO_3S_2]^+$ $[M+H]^+$: 347.9971, found 347.9974.

4. References

(1) Gill, A. L.; Harris, W. Thienopyrrolidinones. US 20020028841 A1, **2002**.

(2) Freyne, E. J. E.; Lacrampe, J. F. A.; Deroose, F. D.; Venet, M. G. Interleukin-5 inhibiting 6-azauracil derivatives. EP 0987265 A1, **2000**.

(3) Sircar, I.; Gudmundsson, K. S.; Martin, R. Inhibitors of alpha 4 mediated cell adhesion. WO 9936393 A1, **1999**.

(4) Dudash Jr, J.; Jiang, J.; Mayer, S. C.; Joullié, M. M. Comparative Study of Selected Coupling Reagents in Dipeptide Synthesis. *Synth. Commun.* **1993**, *23* (3), 349-356.

(5) Crimmins, M. T.; King, B. W. An Efficient Asymmetric Approach to Carbocyclic Nucleosides: Asymmetric Synthesis of 1592U89, a Potent Inhibitor of HIV Reverse Transcriptase. *J. Org. Chem.* **1996**, *61* (13), 4192-4193.

(6) Coutts, I. G. C.; Hamblin, M. Synthesis of NN-diaryltoluene-4-sulphonamides. *J. Chem. Soc., Perkin Trans. 1* **1975**, (23), 2445-2446.

(7) Klapars, A.; Huang, X.; Buchwald, S. L. A General and Efficient Copper Catalyst for the Amidation of Aryl Halides. *J. Am. Chem. Soc.* **2002**, *124* (25), 7421-7428.

(8) Makosza, M.; Kwast, E. Vicarious nucleophilic substitution of hydrogen in nitroderivatives of five-membered heteroaromatic compounds. *Tetrahedron* **1995**, *51* (30), 8339-8354.

(9) Beers, S. A.; Malloy, E. A.; Wu, W.; Wachter, M.; Ansell, J.; Singer, M.; Steber, M.; Barbone, A.; Kirchner, T.; Ritchie, D.; Argentieri, D. N-(5-substituted) thiophene-2-alkylsulfonamides as potent inhibitors of 5-lipoxygenase. *Biorg. Med. Chem.* **1997**, *5* (4), 779-786.

(10) Migaud, M. E.; Wilmouth, R. C.; Mills, G. I.; Wayne, G. J.; Risley, C.; Chambers, C.; Macdonald, S. J. F.; Schofield, C. J. 5,5-Fused thiophene γ-lactams as templates for serine protease inhibition. *Chem. Commun.* **2002**, (12), 1274-1275.

(11) Michael, J. P.; de Koning, C. B.; van der Westhuyzen, C. W.; Fernandes, M. A. Influence of ring size on the outcome of sulfide contraction reactions with thiolactams. Isolation of bicyclic ketene S,N-acetals and thioisomünchnones. *J. Chem. Soc., Perkin Trans. 1* **2001**, (17), 2055-2062.

(12) Hu, X.-Q.; Han, J.-B.; Zhang, C.-P. Cu-Mediated Trifluoromethylation of Aromatic α-Diazo Esters with the Yagupolskii–Umemoto Reagent. *Eur. J. Org. Chem.* **2017**, *2017* (2), 324-331.

(13) Kenda, B.; Turet, L.; Quesnel, L.; Michel, P.; Ates, A. New heterocyclic derivatives useful for the treatment of CNS disorders. WO 2008132142 A2, **2008**.

(14) Jagtap, P.; Pham-Huu, D.-P.; Cohen, F.; Hu, H.; Wang, X. Substituted tetracyclic 1H-indeno (1,2-b) pyridine-2 (5H)-one analogs thereof and uses thereof. WO 2010077663 A2, **2010**.

(15) Ames, D. E.; Ribeiro, O. Heterocyclic syntheses from o-halogeno-acids. Part II.

Thienopyridinones and thienopyranones from 3-bromothiophen-2- and 4-bromothiophen-3-carboxylic acids. *J. Chem. Soc., Perkin Trans. 1* **1975**, (14), 1390-1395.
(16) Chen, Z.; Ginn, J. D.; Hickey, E. R.; Liu, W.; Mao, C.; Morwick, T. M.; Nemoto, P. A.; Spero, D.; Sun, S. Substituted benzothiophene compounds and uses thereof. WO 2005012283 A1, **2005**.
(17) Kondo, Y.; Tanabe, H.; Kudo, H.; Nakano, K.; Otake, T. Electrochromic Type E-Paper Using Poly(1H-Thieno[3,4-d]Imidazol-2(3H)-One) Derivatives by a Novel Printing Fabrication Process. *Materials* **2011**, *4* (12), 2171-2182.
(18) Tapia, I.; Alonso-Cires, L.; López-Tudanca, P. L.; Mosquera, R.; Labeaga, L.; Innerárity, A.; Orjales, A. 2,3-Dihydro-2-oxo-1H-benzimidazole-1-carboxamides with Selective Affinity for the 5-HT4 Receptor: Synthesis and Structure−Affinity and Structure−Activity Relationships of a New Series of Partial Agonist and Antagonist Derivatives. *J. Med. Chem.* **1999**, *42* (15), 2870-2880.
(19) Galvez, C.; Garcia, F.; Garcia, J.; Soldevila, J. Synthesis of thiophenedicarbonyldiazides and Di-t-butyl thiophendicarbamates. *J. Heterocycl. Chem.* **1986**, *23* (4), 1103-1108.
(20) Kondou, Y.; Nakano, K.; Otake, T. pi-electron conjugated compound, manufacturing method therefor, and pi-electron conjugated polymer obtained using same. US 8519150 B2, **2013**.
(21) Reddy, P. A. P.; Wong, T. T.; Zhao, L.; Tang, S.; Labroli, M. A.; Guzi, T. J.; Siddiqui, M. A. Thiazole derivatives as protein kinase inhibitors. WO 2009058728 A1, **2009**.
(22) Vallat, P.; Lamps, J. P.; Schosseler, F.; Rawiso, M.; Catala, J. M. Quasi-Controlled Polymerization through a Nickel Catalyst Process of a Functionalized Thiophene Monomer: Kinetic Studies and Application to the Synthesis of Regioregular Poly(thiophene-3-acetic acid). *Macromolecules* **2007**, *40* (7), 2600-2602.
(23) Heerklotz, J.; Linden, A.; Hesse, M. Synthesis of Aza-1,2-thiophenophanes by Double Ring Enlargement. *Tetrahedron* **2000**, *56* (37), 7205-7210.
(24) Bolli, M.; Boss, C.; Clozel, M.; Fischli, W.; Weller, T. Novel alkansulfonamides as endothelin antagonists. WO 03055863 A1, **2003**.
(25) Chambers, M. S.; Fletcher, S. R.; Matassa, V. G. Benzodiazepine derivatives, compositions containing them and their use in therapy. EP 0514133 A1, **1992**.
(26) Yang, L.-Q.; Wang, K.-B.; Li, C.-Y. Synthesis of oxindoles through the gold-catalyzed oxidation of *N*-arylynamides. *Eur. J. Org. Chem.* **2013**, 2775-2779.

5. ^{1}H and ^{13}C NMR spectra of thiophene-fused γ-lactam SARS-CoV-2 M^{pro} inhibitors prepared for this study

γ-Lactam **7**

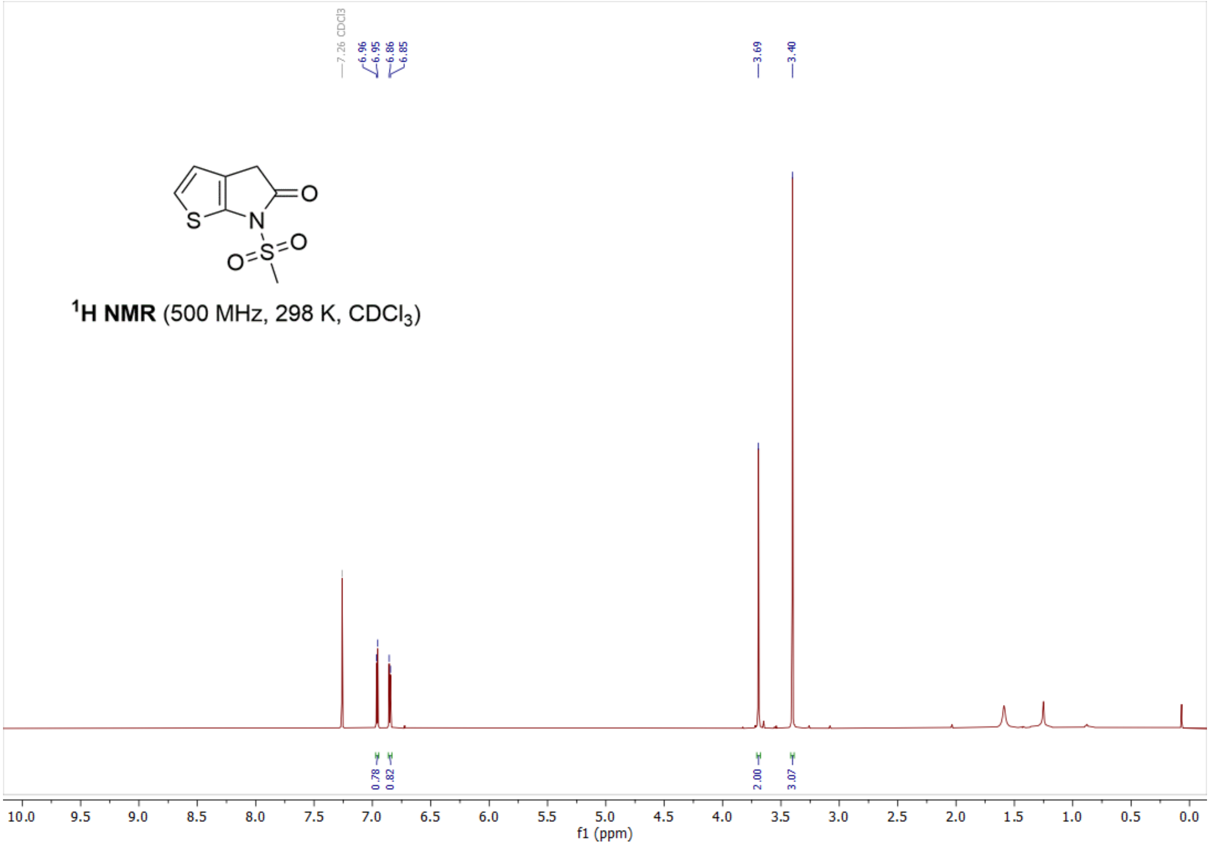

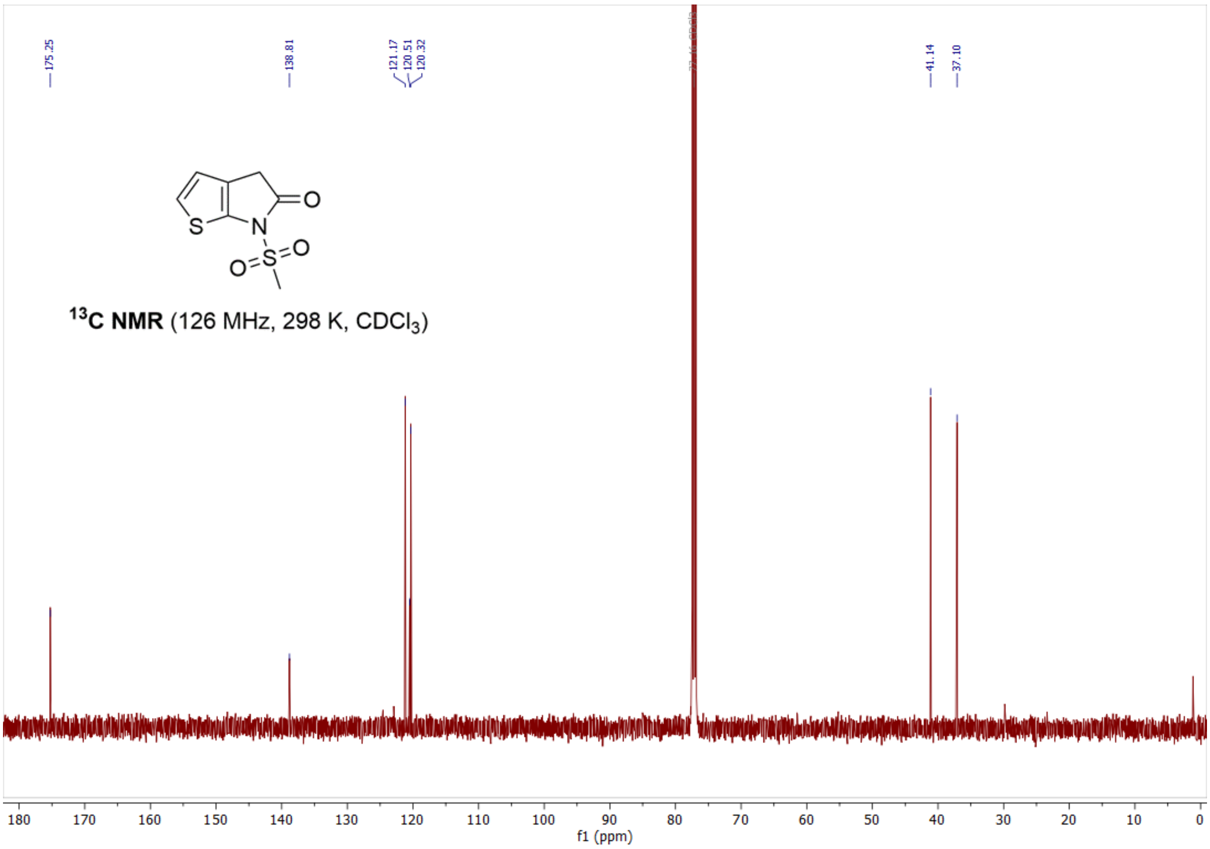

γ-Lactam **8**

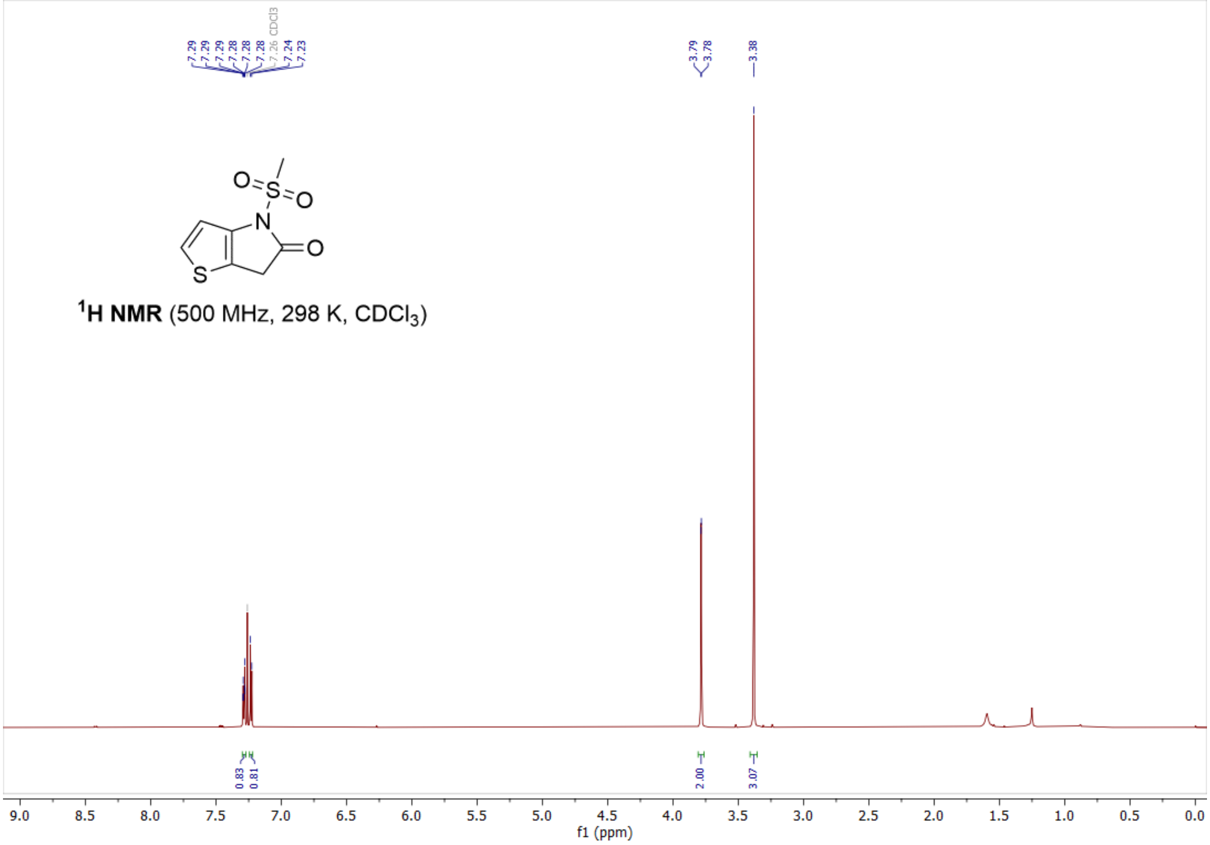

7.29
7.29
7.29
7.28
7.28
7.28
7.26 CDCl3
7.24
7.23
3.79
3.78
3.38
¹H NMR (500 MHz, 298 K, CDCl₃)
0.83
0.81
2.00
3.07
9.0
8.5
8.0
7.5
7.0
6.5
6.0
5.5
5.0
4.5
4.0
3.5
3.0
2.5
2.0
1.5
1.0
0.5
0.0
f1 (ppm)

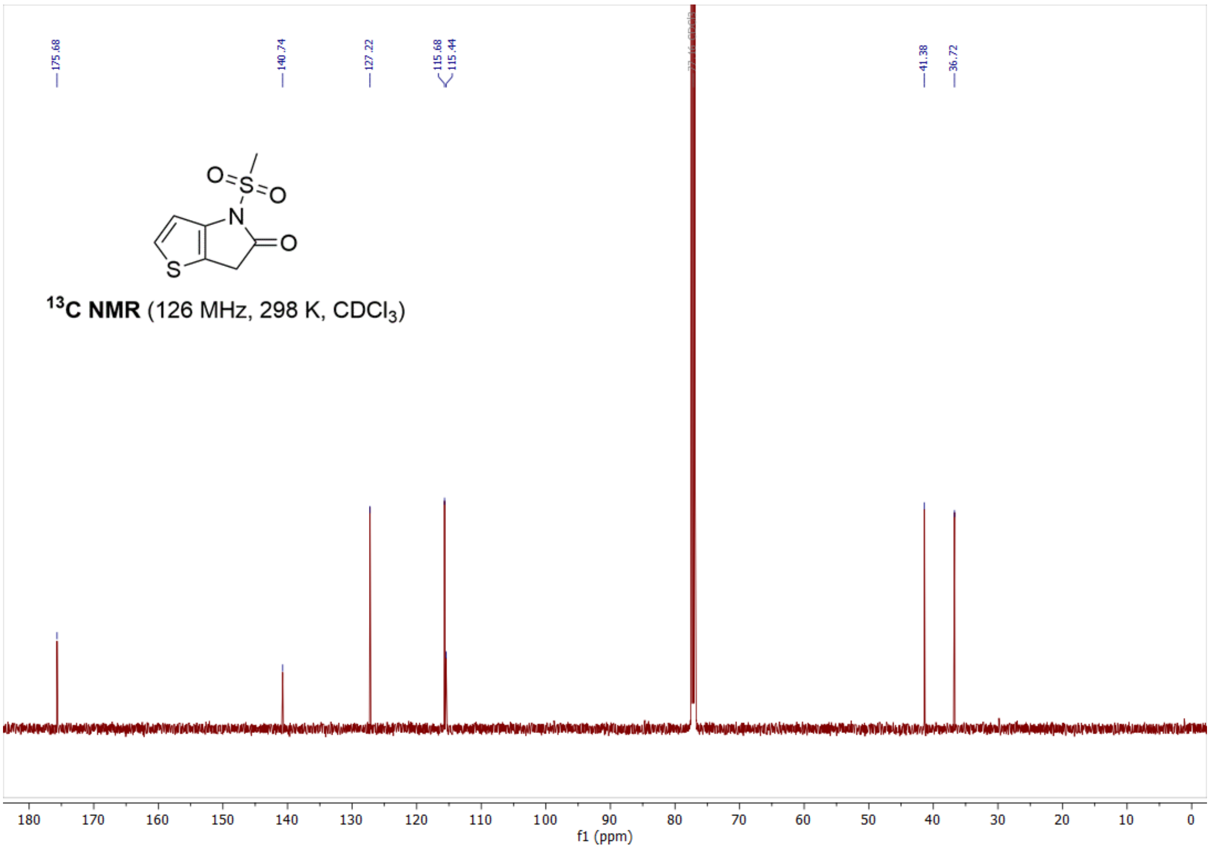

175.68
140.74
127.22
115.68
115.44
41.38
36.72
¹³C NMR (126 MHz, 298 K, CDCl₃)
180
170
160
150
140
130
120
110
100
90
80
70
60
50
40
30
20
10
0
f1 (ppm)

γ-Lactam **9**

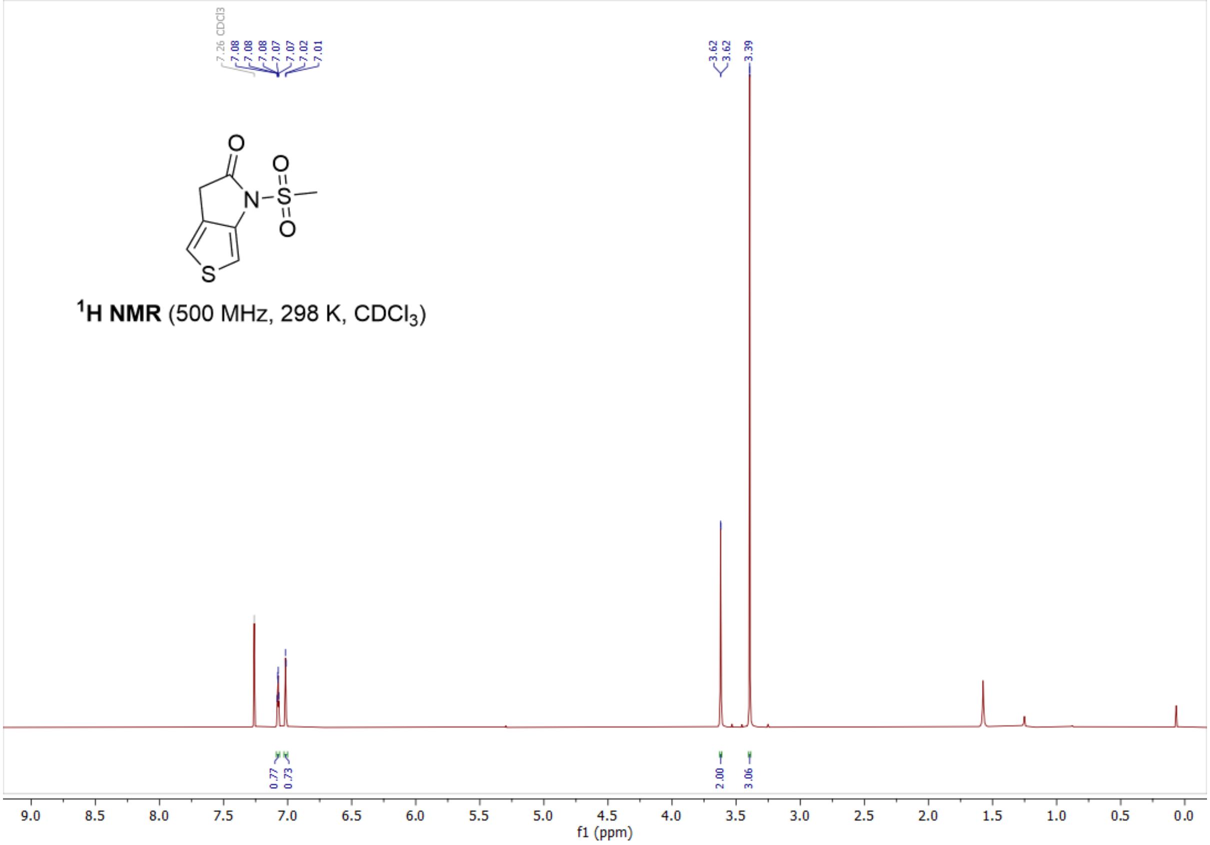

7.26 CDCl3
7.08
7.08
7.08
7.07
7.07
7.02
7.01
3.62
3.62
3.39
O
N
S
O
O
S
1H NMR (500 MHz, 298 K, CDCl3)
0.77
0.73
2.00
3.06
9.0
8.5
8.0
7.5
7.0
6.5
6.0
5.5
5.0
4.5
4.0
3.5
3.0
2.5
2.0
1.5
1.0
0.5
0.0
f1 (ppm)

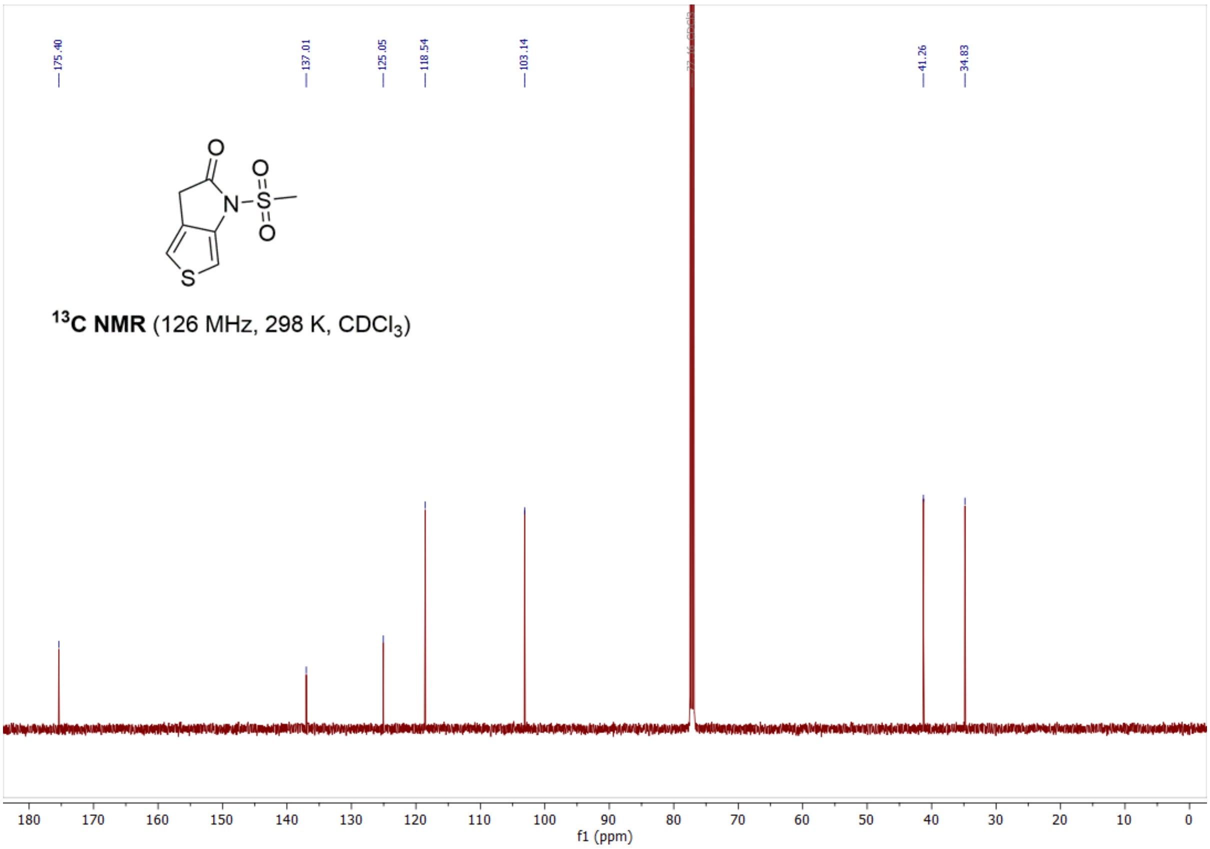

175.40
137.01
125.05
118.54
103.14
41.26
34.83
O
N
S
O
O
S
13C NMR (126 MHz, 298 K, CDCl3)
180
170
160
150
140
130
120
110
100
90
80
70
60
50
40
30
20
10
0
f1 (ppm)

Thiophene **10**

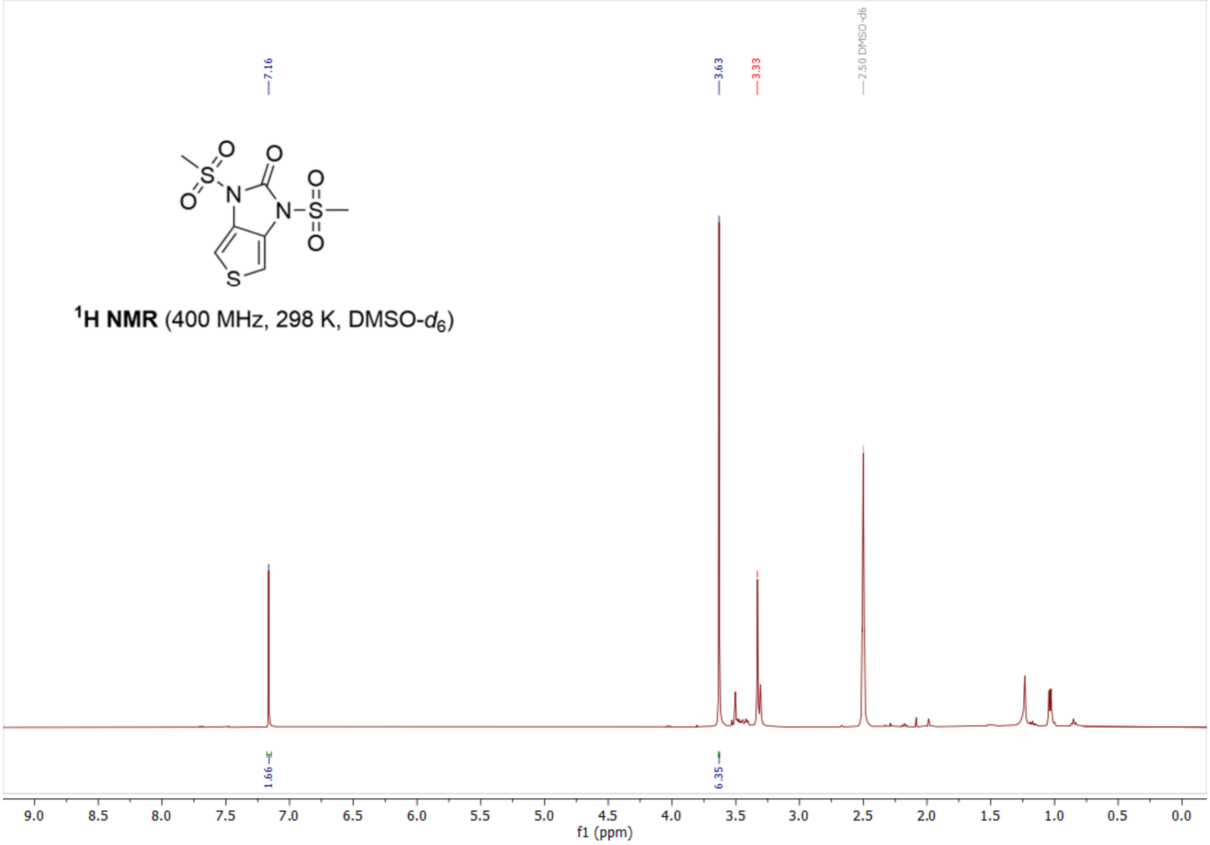

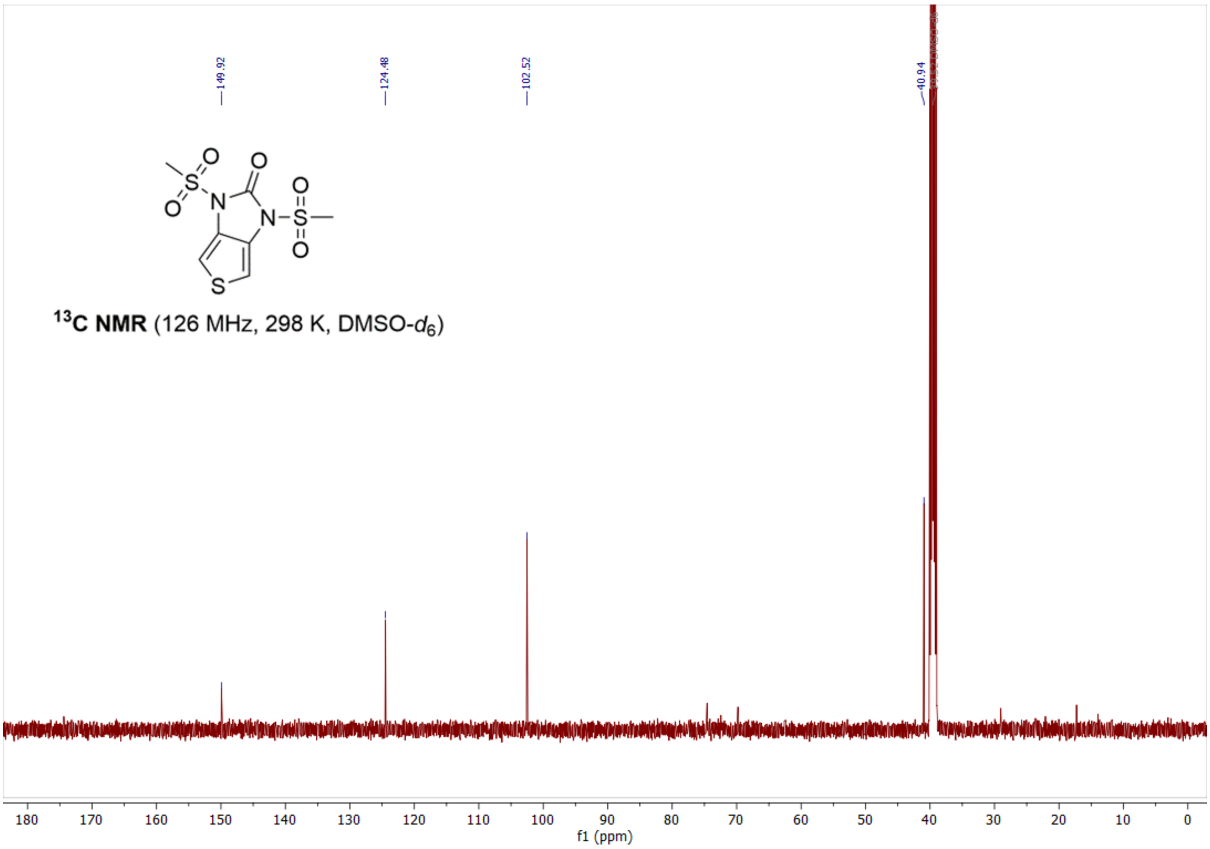

γ-Lactam **16a**

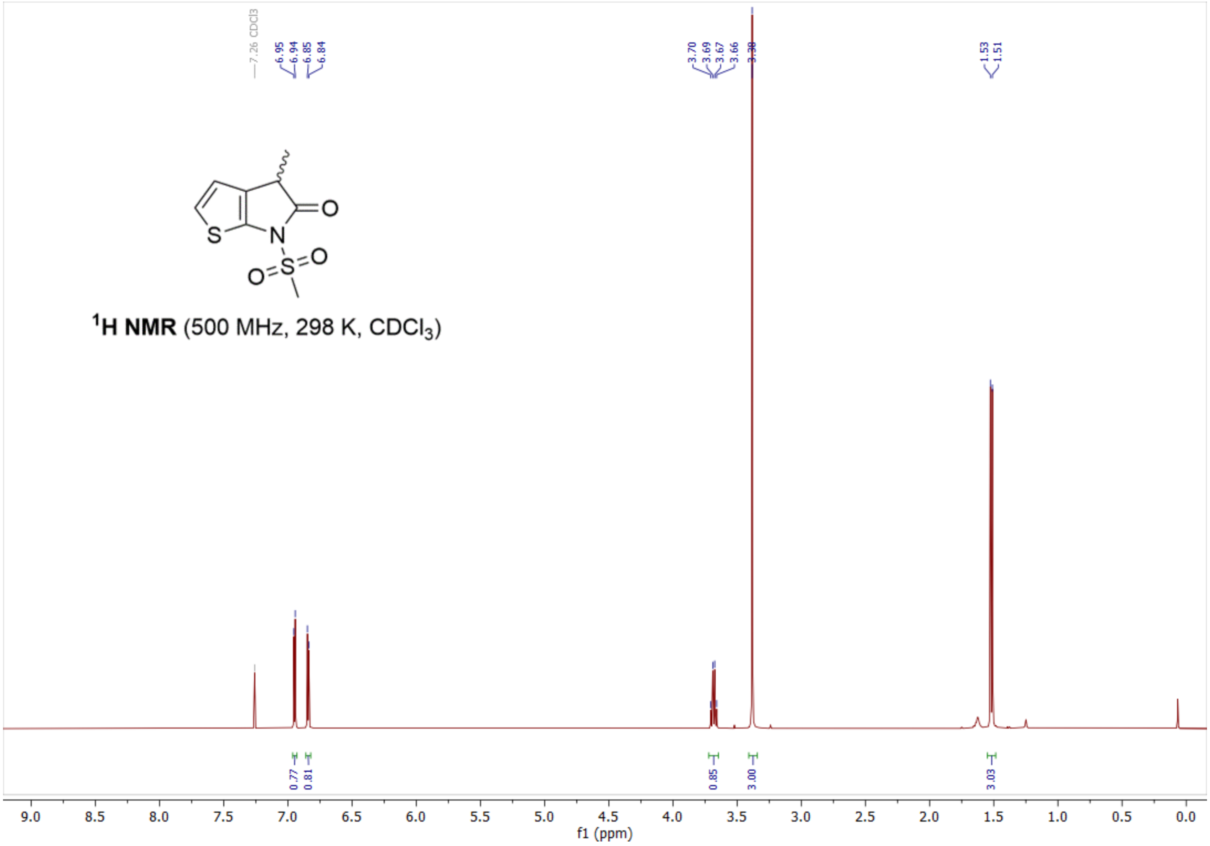

¹H NMR (500 MHz, 298 K, CDCl3)
f1 (ppm)

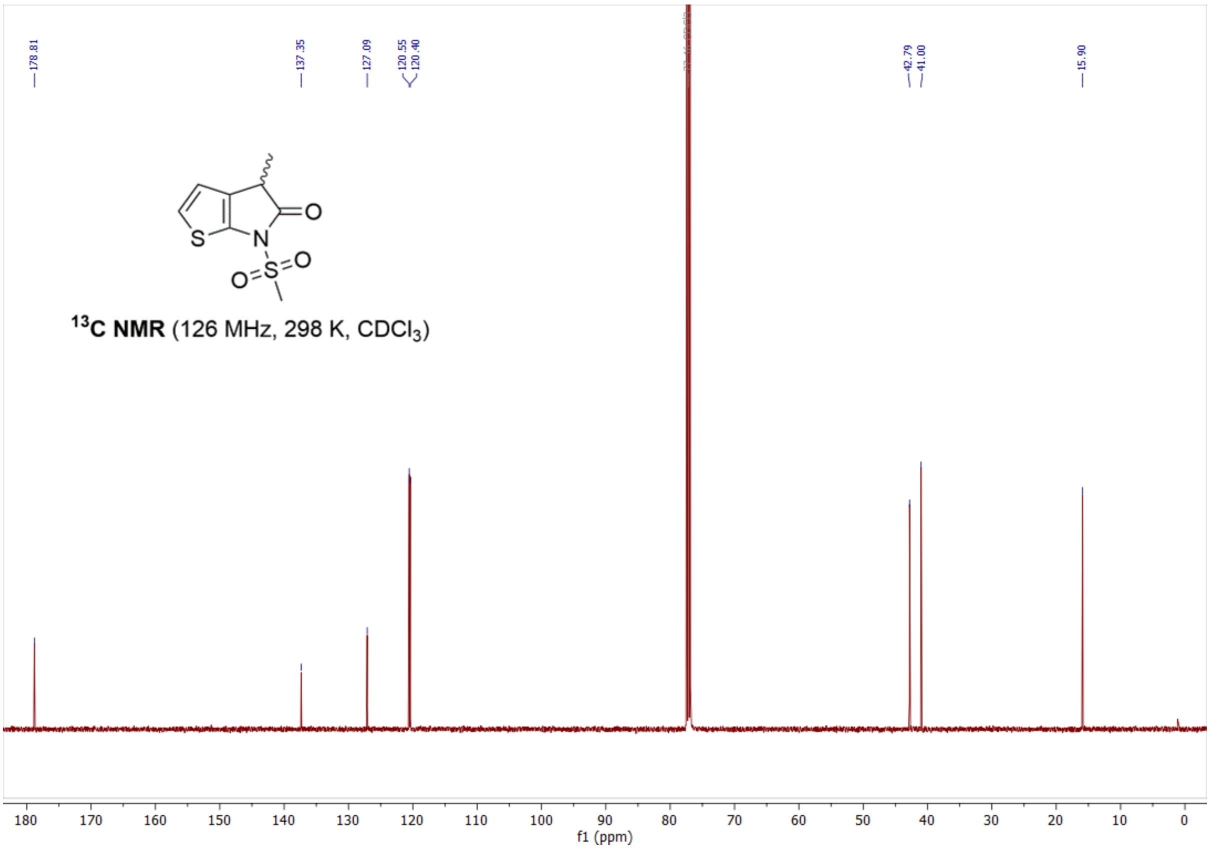

¹³C NMR (126 MHz, 298 K, CDCl3)
f1 (ppm)

γ-Lactam **16b**

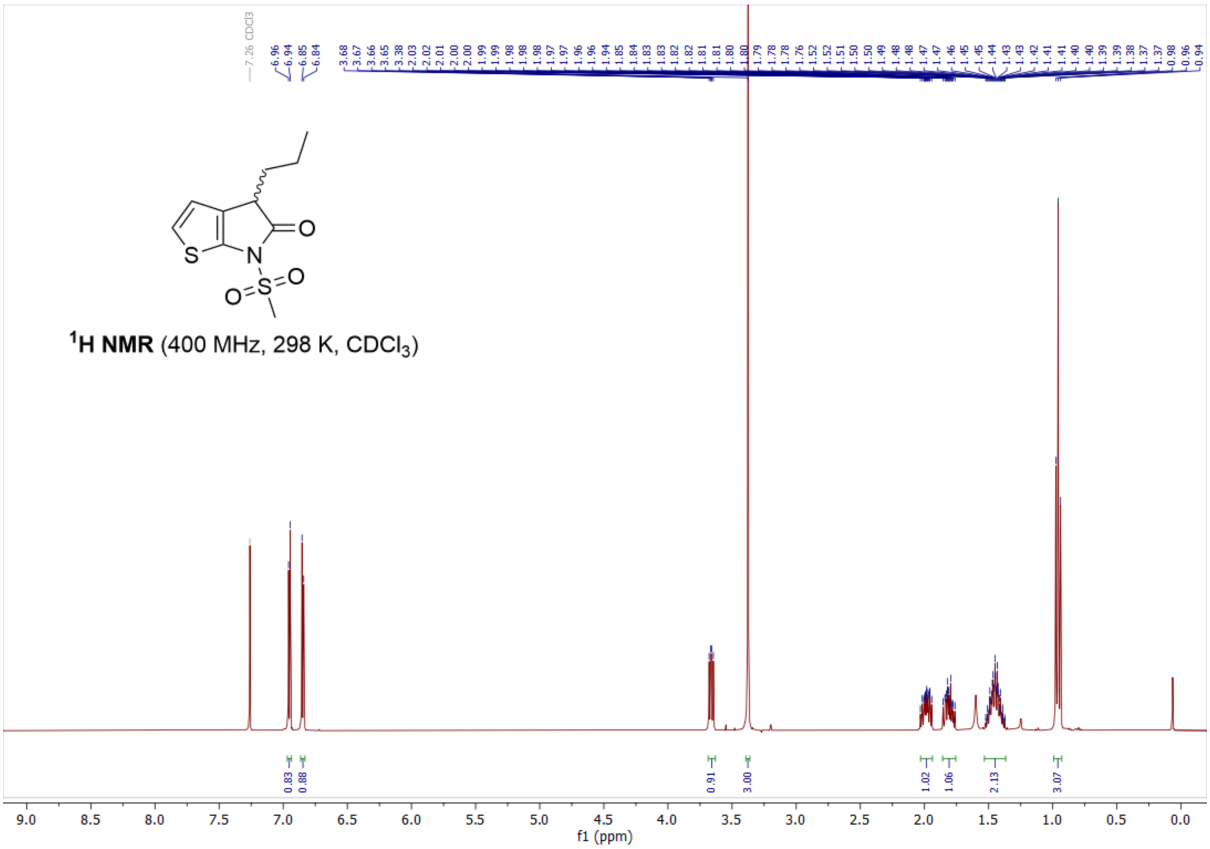

7.26 CDCl3
6.96
6.94
6.85
6.84
3.68
3.67
3.66
3.65
3.38
¹H NMR (400 MHz, 298 K, CDCl3)
0.83
0.88
0.91
3.00
1.02
1.06
2.13
3.07
f1 (ppm)

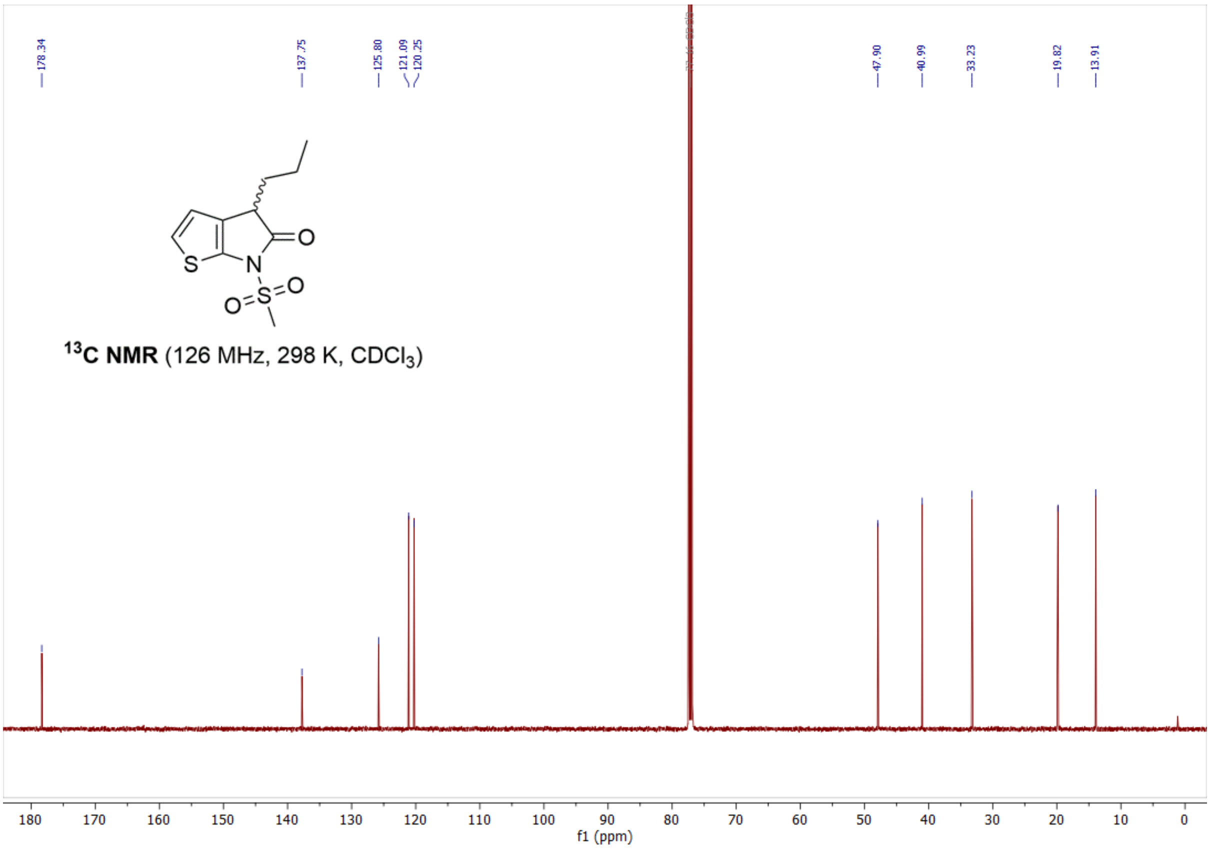

178.34
137.75
125.80
121.09
120.25
47.90
40.99
33.23
19.82
13.91
¹³C NMR (126 MHz, 298 K, CDCl3)
f1 (ppm)

γ-Lactam **16c**

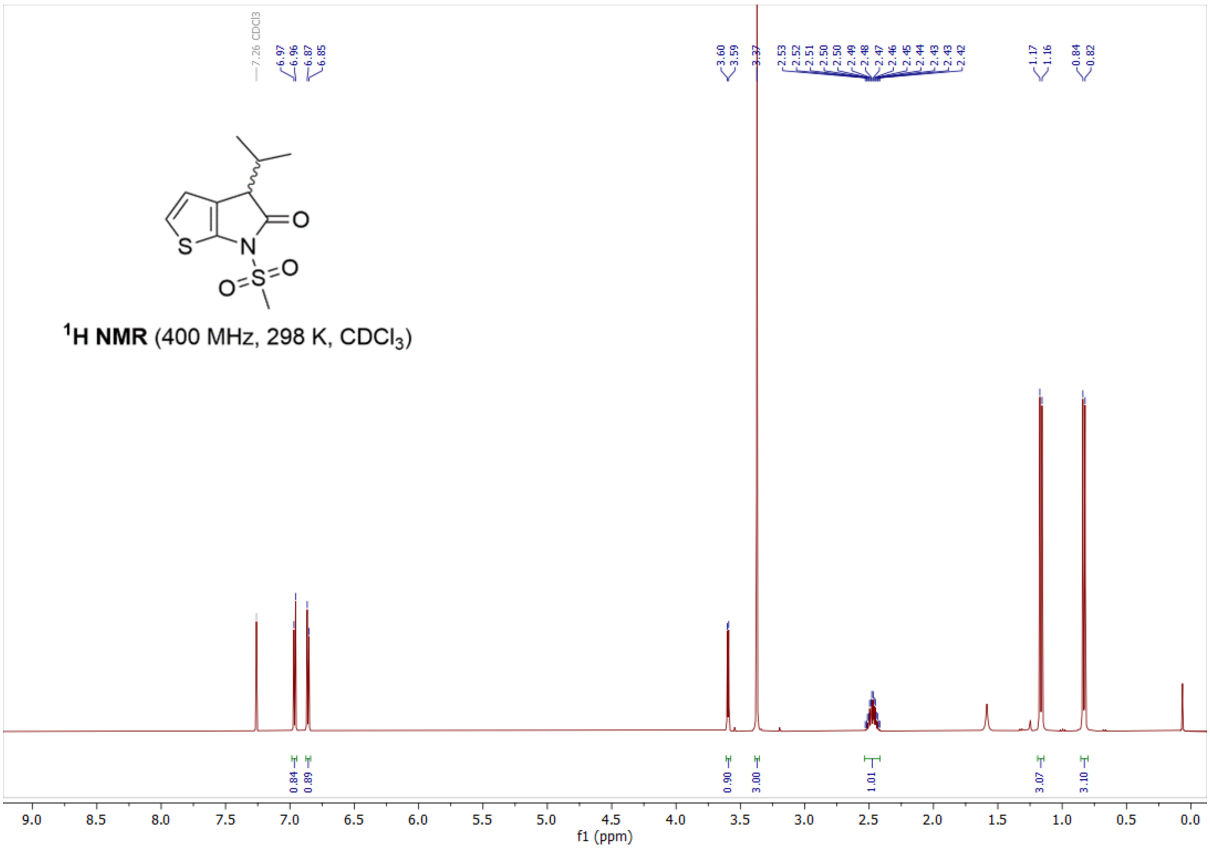

7.26 CDCl3
6.97
6.96
6.87
6.85
3.60
3.59
3.37
2.53
2.52
2.51
2.50
2.50
2.49
2.48
2.47
2.46
2.45
2.44
2.43
2.43
2.42
1.17
1.16
0.84
0.82
S
N
O
O
S
O
¹H NMR (400 MHz, 298 K, CDCl₃)
0.84
0.89
0.90
3.00
1.01
3.07
3.10
9.0
8.5
8.0
7.5
7.0
6.5
6.0
5.5
5.0
4.5
4.0
3.5
3.0
2.5
2.0
1.5
1.0
0.5
0.0
f1 (ppm)

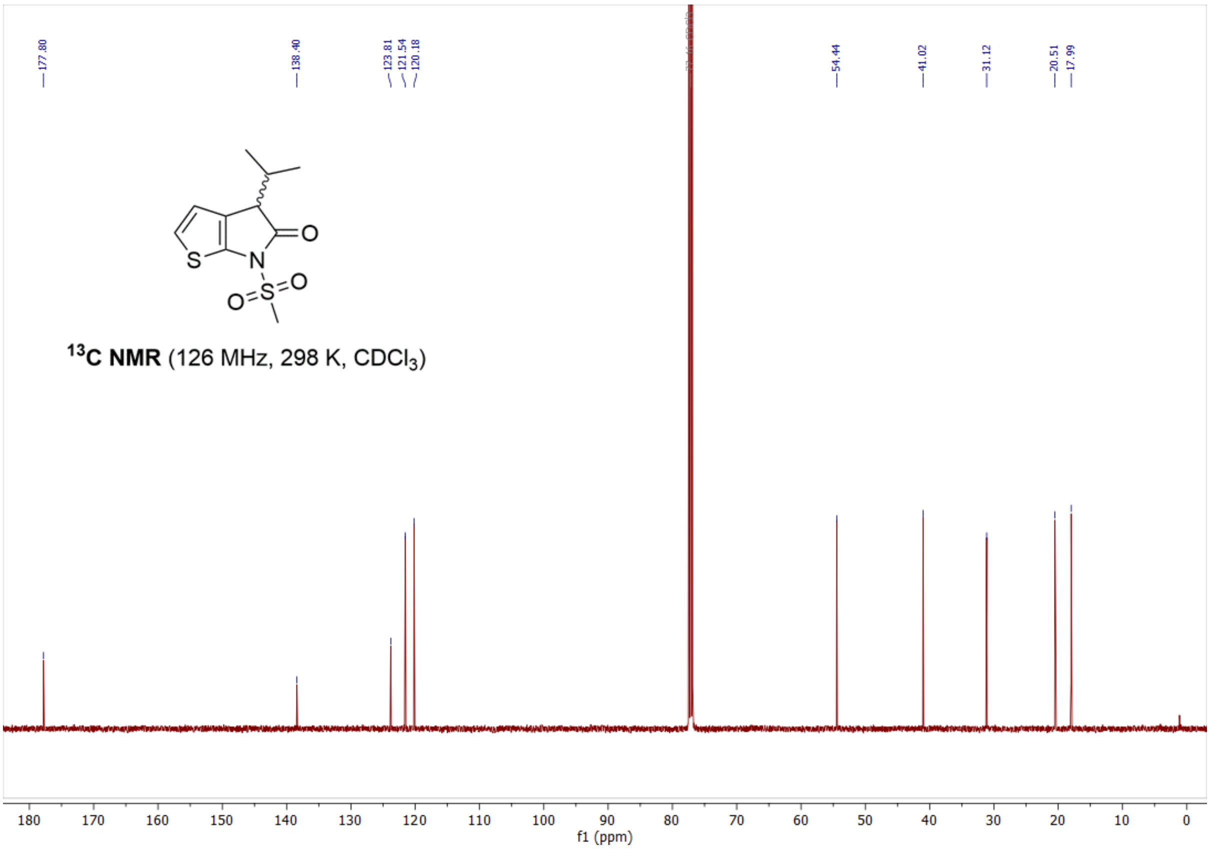

177.80
138.40
123.81
121.54
120.18
54.44
41.02
31.12
20.51
17.99
S
N
O
O
S
O
¹³C NMR (126 MHz, 298 K, CDCl₃)
180
170
160
150
140
130
120
110
100
90
80
70
60
50
40
30
20
10
0
f1 (ppm)

γ-Lactam **16d**

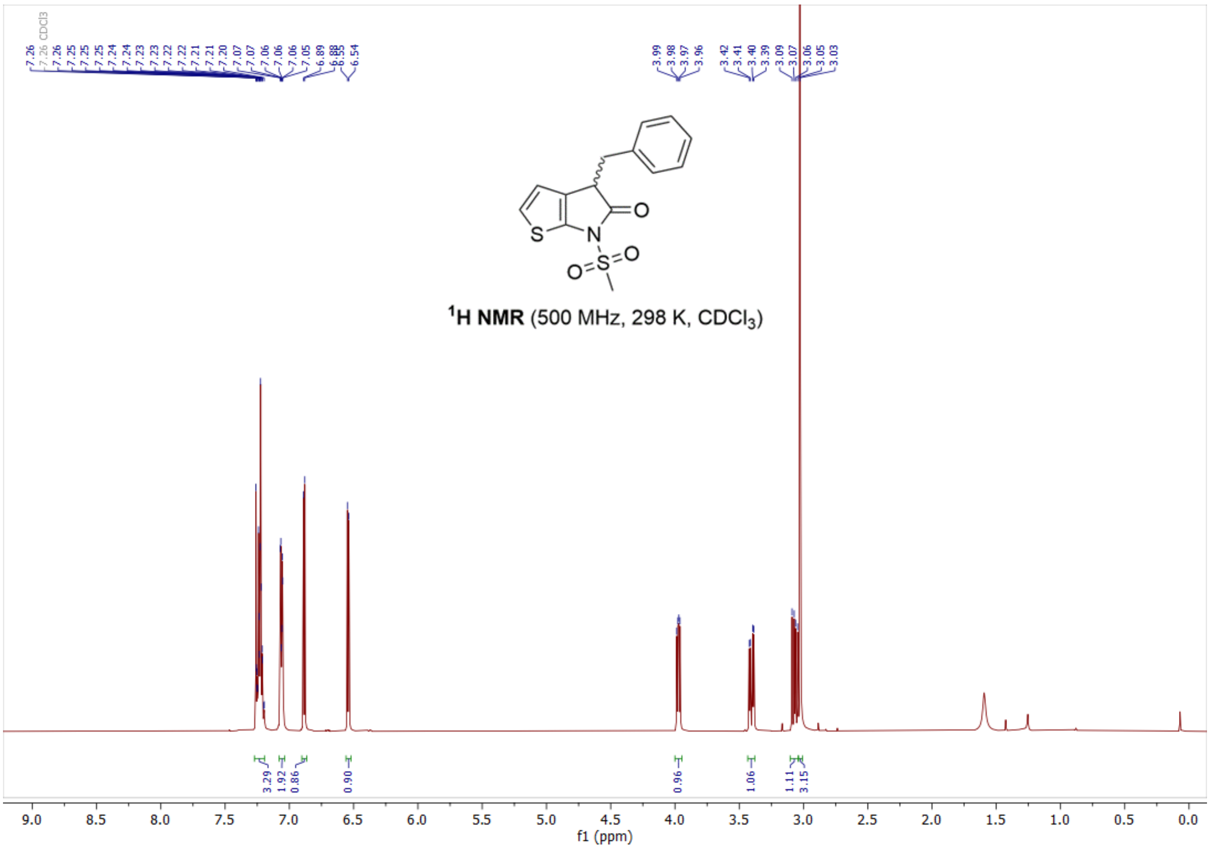

S
N
O
O=S=O
¹H NMR (500 MHz, 298 K, CDCl3)
f1 (ppm)

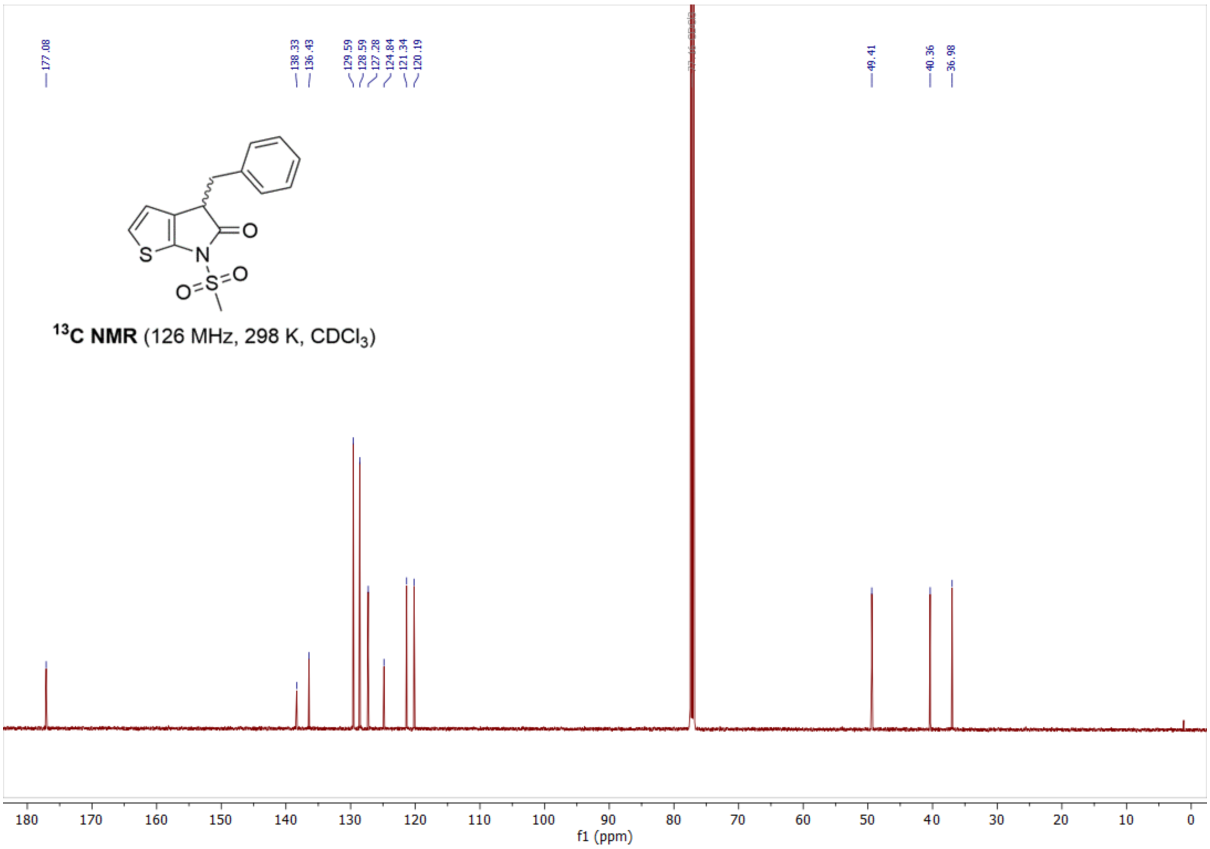

S
N
O
O=S=O
¹³C NMR (126 MHz, 298 K, CDCl3)
f1 (ppm)

γ-Lactam **16e**

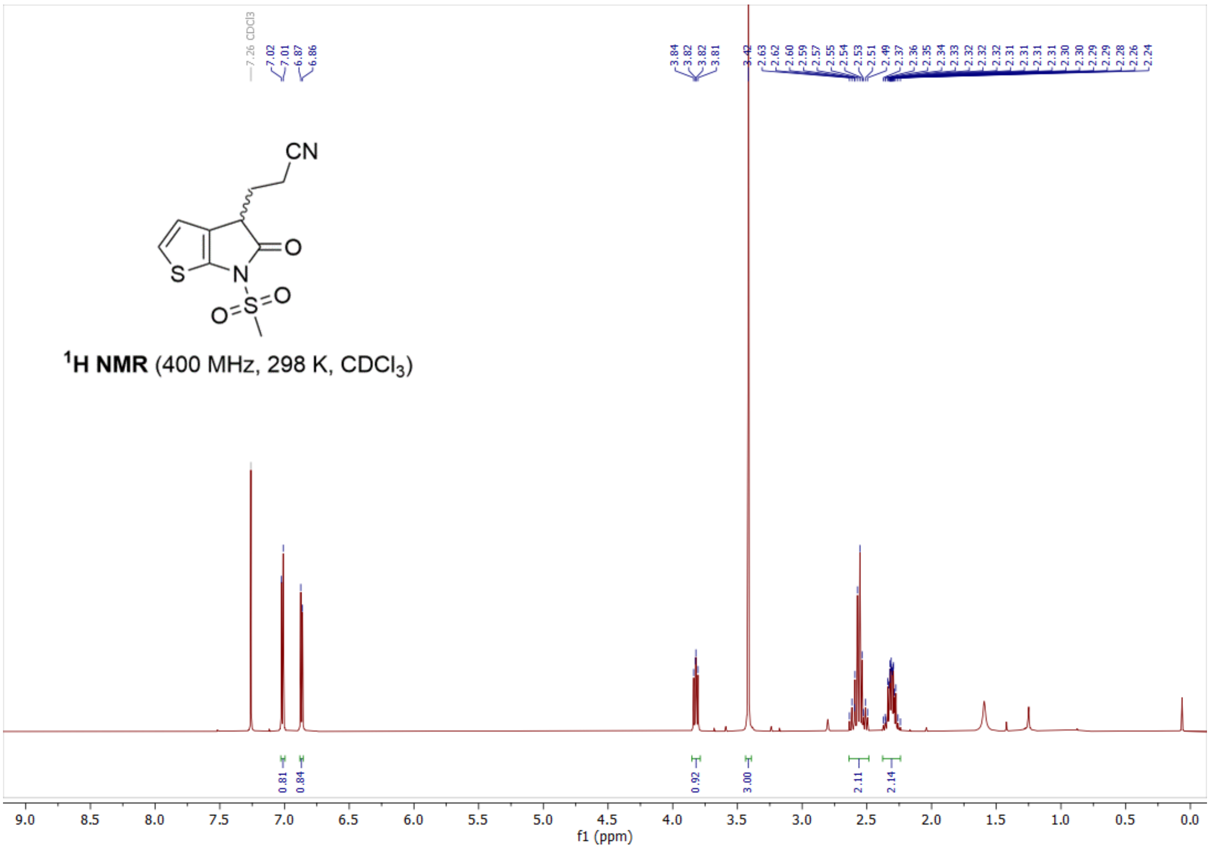

7.26 CDCl3
7.02
7.01
6.87
6.86
3.84
3.82
3.82
3.81
3.42
CN
S
N
O
O=S=O
^{1}H NMR (400 MHz, 298 K, CDCl$_3$)
0.81
0.84
0.92
3.00
2.11
2.14
f1 (ppm)

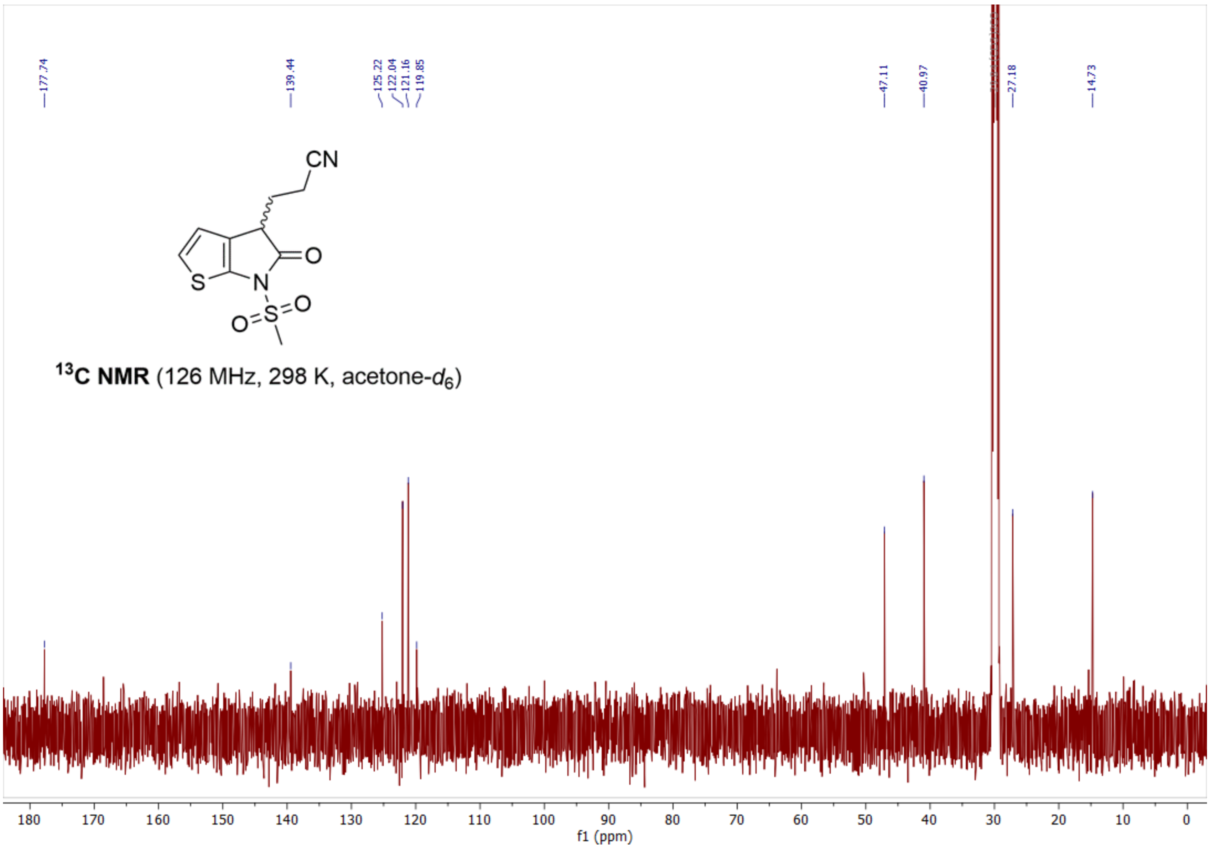

177.74
139.44
125.22
122.04
121.16
119.85
47.11
40.97
27.18
14.73
CN
S
N
O
O=S=O
^{13}C NMR (126 MHz, 298 K, acetone-d_6)
f1 (ppm)

γ-Lactam **16f**

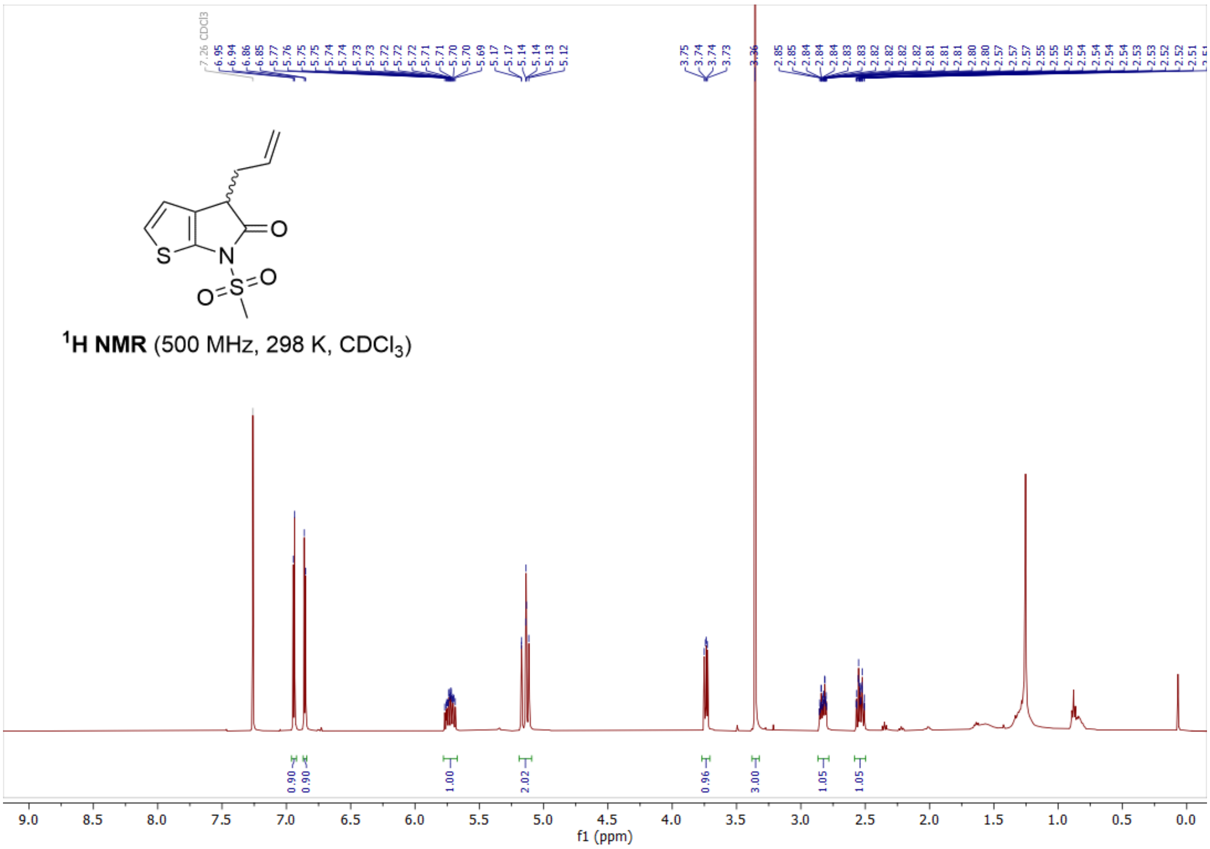

S
N
O
O=S=O
^{1}H NMR (500 MHz, 298 K, CDCl$_3$)
7.26 CDCl3
6.95
6.94
6.86
6.85
5.77
5.76
5.75
5.75
5.74
5.74
5.73
5.73
5.72
5.72
5.71
5.71
5.70
5.70
5.69
5.17
5.17
5.14
5.14
5.13
5.12
3.75
3.74
3.74
3.73
3.36
0.90
0.90
1.00
2.02
0.96
3.00
1.05
1.05
9.0
8.5
8.0
7.5
7.0
6.5
6.0
5.5
5.0
4.5
4.0
3.5
3.0
2.5
2.0
1.5
1.0
0.5
0.0
f1 (ppm)

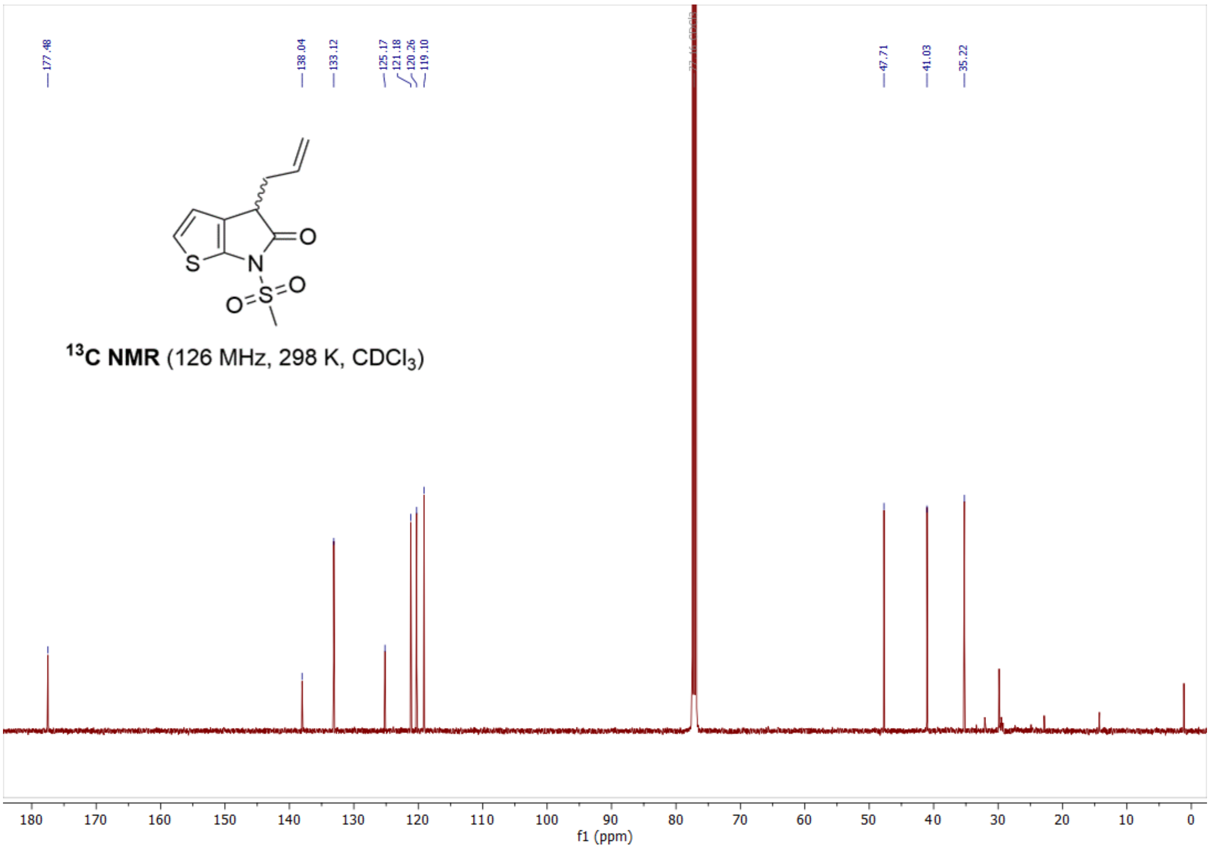

177.48
138.04
133.12
125.17
121.18
120.26
119.10
47.71
41.03
35.22
S
N
O
O=S=O
^{13}C NMR (126 MHz, 298 K, CDCl$_3$)
180
170
160
150
140
130
120
110
100
90
80
70
60
50
40
30
20
10
0
f1 (ppm)

γ-Lactam **17a**

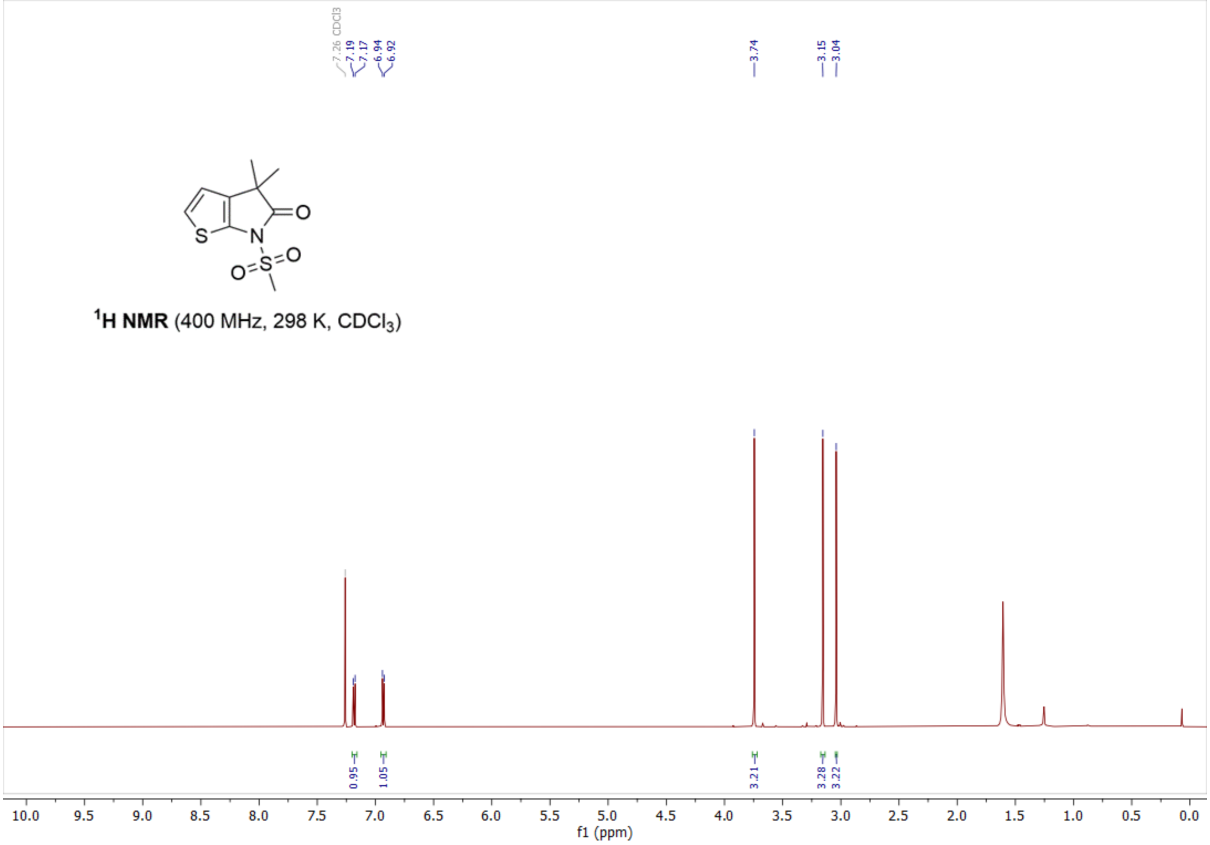
7.26 CDCl3
7.19
7.17
6.94
6.92
3.74
3.15
3.04
S
N
O
O=S=O
¹H NMR (400 MHz, 298 K, CDCl₃)
0.95
1.05
3.21
3.28
3.22
10.0 9.5 9.0 8.5 8.0 7.5 7.0 6.5 6.0 5.5 5.0 4.5 4.0 3.5 3.0 2.5 2.0 1.5 1.0 0.5 0.0
f1 (ppm)

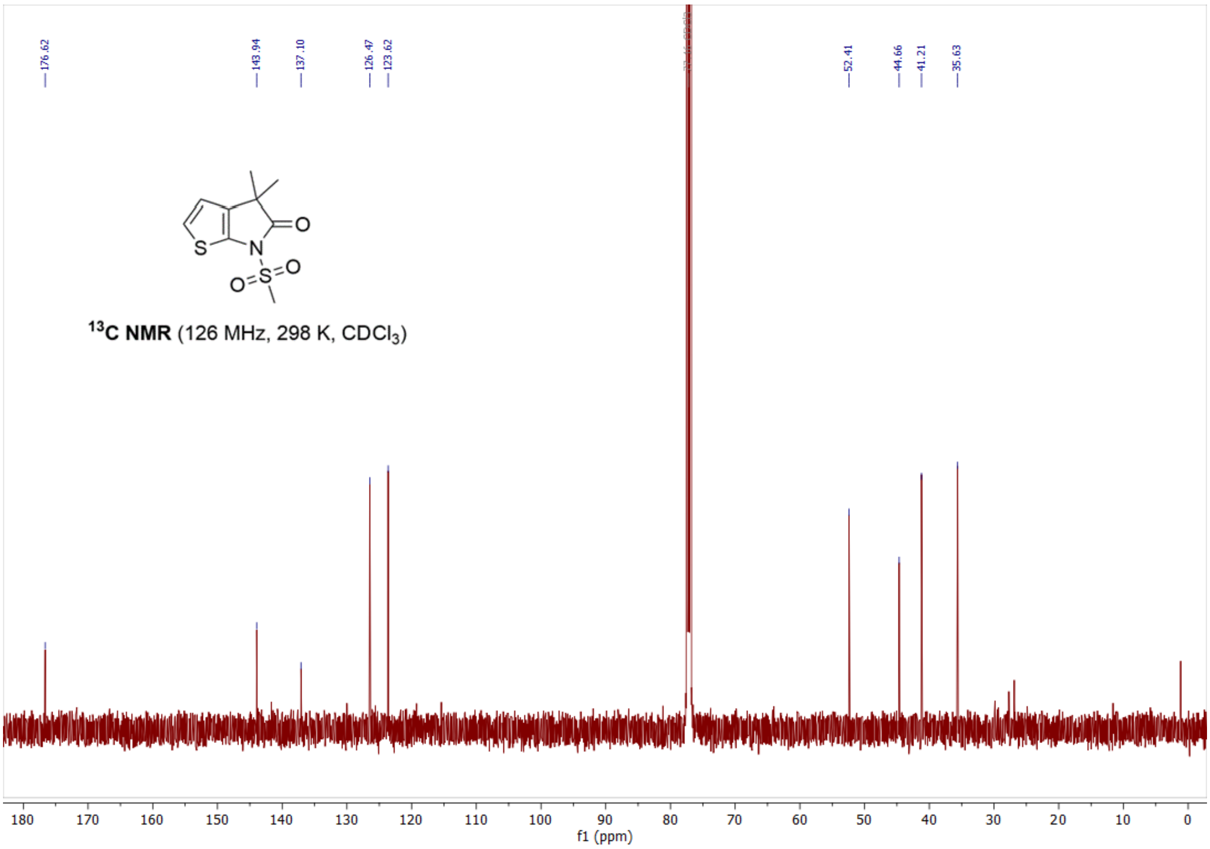
176.62
143.94
137.10
126.47
123.62
52.41
44.66
41.21
35.63
S
N
O
O=S=O
¹³C NMR (126 MHz, 298 K, CDCl₃)
180 170 160 150 140 130 120 110 100 90 80 70 60 50 40 30 20 10 0
f1 (ppm)

γ-Lactam **17b**

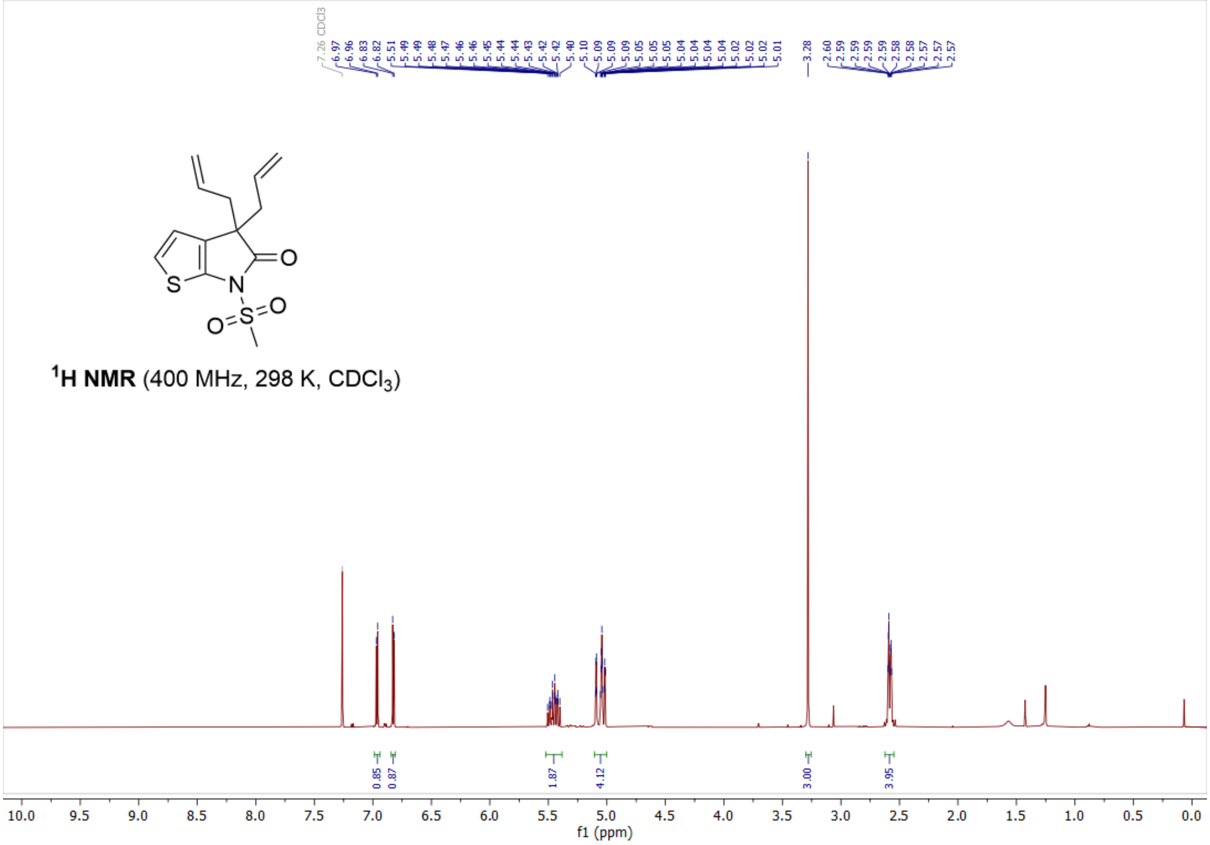

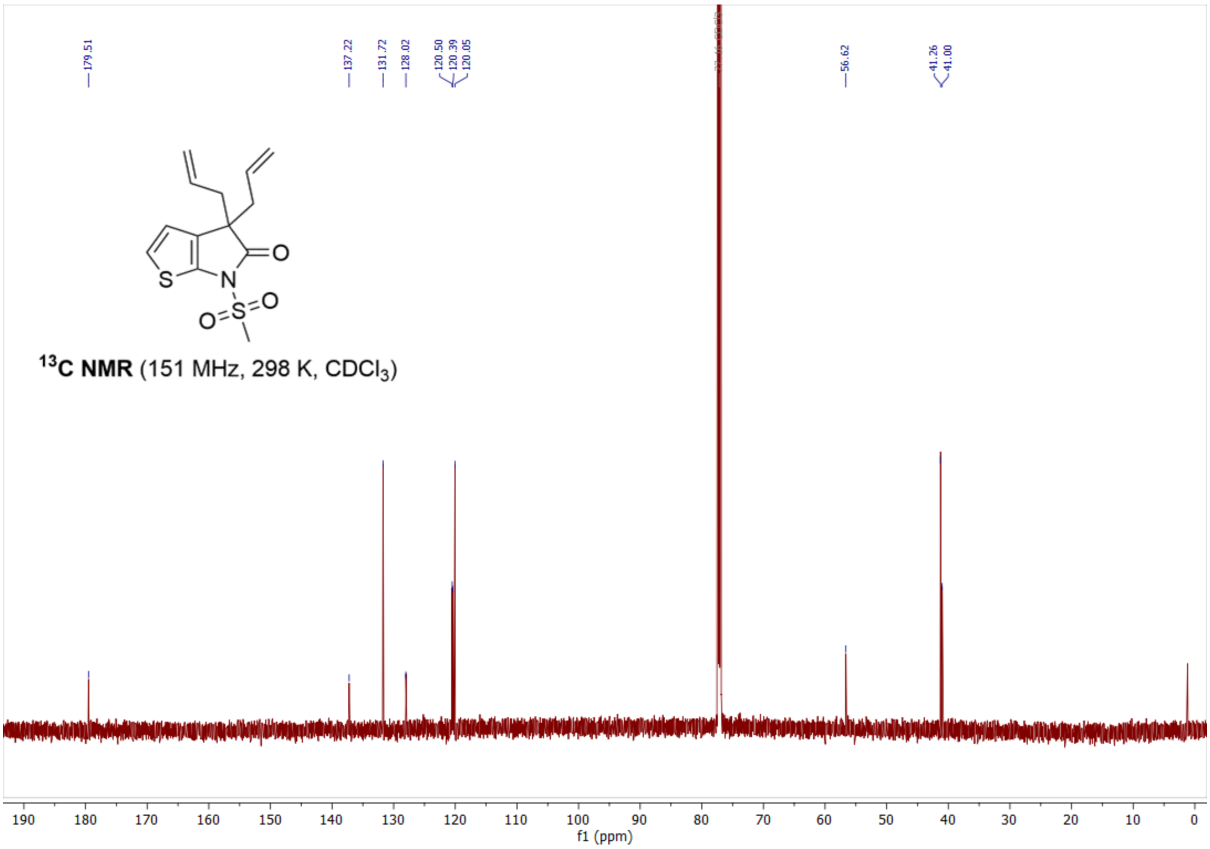

γ-Lactam **18**

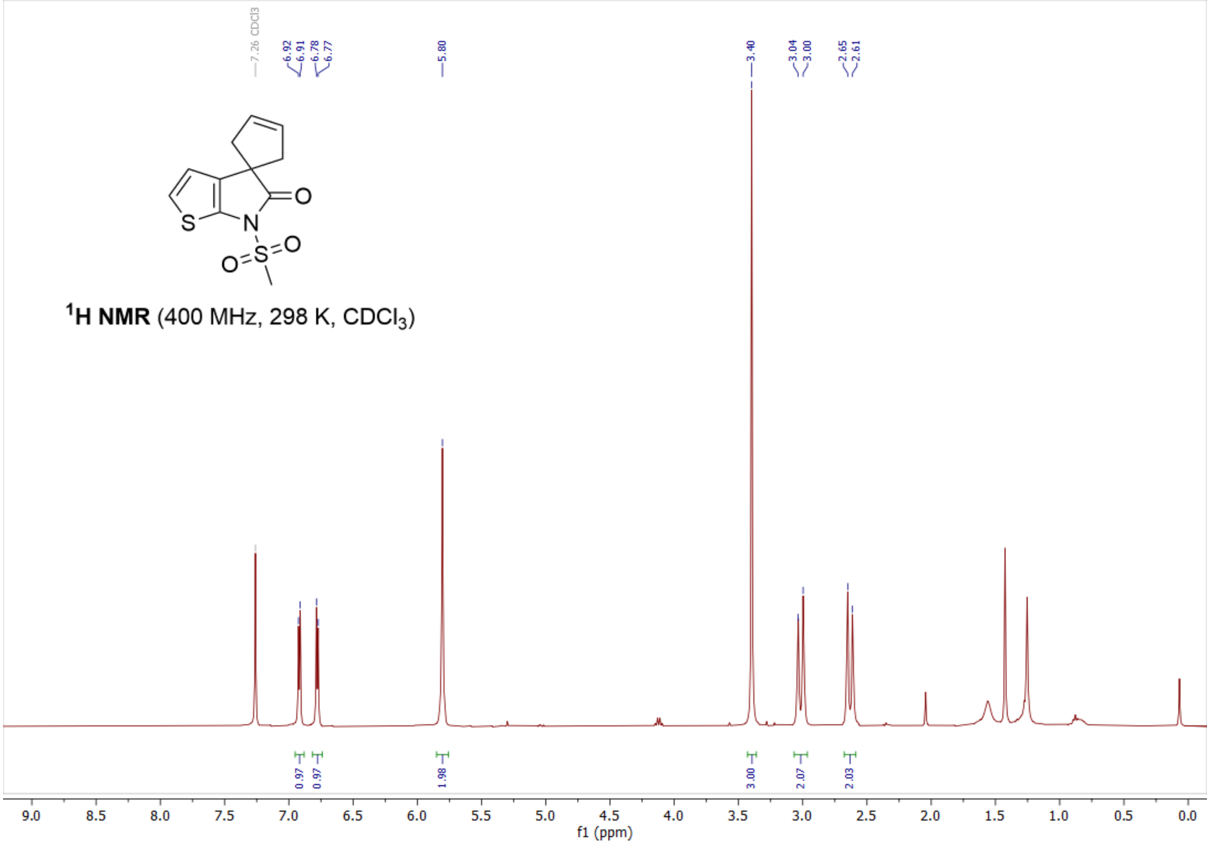

7.26 CDCl3
6.92
6.91
6.78
6.77
5.80
3.40
3.04
3.00
2.65
2.61
S
N
O
O=S=O
¹H NMR (400 MHz, 298 K, CDCl3)
0.97
0.97
1.98
3.00
2.07
2.03
9.0 8.5 8.0 7.5 7.0 6.5 6.0 5.5 5.0 4.5 4.0 3.5 3.0 2.5 2.0 1.5 1.0 0.5 0.0
f1 (ppm)

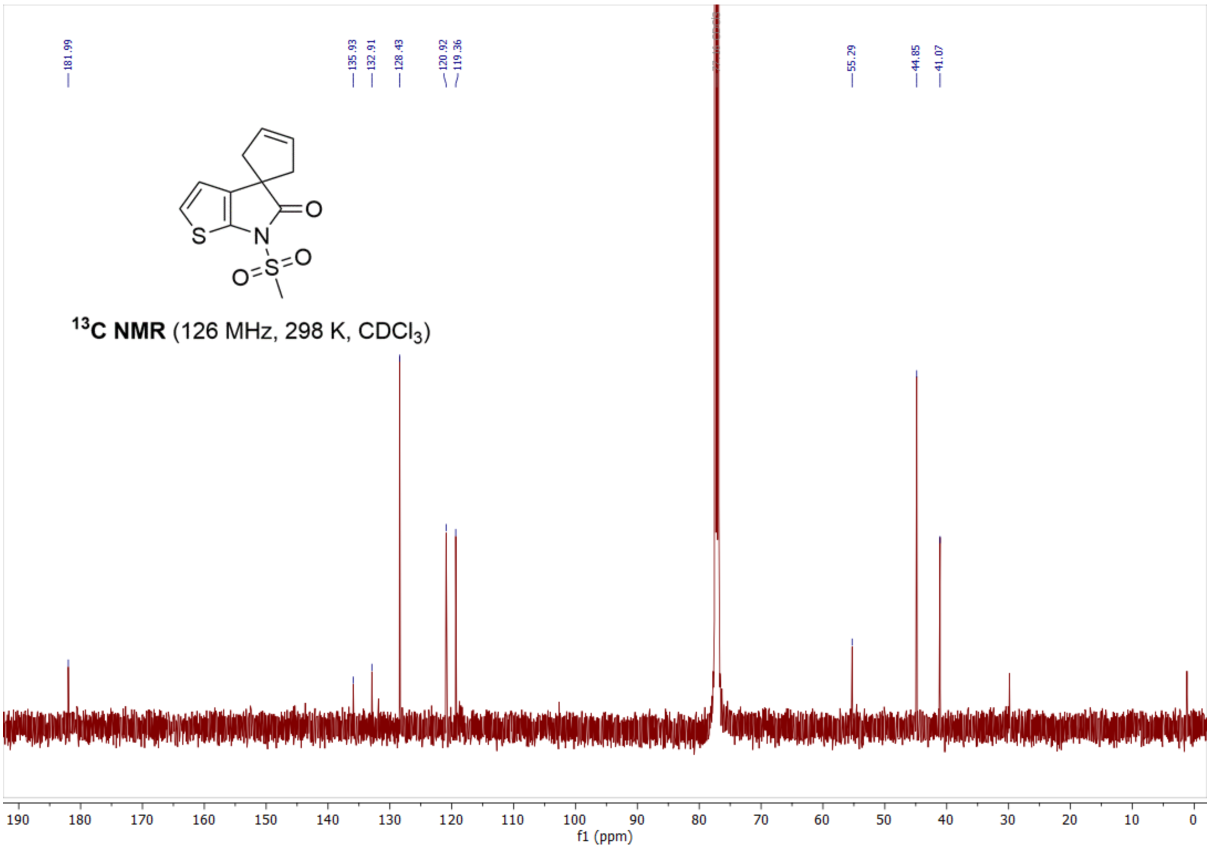

181.99
135.93
132.91
128.43
120.92
119.36
55.29
44.85
41.07
S
N
O
O=S=O
¹³C NMR (126 MHz, 298 K, CDCl3)
190 180 170 160 150 140 130 120 110 100 90 80 70 60 50 40 30 20 10 0
f1 (ppm)

γ-Lactam **19**

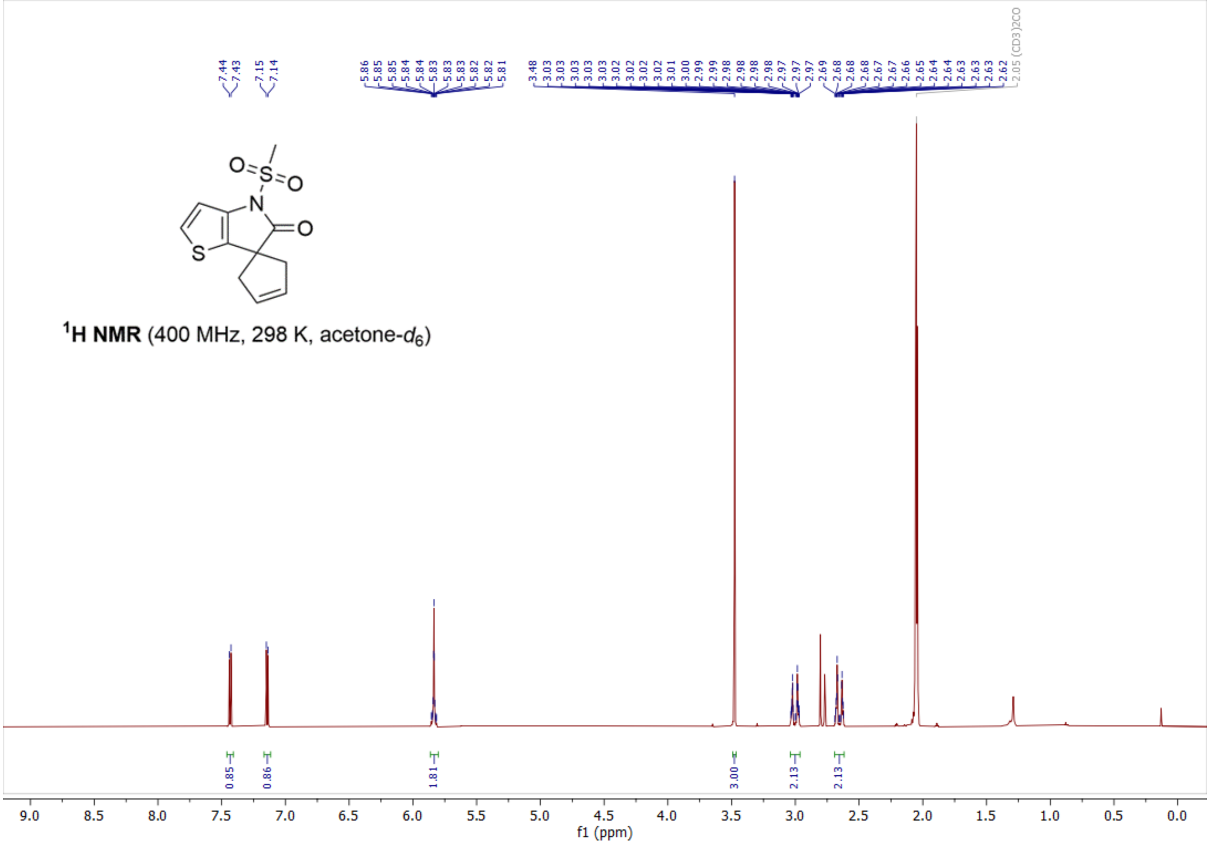

¹H NMR (400 MHz, 298 K, acetone-d6)
7.44
7.43
7.15
7.14
5.86
5.85
5.85
5.84
5.84
5.83
5.83
5.83
5.82
5.82
5.81
3.48
2.05 (CD3)2CO
0.85
0.86
1.81
3.00
2.13
2.13
f1 (ppm)

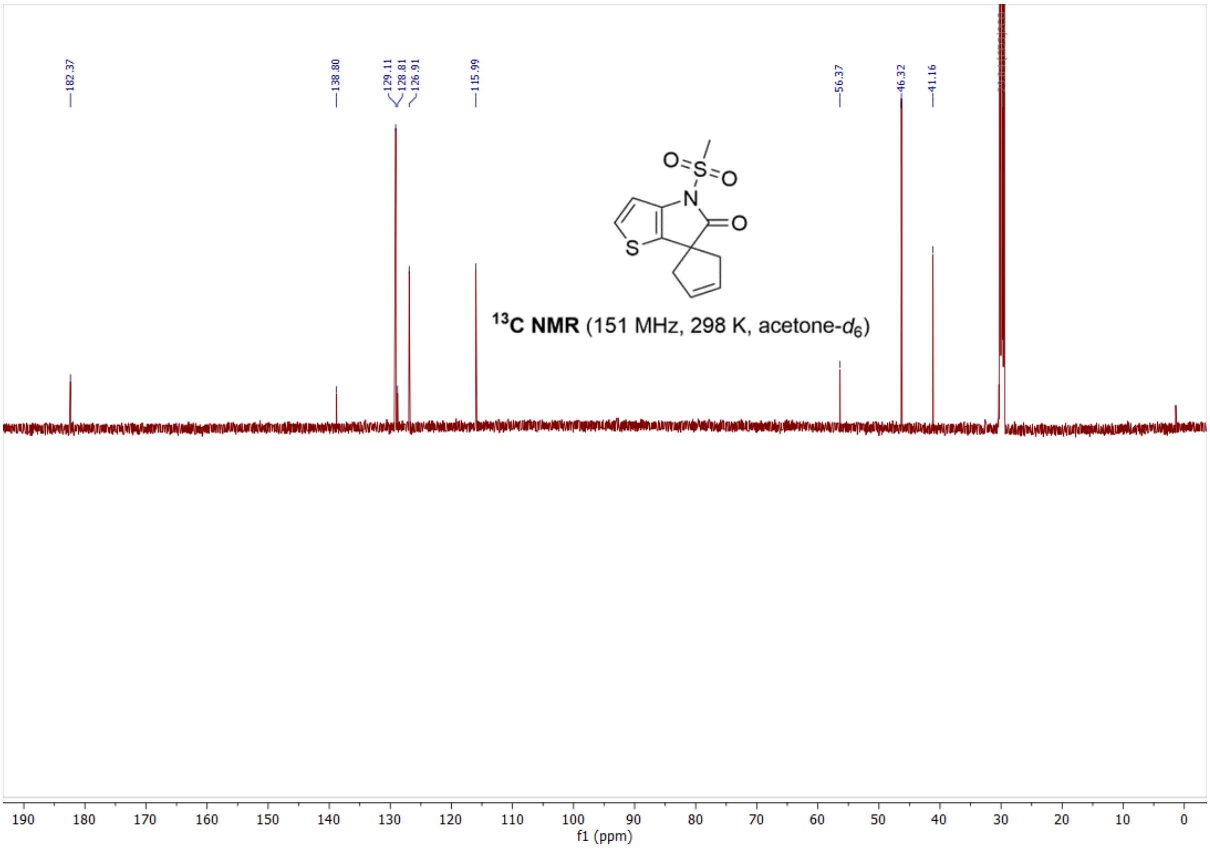

182.37
138.80
129.11
128.81
126.91
115.99
56.37
46.32
41.16
¹³C NMR (151 MHz, 298 K, acetone-d6)
f1 (ppm)

γ-Lactam **23a**

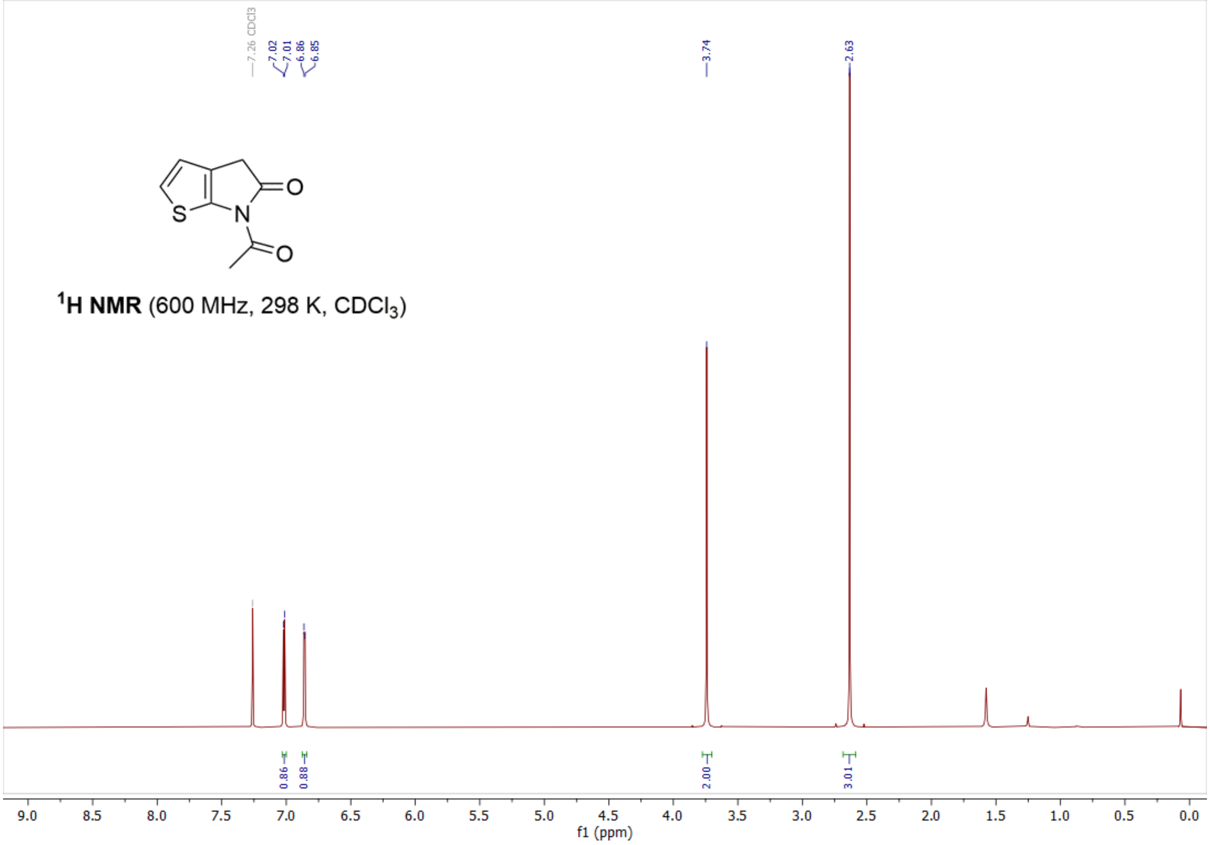

7.26 CDCl3
7.02
7.01
6.86
6.85
3.74
2.63
S
N
O
O
¹H NMR (600 MHz, 298 K, CDCl3)
0.86
0.88
2.00
3.01
9.0 8.5 8.0 7.5 7.0 6.5 6.0 5.5 5.0 4.5 4.0 3.5 3.0 2.5 2.0 1.5 1.0 0.5 0.0
f1 (ppm)

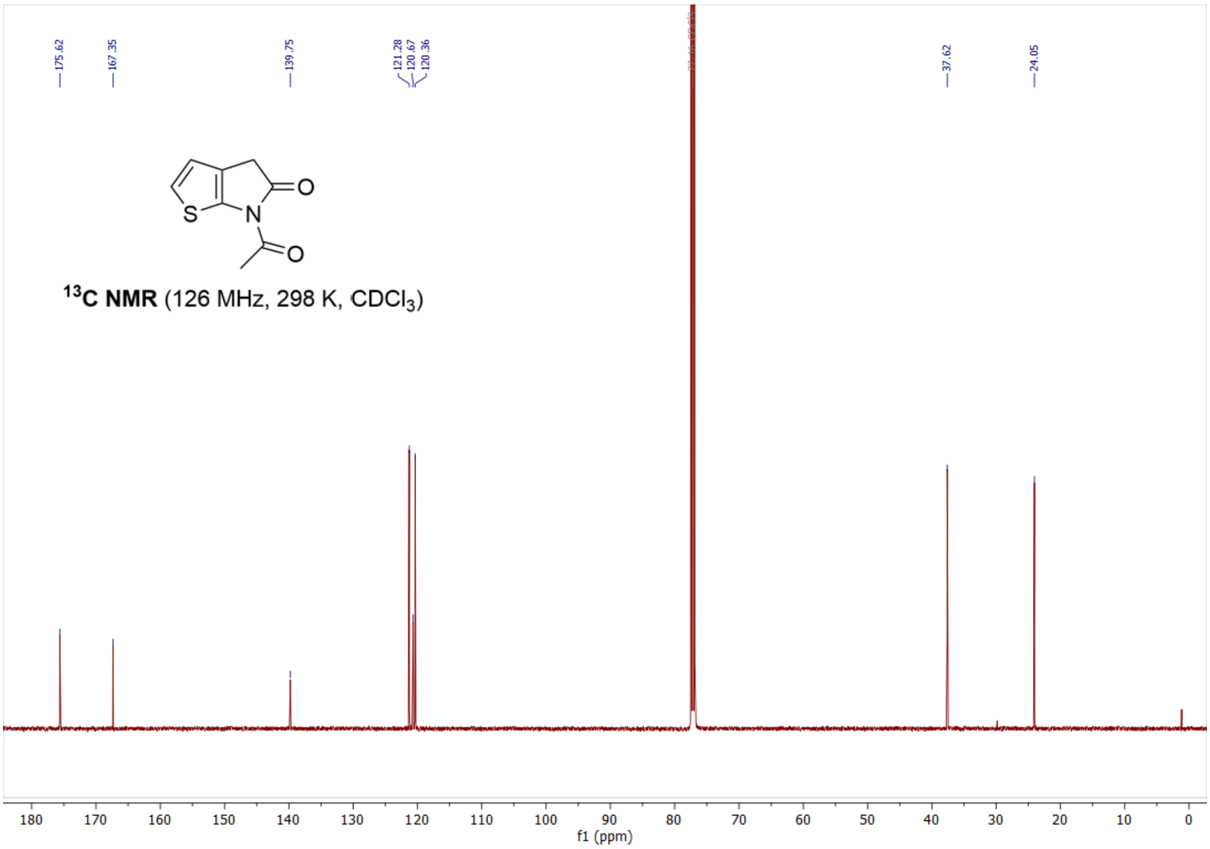

175.62
167.35
139.75
121.28
120.67
120.36
37.62
24.05
S
N
O
O
¹³C NMR (126 MHz, 298 K, CDCl3)
180 170 160 150 140 130 120 110 100 90 80 70 60 50 40 30 20 10 0
f1 (ppm)

γ-Lactam **23b**

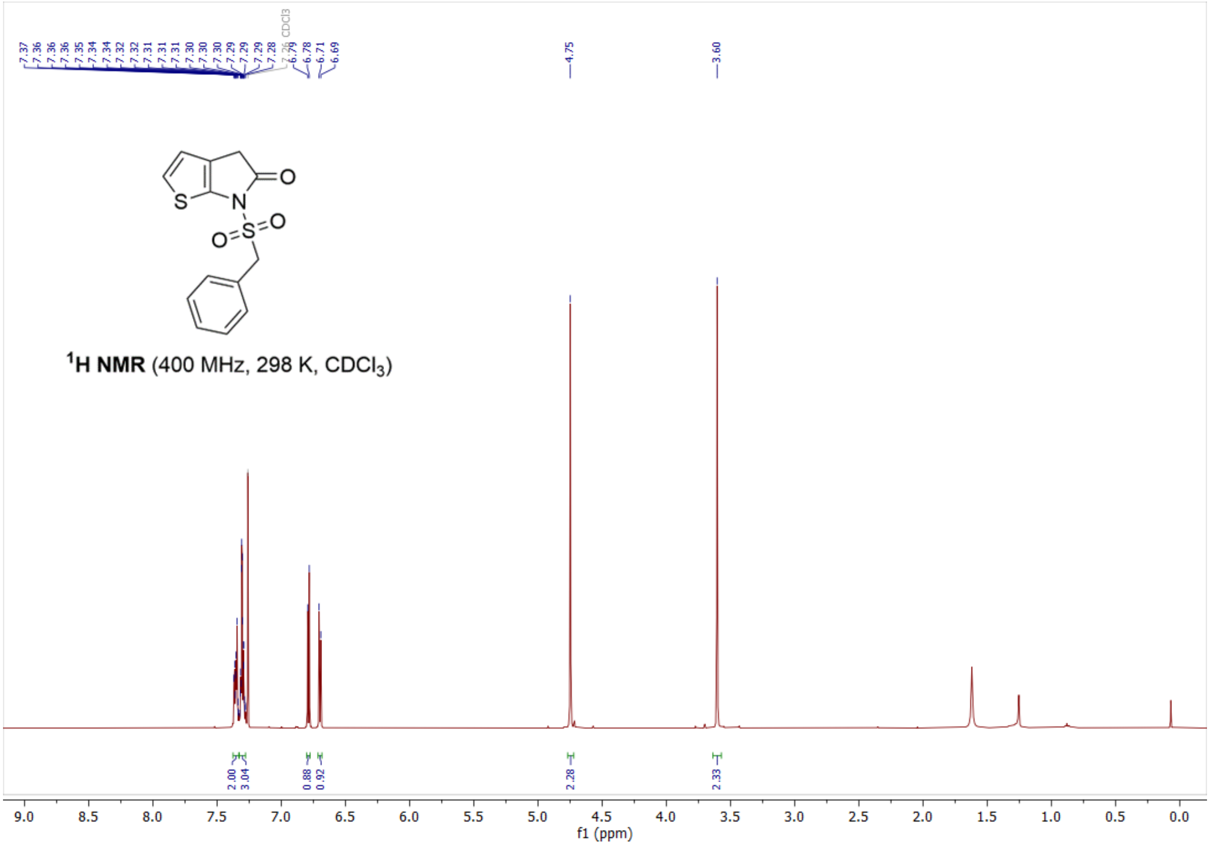

7.37
7.36
7.36
7.36
7.35
7.34
7.34
7.32
7.32
7.31
7.31
7.31
7.30
7.30
7.30
7.29
7.29
7.29
7.28
7.26 CDCl3
6.79
6.78
6.71
6.69
4.75
3.60
^{1}H NMR (400 MHz, 298 K, CDCl$_3$)
2.00
3.04
0.88
0.92
2.28
2.33
f1 (ppm)

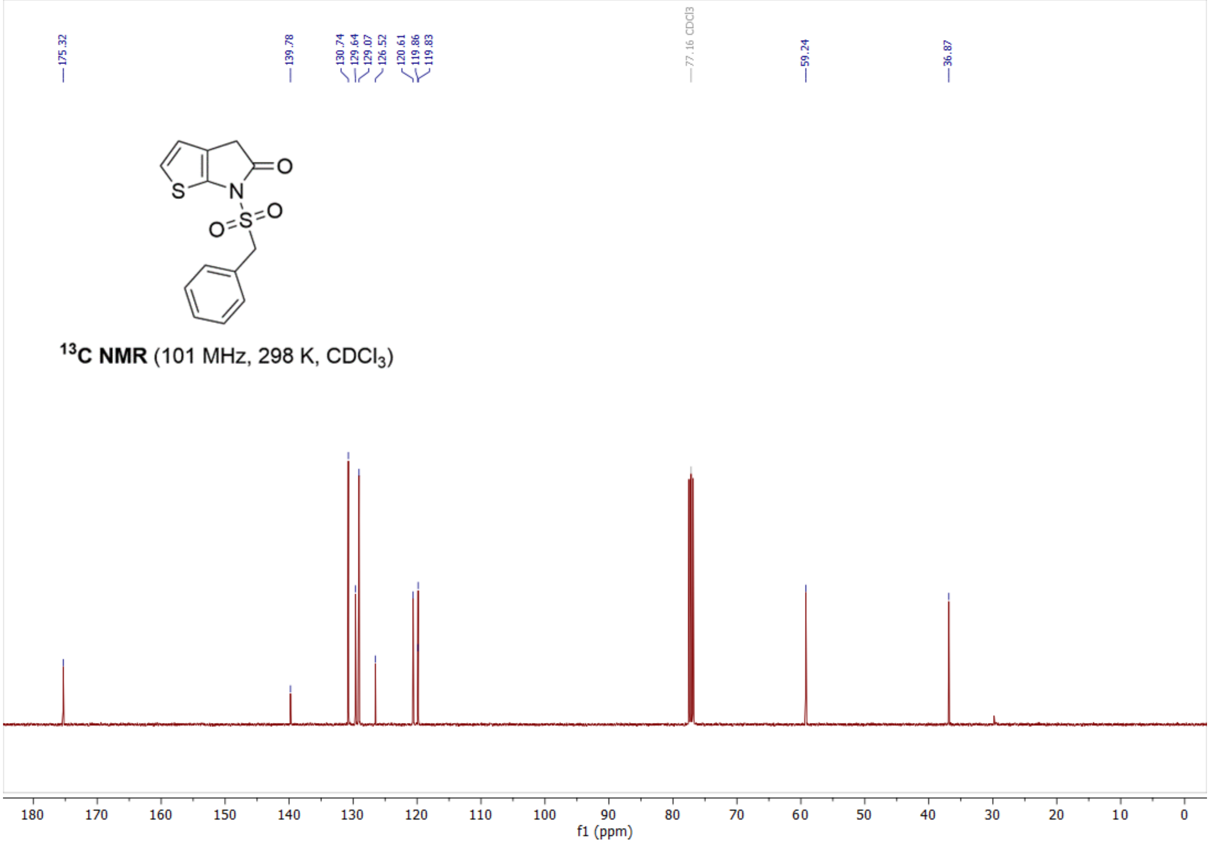

175.32
139.78
130.74
129.64
129.07
126.52
120.61
119.86
119.83
77.16 CDCl3
59.24
36.87
^{13}C NMR (101 MHz, 298 K, CDCl$_3$)
f1 (ppm)

γ-Lactam **23c**

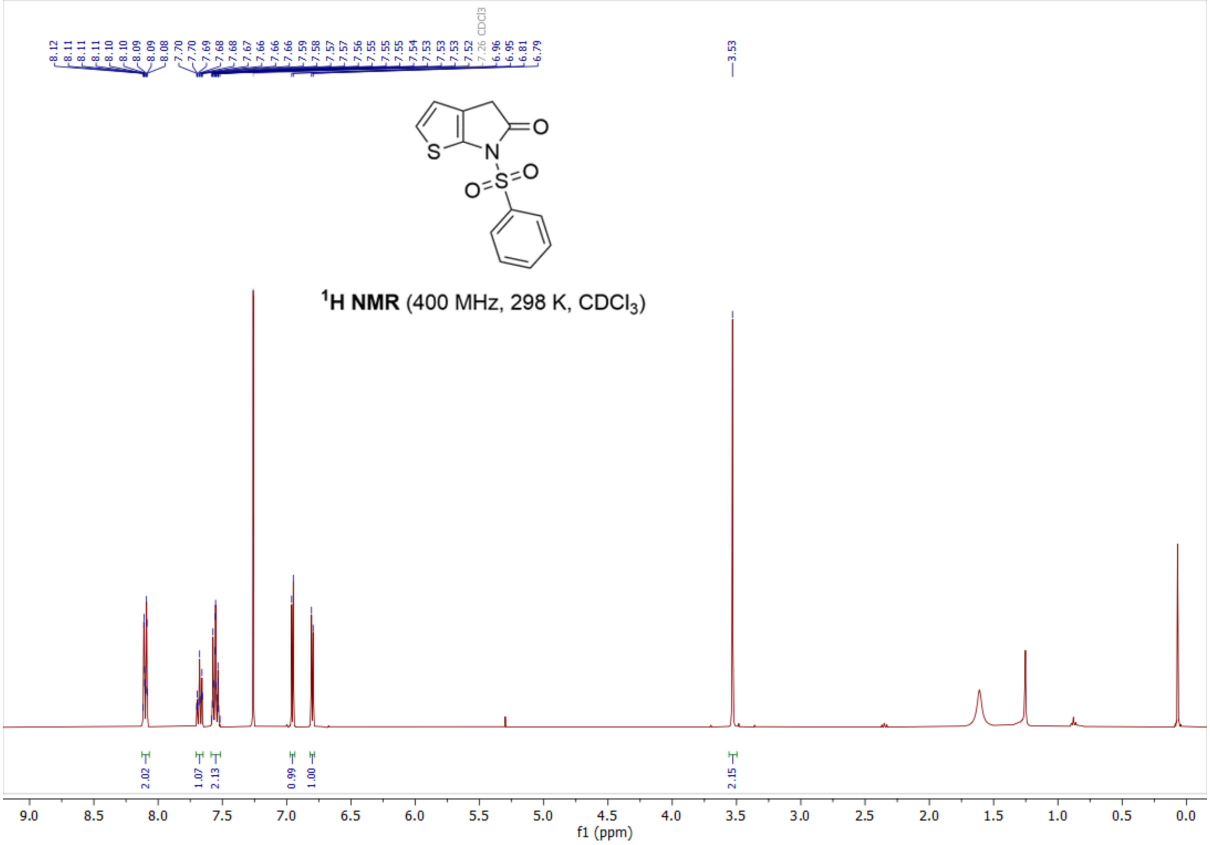

8.12 8.11 8.11 8.11 8.10 8.09 8.09 8.08 7.70 7.70 7.69 7.68 7.68 7.67 7.66 7.66 7.66 7.59 7.58 7.57 7.57 7.56 7.55 7.55 7.55 7.54 7.53 7.53 7.53 7.52 7.26 CDCl3 6.96 6.95 6.81 6.79
3.53
S
N
O
O=S=O
^{1}H NMR (400 MHz, 298 K, $CDCl_3$)
2.02 1.07 2.13 0.99 1.00 2.15
9.0 8.5 8.0 7.5 7.0 6.5 6.0 5.5 5.0 4.5 4.0 3.5 3.0 2.5 2.0 1.5 1.0 0.5 0.0
f1 (ppm)

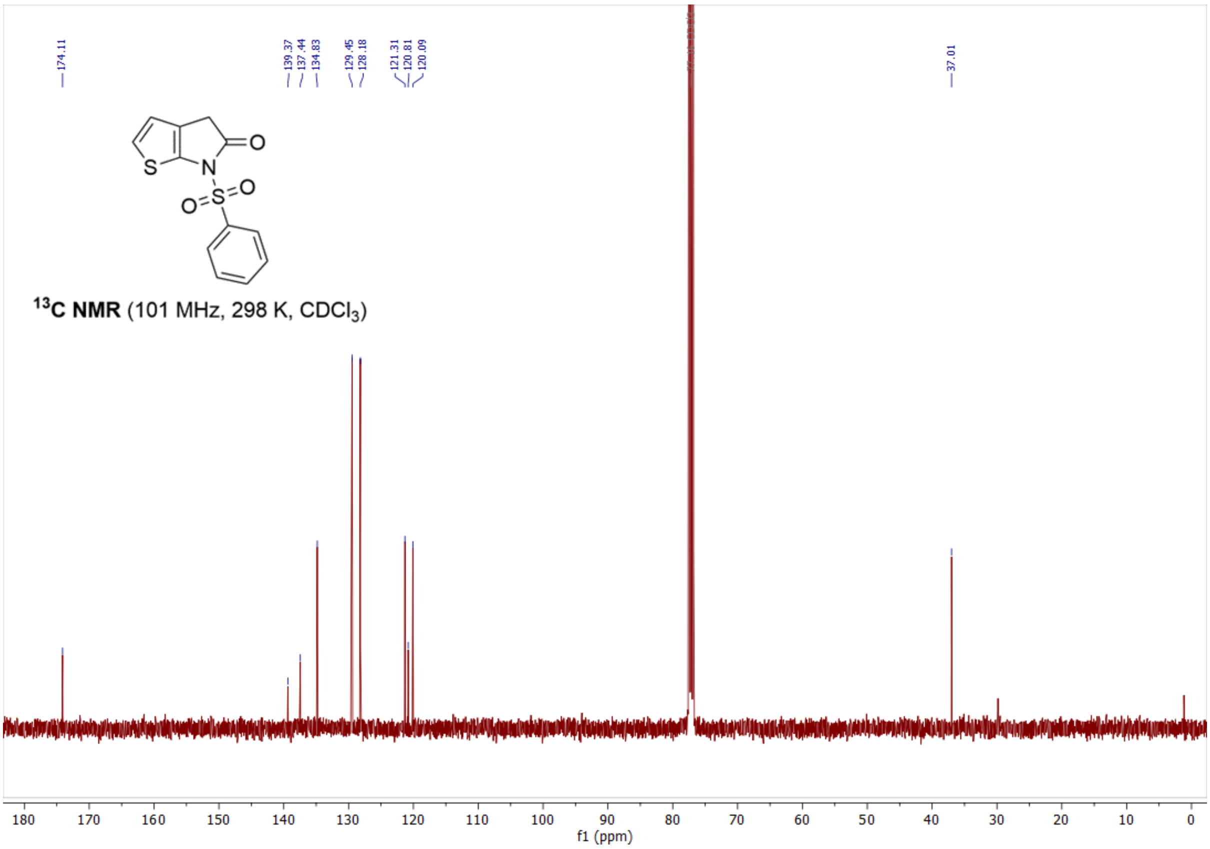

174.11
139.37 137.44 134.83
129.45 128.18
121.31 120.81 120.09
37.01
S
N
O
O=S=O
^{13}C NMR (101 MHz, 298 K, $CDCl_3$)
180 170 160 150 140 130 120 110 100 90 80 70 60 50 40 30 20 10 0
f1 (ppm)

γ-Lactam **23d**

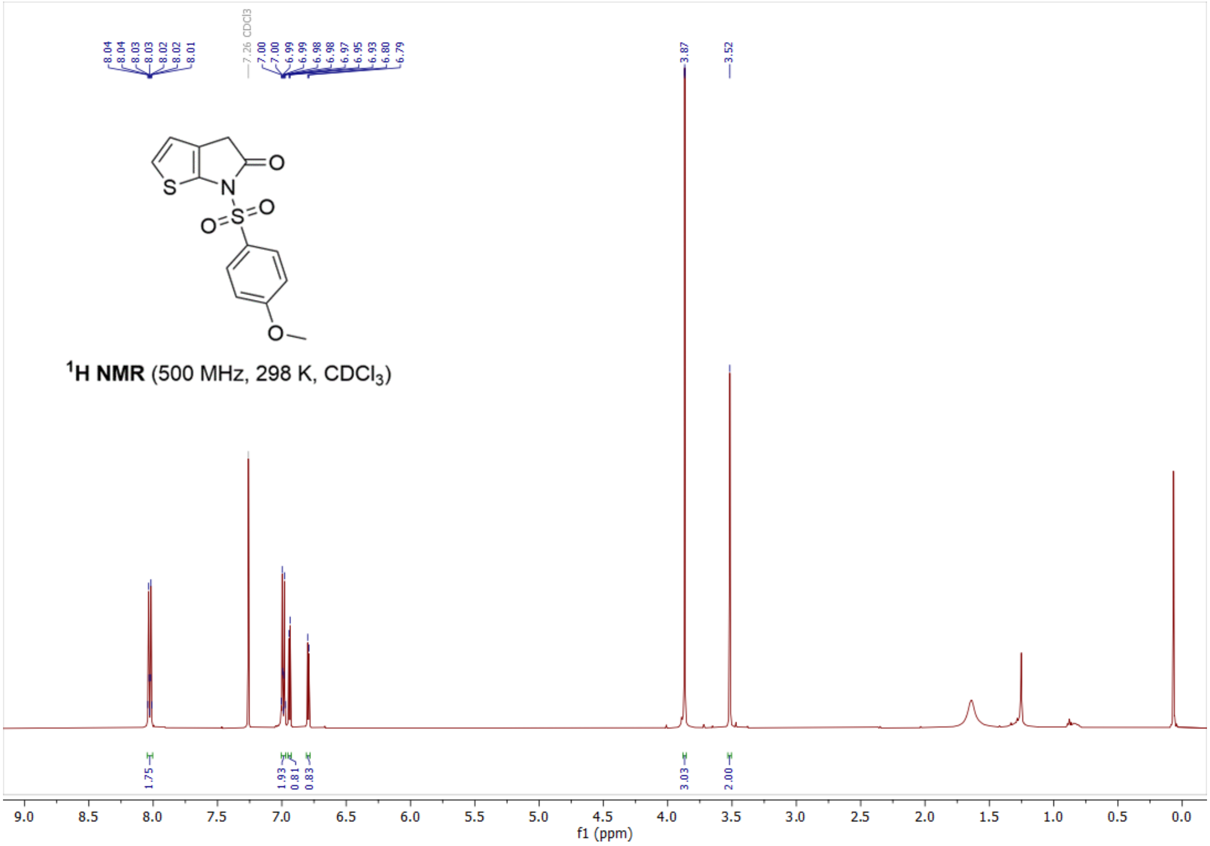

8.04
8.04
8.03
8.03
8.02
8.02
8.01
7.26 CDCl3
7.00
7.00
6.99
6.99
6.98
6.98
6.97
6.95
6.93
6.80
6.79
3.87
3.52
S
N
O
O
S
O
O
¹H NMR (500 MHz, 298 K, CDCl3)
1.75
1.93
0.81
0.83
3.03
2.00
9.0
8.5
8.0
7.5
7.0
6.5
6.0
5.5
5.0
4.5
4.0
3.5
3.0
2.5
2.0
1.5
1.0
0.5
0.0
f1 (ppm)

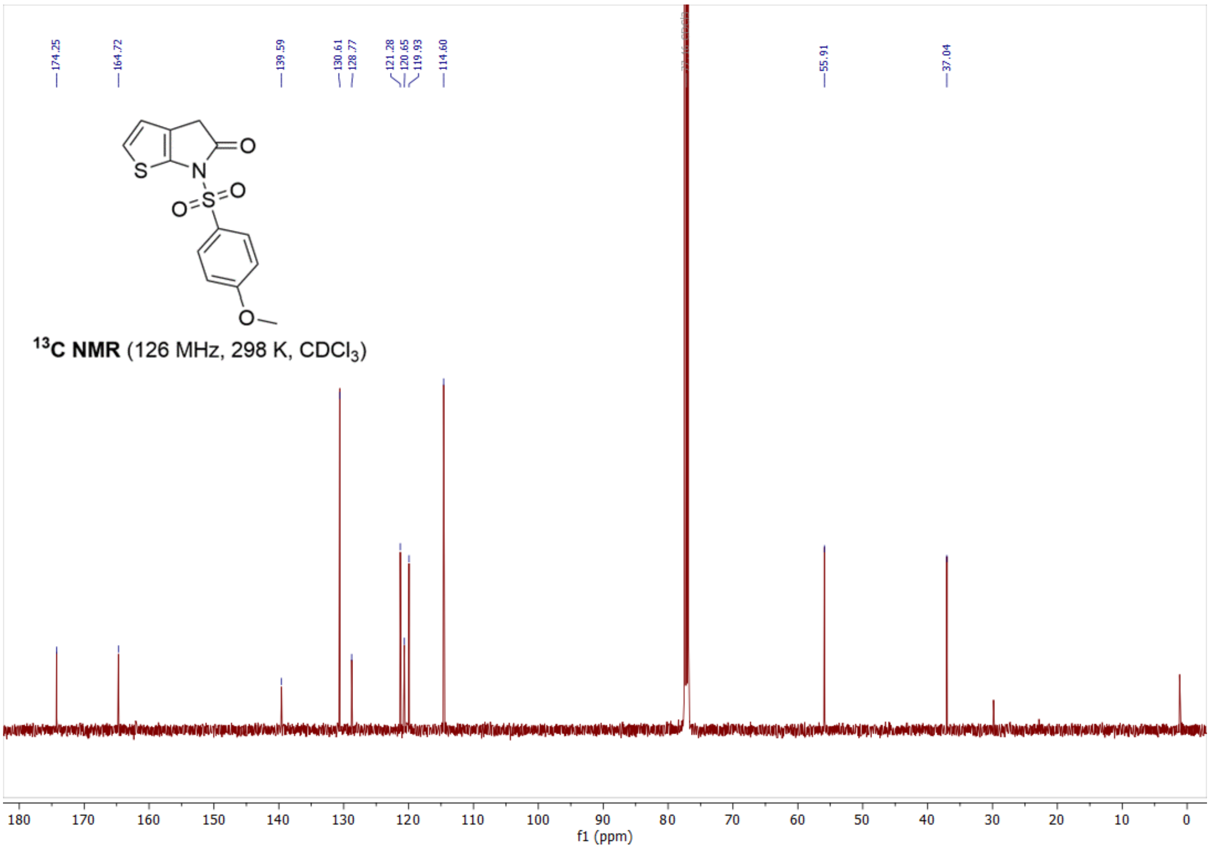

174.25
164.72
139.59
130.61
128.77
121.28
120.65
119.93
114.60
55.91
37.04
S
N
O
O
S
O
O
¹³C NMR (126 MHz, 298 K, CDCl3)
180
170
160
150
140
130
120
110
100
90
80
70
60
50
40
30
20
10
0
f1 (ppm)

γ-Lactam **23e**

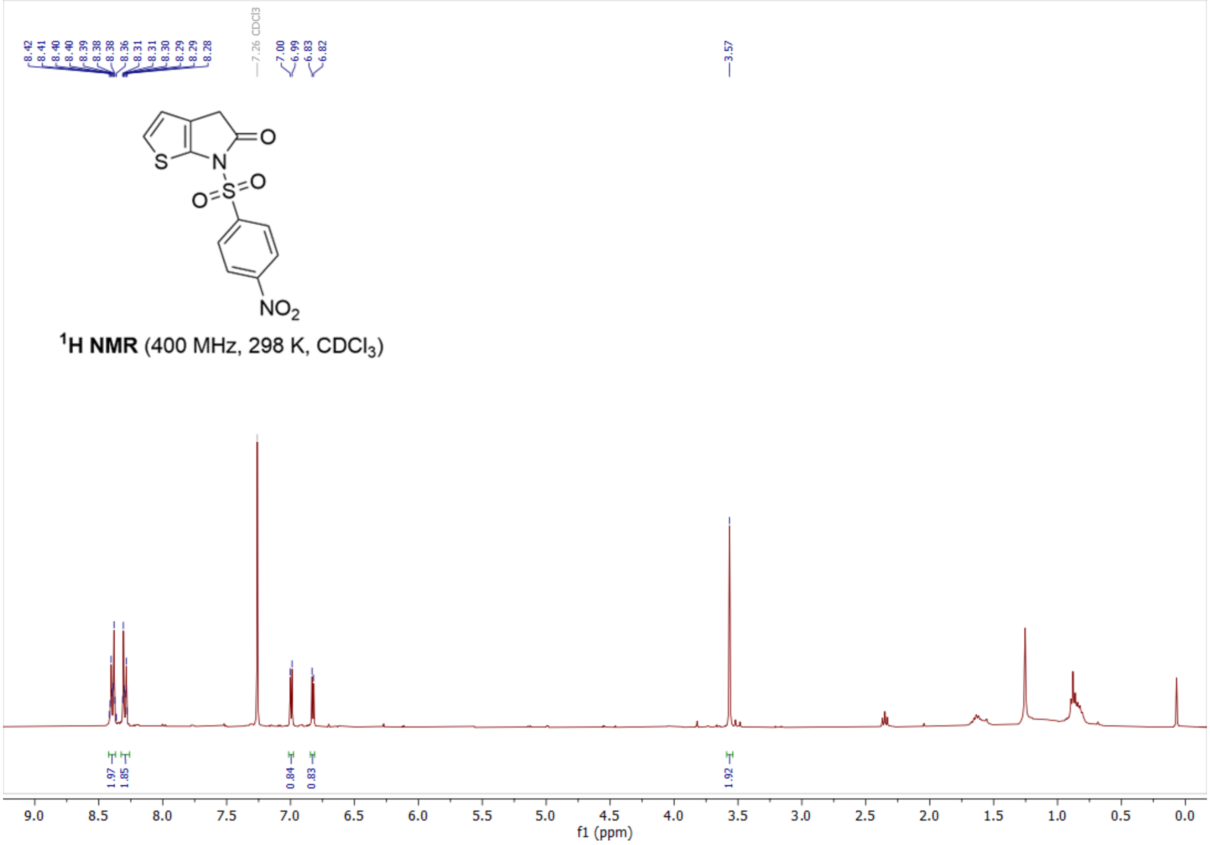

8.42
8.41
8.40
8.40
8.39
8.38
8.38
8.36
8.31
8.31
8.30
8.29
8.29
8.28
7.26 CDCl3
7.00
6.99
6.83
6.82
3.57
NO2
1H NMR (400 MHz, 298 K, CDCl3)
1.97
1.85
0.84
0.83
1.92
f1 (ppm)

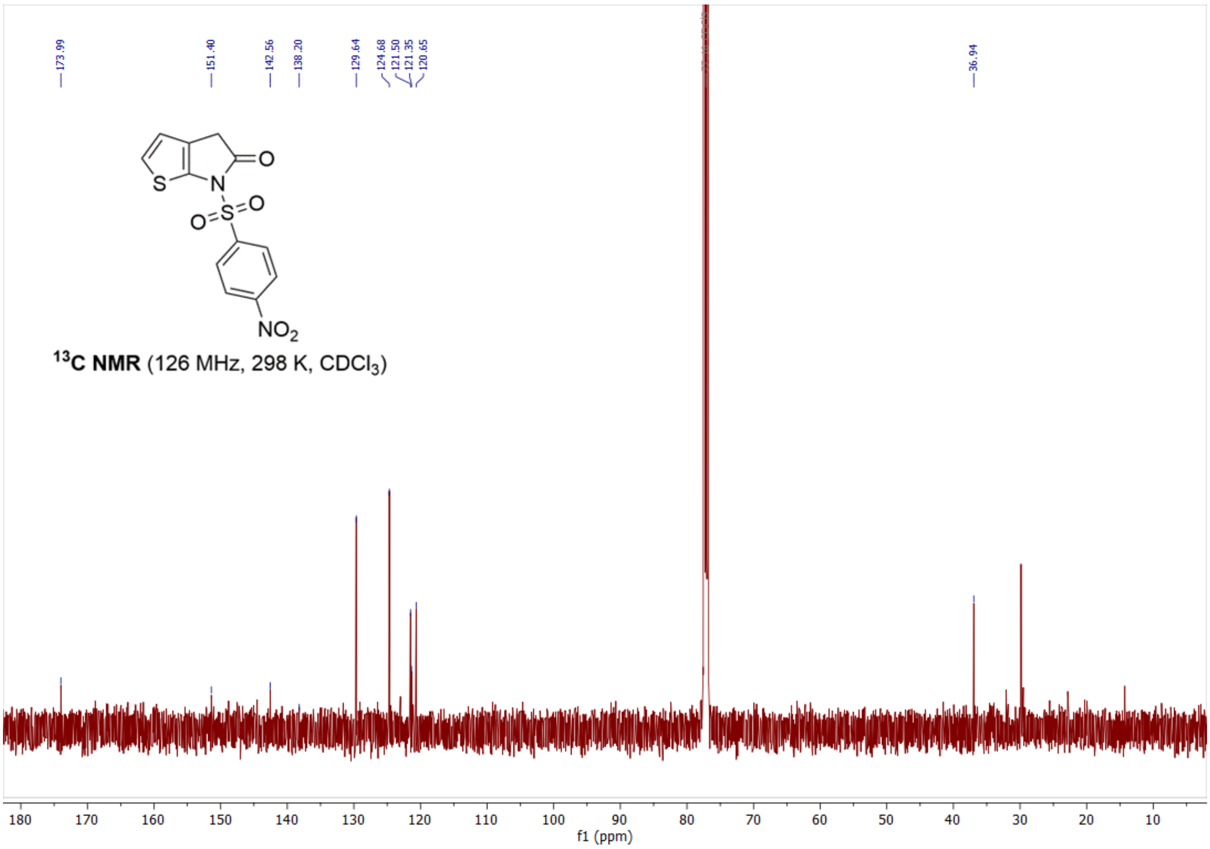

173.99
151.40
142.56
138.20
129.64
124.68
121.50
121.35
120.65
36.94
NO2
13C NMR (126 MHz, 298 K, CDCl3)
f1 (ppm)

γ-Lactam **23f**

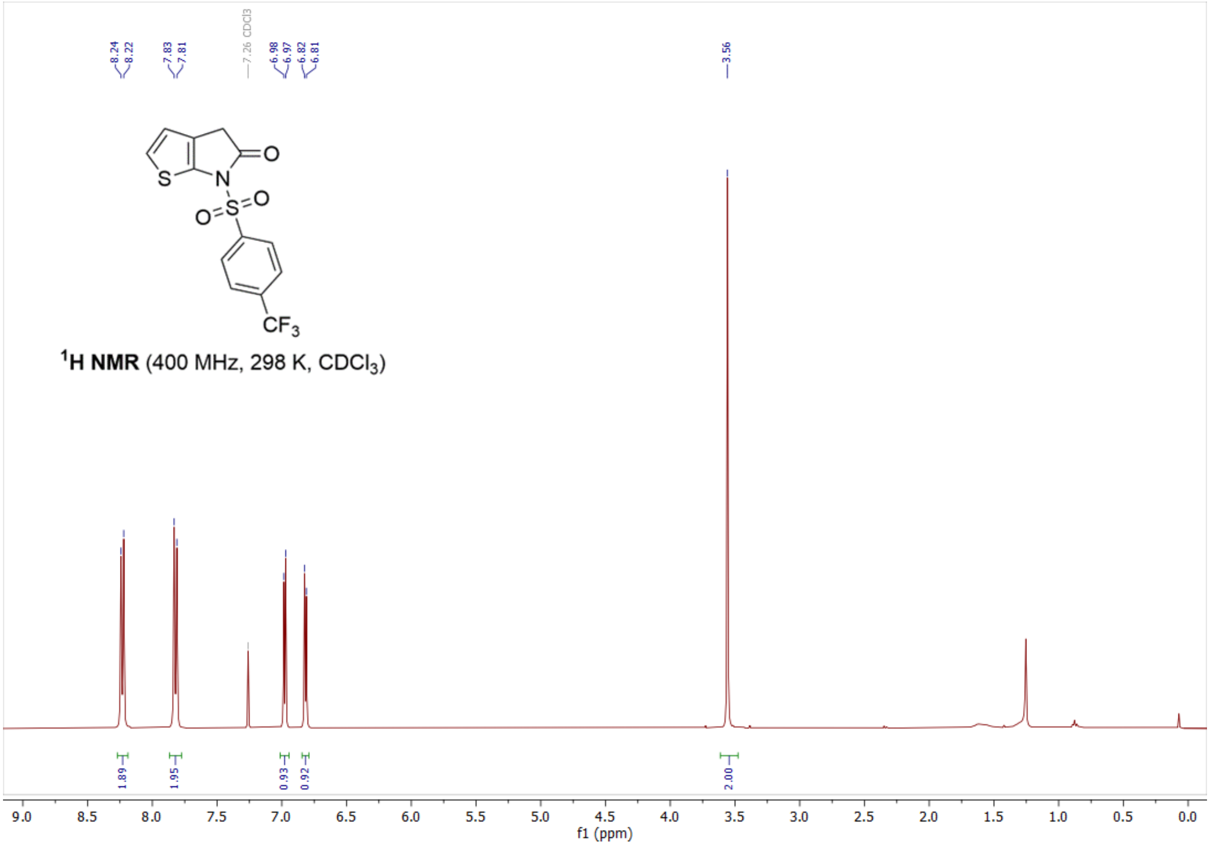

8.24
8.22
7.83
7.81
7.26 CDCl3
6.98
6.97
6.82
6.81
3.56
1H NMR (400 MHz, 298 K, CDCl3)
1.89
1.95
0.93
0.92
2.00
9.0 8.5 8.0 7.5 7.0 6.5 6.0 5.5 5.0 4.5 4.0 3.5 3.0 2.5 2.0 1.5 1.0 0.5 0.0
f1 (ppm)

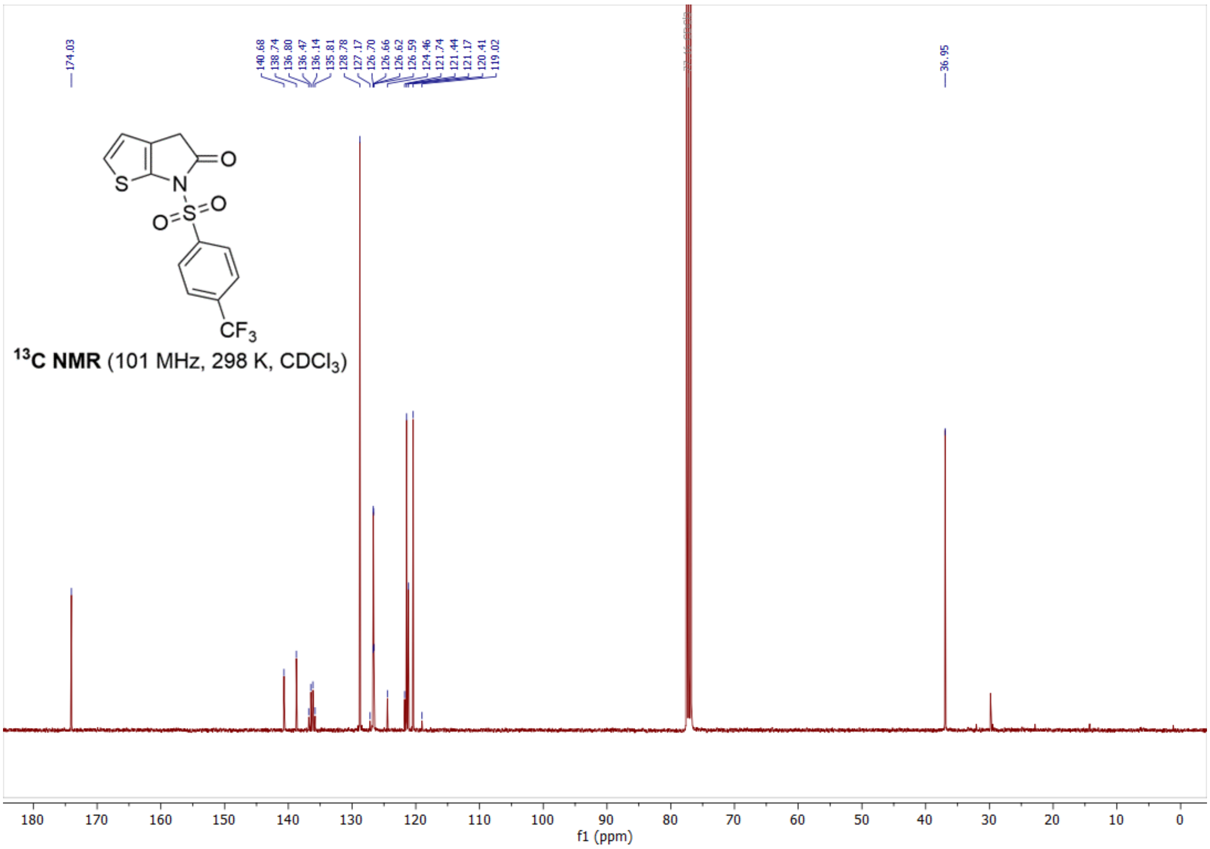

174.03
140.68
138.74
136.80
136.47
136.14
135.81
128.78
127.17
126.70
126.66
126.62
126.59
124.46
121.74
121.44
121.17
120.41
119.02
36.95
13C NMR (101 MHz, 298 K, CDCl3)
180 170 160 150 140 130 120 110 100 90 80 70 60 50 40 30 20 10 0
f1 (ppm)
